# Negative Autoregulation by FAS Mediates Robust Fetal Erythropoiesis

**DOI:** 10.1371/journal.pbio.0050252

**Published:** 2007-09-25

**Authors:** Merav Socolovsky, Michael Murrell, Ying Liu, Ramona Pop, Ermelinda Porpiglia, Andre Levchenko

**Affiliations:** 1 Department of Pediatrics, University of Massachusetts Medical School, Worcester, Massachusetts, United States of America; 2 Department of Cancer Biology, University of Massachusetts Medical School, Worcester, Massachusetts, United States of America; 3 Department of Biomedical Engineering, Johns Hopkins University, Baltimore, Maryland, United States of America; Institute for Systems Biology, United States of America

## Abstract

Tissue development is regulated by signaling networks that control developmental rate and determine ultimate tissue mass. Here we present a novel computational algorithm used to identify regulatory feedback and feedforward interactions between progenitors in developing erythroid tissue. The algorithm makes use of dynamic measurements of red cell progenitors between embryonic days 12 and 15 in the mouse. It selects for intercellular interactions that reproduce the erythroid developmental process and endow it with robustness to external perturbations. This analysis predicts that negative autoregulatory interactions arise between early erythroblasts of similar maturation stage. By studying embryos mutant for the death receptor FAS, or for its ligand, FASL, and by measuring the rate of FAS-mediated apoptosis in vivo, we show that FAS and FASL are pivotal negative regulators of fetal erythropoiesis, in the manner predicted by the computational model. We suggest that apoptosis in erythroid development mediates robust homeostasis regulating the number of red blood cells reaching maturity.

## Author Summary

The factors that control the rate of tissue growth during development are largely unknown. During embryogenesis, the formation of anucleated red blood cells (erythropoiesis) begins in the liver, with a dramatic expansion in erythropoietic tissue mass, occurring ten times faster than overall embryonic growth. We hypothesized that a network of cell–cell interactions within the erythroid microenvironment regulates this growth burst. To identify these regulatory interactions, we made use of the empirical finding that developmental processes are relatively robust to environmental perturbations. We determined how the frequency of erythroid progenitors in each of four sequential differentiation states varies during early development in vivo. We then modeled this behavior, and computationally selected those interactions that endow the network with resistance to external perturbations. This analysis predicted that erythroblasts in “state 2” of differentiation negatively regulate each other. We found that this autoregulatory interaction is mediated by the death receptor FAS and its ligand, FASL, which are co-expressed in state 2 cells. FAS-mediated cell death occurs only when the frequency of state 2 cells is high enough to permit their sufficient proximity. In this manner, FAS-mediated apoptosis dampens the initially rapid expansion of state 2 cells, and buffers unexpected fluctuations in their number, contributing to the system's robustness. We propose that a similar approach could be used to identify intercellular interactions in other rapidly growing tissues.

## Abstract 3

A novel computational algorithm is used to identify regulatory feedback and feedforward interactions between progenitors in developing erythroid tissue.

## Introduction

During development, progenitors undergo orderly differentiation through a series of maturation steps. The resulting number of fully differentiated progeny is precisely regulated to match physiologic and developmental needs, and is relatively resistant to environmental or gene-dose fluctuations [1,2]. This precise quantitative regulation of tissue development has been attributed to signaling interactions between cells within the tissue microenvironment [3,4]. Some of these interactions may be autoregulatory, taking place between progenitors of the same lineage. Although elucidating such regulatory intercellular networks is crucial to our understanding of development, little effort has been dedicated to the development of computational algorithms that would allow their reconstruction in a structured fashion. Here we propose such an algorithm and apply it to the intercellular network regulating progenitors of the non-nucleated red cell lineage (definitive, or adult-type, erythropoiesis), which first develops in the mouse fetal liver between embryonic day 12 (E12) and E15 [5]. The rapid production of red cells at this stage is dependent on the hormone erythropoietin (EPO) [6–8] and is essential for embryo survival, as shown by the death, between E13 and E15, of a number of mouse mutants defective for the formation of this lineage [6,9–13]. In addition to EPO, multiple soluble factors as well as direct intercellular interactions within the erythroid microenvironment have been implicated as potential erythroid regulators [14–18]. However, it is not clear how this plethora of candidate regulators is integrated into a coherent intercellular signaling network. Here we aimed to develop an algorithm that would identify the principal regulatory intercellular interactions that affect erythroid progenitors and that ultimately determine erythropoietic tissue mass and developmental rate. The underlying assumption of this analysis is that cells obey certain input–output relationship functions converting external signals into the probability of transition into the next developmental state.

An efficient identification of the main components of a regulatory developmental network requires quantitative measurements in developing tissue, in conjunction with mathematical modeling. The quantitative study of erythropoiesis was made possible recently by the development of a flow-cytometric assay that defines differentiation-stage-specific erythroblast subsets in erythropoietic tissue in vivo [19]. This assay allowed us to measure the dynamic changes in the frequency of these cells as they appear and differentiate in fetal liver. We then sought to develop a mathematical algorithm that would identify the principal intercellular interactions between erythroblasts during this developmental process.

A variety of mathematical tools have been employed recently to reconstruct regulatory intracellular networks from experimental datasets. These include statistical correlation techniques, such as Bayesian inference, used to model gene expression (e.g., in [20,21]) and signal transduction networks [22–24], or differential-equation-based models, used to reconstruct biochemical, gene expression, and signaling networks [25–29]. Most of these mathematical approaches aim to identify a model that best represents a biological network, often by first defining a space of all possible models, and then ranking these models based on their ability to fit the experimental data. Alternatively, though less frequently, algorithms seek to rank individual links within a network, without making definitive statements about the underlying network itself. Unfortunately, many of the currently used network reconstruction algorithms place important limitations on the state or structure of the network under analysis. For example, models based on ordinary differential equations (ODEs) frequently assume that the network is in steady state. Traditional Bayesian approaches impose limitations on network structure since they only allow networks devoid of cycles or feedback links. Although dynamic Bayesian approaches can overcome this limitation by using sufficiently frequent sampling of the network states, it may nevertheless be difficult to apply such approaches to developmental networks, which are dynamic, are far from equilibrium, and might not allow frequent sampling.

In this study, we developed a novel algorithm that focuses on identifying the principal intercellular interactions between erythroblasts, without necessarily determining the complete regulatory developmental network. Rather than ranking models representing the whole network, we ranked the network links: individual feedforward or feedback interactions between erythroblasts arising in developmental time course. To operate under relative scarcity of the initial experimental data and to increase the confidence in the ultimate results, interactions were ranked based on several criteria, including their ability to endow the network with robustness to small perturbations of the strength of the network links. We selected robustness as one of the ranking criteria since many biological processes are found to be relatively resistant to small exogenous perturbations [4,30–33].

Our proposed algorithm contains several steps. The first consists of the acquisition of biological data describing the developmental process, in terms of time-dependent changes in developmental markers. In the second step the developmental process is defined in terms of a series of discrete states. In the third step, a generalized model is constructed, describing how regulatory interactions between cells in different states may be responsible for the time-dependent changes that are observed experimentally. In the fourth step, different model networks, or topologies, are generated, based on the generalized model developed in the previous step. In the fifth step, the goodness of fit and robustness properties associated with each model network are characterized, as well as the values of model parameters and the ability of each parameter to influence the model fit. In the sixth step, likely feedback and feedforward interactions are identified, based on whether they occur in the model networks that are more robust to parameter variation and that show better fit to the experimental data. In the seventh step, we identify candidate molecules that may regulate the biological process and mediate the interactions predicted by the algorithm. In the final step we examine quantitatively the role of these candidate molecules during the developmental process in wild-type mice and in relevant mutant mice, in order to ascertain that their function indeed matches that predicted by the algorithm.

Application of this algorithm to fetal erythropoiesis identified a negative autoregulatory interaction, between erythroblasts of similar maturation stage, as highly significant in the homeostatic regulation of erythroid development. We show that this interaction is exerted through the death receptor FAS and its ligand, FASL.

## Results

### Definition of Developmental States in Fetal Liver, Based on Expression of CD71 and Ter119

**Figure 1:**
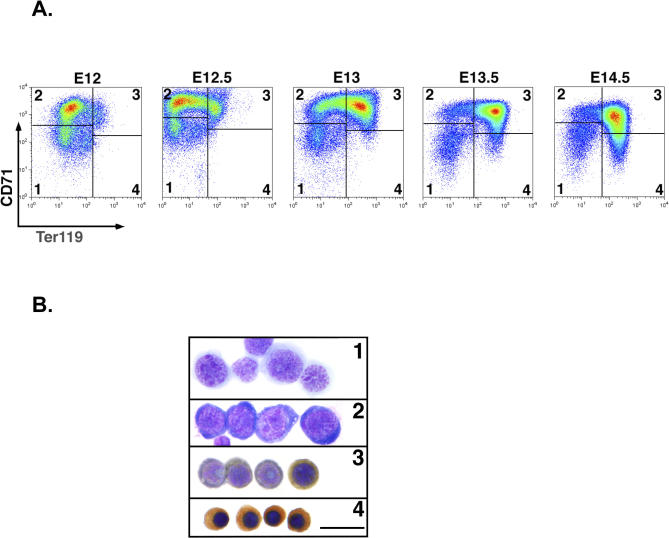
Erythropoietic Developmental Wave in Fetal Liver (A) CD71/Ter119 flow-cytometric histograms of fetal liver cells derived from embryos between E12 and E14.5. Developmental states 1 to 4 are noted. (B) Cytospin preparations of E14.5 fetal liver cells sorted from each of states 1 to 4 show increasingly differentiated erythroid progenitors. Cells were stained for hemoglobin expression (brown) with diaminobenzidine and counterstained with Giemsa. Scale bar = 20 μm. Developmental states 1 to 4 are defined as described in the text and in Protocol S1, section 1.1.

Fetal liver between E12 and E15 is primarily an erythropoietic tissue, although it also contains a small minority of non-erythroid cells. We divided all fetal liver cells, both erythroid and non-erythroid, into four developmental states, and then measured the relative frequencies of cells in each state as a function of time in the following manner. We used a recently developed flow-cytometric assay that makes use of the cell-surface markers CD71 (also known as TFRC) and Ter119 (also known as LY76) to identify differentiation-stage-specific erythroblasts in erythropoietic tissue [19]. Ter119 is an erythroblast-specific epitope, first expressed at the proerythroblast stage and throughout subsequent maturation [34,35]. The transferrin receptor, CD71, is expressed at high levels in proerythroblasts and is gradually down-regulated with differentiation [36]. Two-dimensional flow-cytometry histograms of fetal liver at the onset of erythropoiesis (Figure 1) show that cells accumulate in distinct regions of the CD71/Ter119 expression “space.” Based on this and on morphological and other criteria, we divided fetal liver cells into four states, with progression from state 1 to 4 representing a developmental sequence (Figure 1; Protocol S1, section 1.1; [19,37]). State 1 cells express moderate levels of CD71 and are negative for Ter119 (CD71^med^Ter119^neg^). State 1 contains the earliest erythroid precursors. It also contains all the non-erythroid cells in fetal liver, which constitute 10% or less of all state 1 cells [19,37]. State 2 cells are CD71^high^Ter119^low^, state 3 cells are CD71^high^Ter119^high^, and state 4 cells are CD71^med/low^Ter119^high^. All cells in states 2, 3, and 4 are in the erythroid lineage [19,37].

Analysis of fetal liver progenitors on successive days of development (E12–E15.5) reveals a “developmental wave,” with a large number of progenitors appearing first in states 1 and 2, and progressing into states 3 and 4 with developmental age (Figure 1A). We used this initial dataset indicating relative (fractional) numbers of cells in different states over developmental time in our further analysis.

### Generalized Model Development

We next constructed a generalized model that describes how regulatory intercellular interactions may be responsible for generating the experimentally observed erythropoietic developmental wave in Figure 1. A system of ODEs was used to describe how the proportion of cells in each state *i* (where *i* denotes any of states 1 to 4) changes with time (equation 1). At each developmental time point, the flow-cytometric data show how fetal liver cells are distributed amongst the four states, but does not provide absolute cell numbers. Therefore, the state variables, *x_i_*, represent the proportion of all cells in each state, rather than absolute cell numbers, and their sum at any particular time always adds up to one. Conversion into absolute cell numbers is possible by multiplying *x_i_* by the number of cells in fetal liver at each developmental stage (Protocol S2, section 2.1).

We assumed that, within the time scale considered here, the ODE system is driven by its initial conditions, with no additional influx of cells to state 1 nor efflux from state 4. We also assumed that no “de-differentiation” occurs and that cells transition unidirectionally from state 1 to 2, 2 to 3, and 3 to 4. Therefore, each transition rate *h_i_*_,*i+*1_ from state *i* to state *i+*1 is non-negative. Equation 1 describes how each state variable *x_i_* changes with time, as a result of influx from the preceding state and/or efflux into the subsequent state:

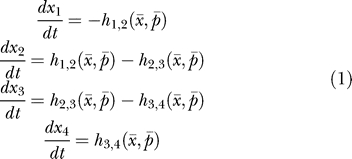



The transition functions *h_i_*_,*i+*1_ govern the rates of transition of cells from the “source” state, *x_i_*, to the immediately subsequent state, *x_i+_*_1_ *.* These functions consist of two terms, each describing a distinct type of signaling interaction (equation 2). The first is a time-dependent progression of the differentiation process in each cell, facilitated by the hormone EPO. It causes transition of cells between states that is independent of intercellular interactions between progenitors, and is therefore described as a first-order state dependency. Three non-negative parameters, *p*_12_ *, p*_23_, and *p*_34_, represent the rates of transition per cell due to this process from states 1 to 2, 2 to 3, and 3 to 4, respectively. The transition function *h_i_*_,*i+*1_ is also influenced by a second type of interaction, due to feedback or feedforward regulation of cells in the source state, *x_i_* , by cells in any other state, *x_j_* . The effect of such an interaction on the transition function may be either positive or negative, and is proportional to the product of the fractions of cells in the two interacting states, *x_i_x_j_*. In the special case where *j = i,* the interaction is autoregulatory and is proportional to *x_i_* ^2^ *.* Twelve parameters, *p_k_* (*k* ∈ 4*–*15), represent the rate constants for each of these potential interactions, which are classified as feedforward (ff[*j*][*i+*1], if *j* ≤ *i*) or feedback (fb[*j*][*i*], if *j* > *i*) (detailed in Protocol S1; note that *p_ij_* in Protocol S1 is denoted as *p_i_* for all *i*). Note that, since we rely on the fractional rather than total numbers of cells in specific states (because of the nature of data obtained in flow cytometry), the sum of fractional cell numbers is always unity. The transition function *h_i_*_,*i+*1_ incorporates all these potential interactions:

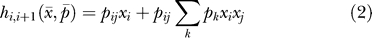



### Note on the Mathematical Model

The model contained in equations 1 and 2 is an abstract approximation of what are in reality much more complex processes. Specifically, the transition functions *h_i−1_*_,*i*_ and *h_i_*_,*i+*1_ combine processes of cell differentiation, proliferation, and death, all of which regulate the fractional number of cells in state *i*. Our use of a more abstract representation was driven primarily by the desire to make the algorithm applicable to cases where direct and independent measurements of cell number, cell division, and apoptotic rate might be hard to obtain, which will likely be true for most developmental processes. Introduction of a more detailed description of cell division and cell death would require estimation of a higher number of free parameters and thus, potentially, a greater number of initial measurements, which, in turn, may require artificial perturbations of the developmental network. However, we can foresee that much more detailed models will be used in the future as our ability to measure in vivo states of developmental networks increases. Nevertheless, since any model is likely to be less detailed than the underlying system, the utility of the model is primarily in its predictive power. We show below that the description contained in equations 1 and 2 is sufficient to make predictions as to the nature of a pivotal regulatory interaction and the developmental stage at which it occurs, both validated experimentally. One can thus claim that the use of the particular form of this mathematical model was justified a posteriori.

### Generation of Model Topologies

**Figure 2:**
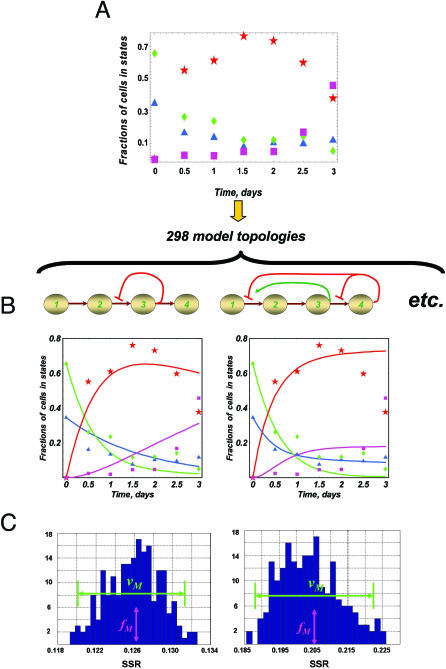
Generation and Analysis of the 298 Distinct Model Topologies Used in the Computational Analysis (A) The relative numbers of cells in four states over the time course of the erythropoietic wave shown in Figure 1 were evaluated (Protocol S1, section 1.1) and plotted versus time. The fractions of cells in state 1 (blue triangles), 2 (green rhombs), 3 (red stars), and 4 (magenta squares) are shown. (B) The model topologies were generated by allowing up to three independent feedback and feedforward interactions between different states, so that the fractions of cells in some of the states might affect the transition probabilities between pairs of successive states. Each model topology was then fitted to the data shown in (A). Two examples of the possible 298 topologies and the corresponding best fits are shown. (C) For each model topology, the parameters determined during the fitting process were then varied within a 5% range, resulting in 200 predictions for the corresponding model topology, each with its associated SSR. The distributions of SSR values for two such model topologies are shown. The mean SSR value, *f_M_*, is computed, as well as its variance in the distribution, *v_M_*. These values are recorded for all the model topologies and then used to calculate the *F_j_* and *R_j_* metrics for each of the possible feedback and feedforward interactions, shown in Figure 4.

We generated different model networks, or topologies, based on the general model described above. Erythroblast differentiation due to EPO action, represented by the first-order state dependency term and the parameter *p_ij_* (*i* ∈ 1, 2, 3; *j* ∈ 2, 3, 4), is always present in the transition functions (equation 2). However, we limited the number of feedback and feedforward interactions that exist simultaneously in the transition functions of any one model to three or less. This assumption was based on the desire to limit the combinatorial explosion in the number of possible state dependencies, and on the more general argument that multiple and dense interactions between elements of any network can lead to instabilities [38]. In a similar fashion, a recent network reconstruction analysis considerably limited the number of possible links in potential networks, obtaining nevertheless excellent results [29]. The feedforward and/or feedback interactions that exist simultaneously in any particular model determine its topology or connectivity (Figure 2). Thus, all potential model topologies containing three or fewer such interactions were considered, giving a total of 298 models (Figure 2; Protocol S1).

**Figure 4:**
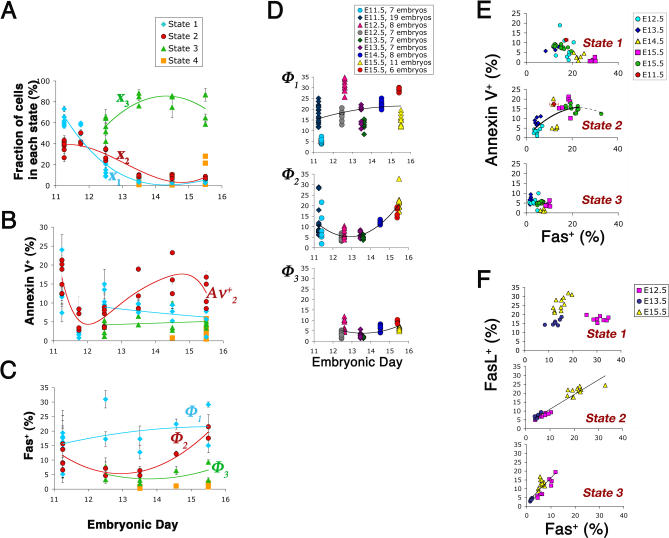
Fraction of Cells Expressing FAS or FASL or Binding ANXA5 during E11–E15.5 (A and B) ANXA5 binding in each of the four fetal liver states (B) and the corresponding frequency of each state in the same fetal livers (A) during E11–E15.5. Each data point is the mean ± standard error of the mean for embryos in one litter. *x*_1_, *x*_2_, and *x*_3_ are the fraction of cells in states 1, 2, and 3, respectively. Av^+^_2_ is the fraction of cells in state 2 that bind ANXA5. Individual embryo data for the same dataset are presented in Protocol S2, section 2.3. (C and D) The fraction of cells expressing FAS in each state during E11–E15.5. Each data point in (C) is mean ± standard deviation for a litter of embryos. Individual embryos in the same dataset are shown in (D); each symbol denotes an independent experiment of one or more litters from the same embryonic age. Further analysis of embryos in this dataset, with the same symbols, is shown in Figure 6A and 6F. Φ_1_, Φ_2_, and Φ_3_ are the fraction of cells expressing FAS in states 1, 2, and 3, respectively. All curves are second-order polynomials. In order to avoid overlapping data points in (D), the data for some litters were slightly displaced along the horizontal axis (e.g., E13.5 was plotted as E13.45). (E) Fraction of cells that are ANXA5^+^ and FAS^+^ in states 1 to 3, measured in the same fetal livers (E12.5–E15.5) or in the same litters (E11.5, due to the small number of cells in fetal liver at this age). Each point represents a single embryo (E12.5–E15.5) or a single experiment (E11.5, mean of four litters). Each symbol denotes an independent experiment of one or more litters. Curve is a second-order polynomial. (F) Fraction of cells that are FASL^+^ and FAS^+^ in states 1 to 3, measured in the same fetal livers (E12.5–E15.5). Each color denotes an independent experiment of one or more litters.

**Figure 6:**
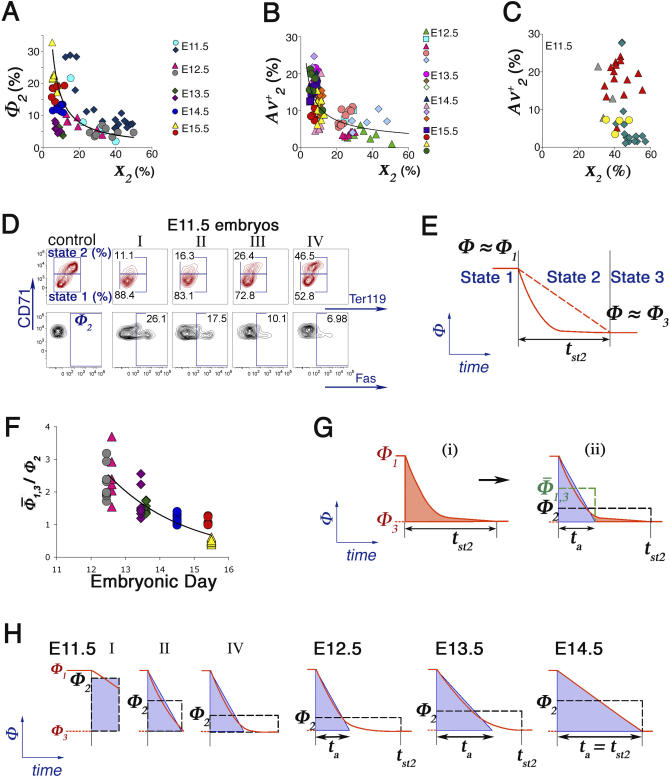
The Rate of FAS-Mediated Apoptosis in State 2 Is Proportional to *x*_2_^2^ (A) The fraction of FAS^+^ cells in state 2 (Φ_2_) is inversely proportional to the fraction of all fetal liver cells that are in state 2 (*x*_2_). Each point represents an individual embryo from the same set of embryos shown in Figure 4D. The curve describes the equation *y =* 162*x^−^* ^1*.*009^ (*R* ^2^ = 0.63). (B and C) The fraction of ANXA5^+^ cells in state 2 (Av^+^_2_) is inversely proportional to *x*_2_ between E12.5 and E15.5 (B), but not in earlier E11.5 embryos (C). Each point represents an individual embryo from the same set of embryos and with the same symbols as in Protocol S2, section 2.3. The curve describes the equation *y =* 0.03*x^−^* ^0*.*6^ (*R* ^2^ = 0.4). (D) Four E11.5 embryos from the same litter, showing the distribution of cells in states 1 (*x*_1_) and 2 (*x*_2_) in the top panels, and a fraction of state 2 cells that are FAS^+^ (Φ_2_) in the lower panels. Staining control for FAS shown in the leftmost panels. See additional data on these in Protocol S2, section 2.4. (E) Potential time course of Φ, the fraction of cells that are FAS^+^, in a cohort of erythroblasts traversing state 2. *t*_st2_ is the length of state 2. Φ_1_ and Φ_3_ are used as estimates for Φ when cells enter and exit state 2, respectively. The dashed red line represents a constant rate of decline in Φ throughout state 2. The solid red curve represents an alternative time course, with an initial rapid decline in Φ that slows down due to depletion of FAS^+^ cells. (F) Comparison of Φ_2_ with Φ_1,3_, the arithmetic mean of Φ_1_ and Φ_3_, during E12.5 and E15.5. Data calculated for each embryo from values shown in Figure 4D. If the rate of decline in Φ in state 2 were constant, the ratio Φ_1,3_/Φ_2_ would be expected to be equal to one. Since Φ_1,3_/Φ_2_ is greater than one for embryos younger than E14.5, the rate of decline in Φ in these embryos resembles the solid red curve in (E). (G) Approximate method for relating Φ_2_ to Φ_1,3_ when the rate of decline in Φ is nonlinear. Φ_2_ equals the area under the red Φ curve (shaded red, panel i) divided by *t*_st2_, and may be represented as the height of a rectangle (black dashed lines, panel ii) of height Φ_2_ and base *t*_st2_. The shaded red area is approximately equal to the area of a triangle (shaded blue, panel ii) of base *t*_a_, where *t*_a_ is the length of time within state 2 when the rate of decline in Φ is relatively constant. The area of the blue triangle, given by Φ_1,3_ *t*_a_, is therefore approximately equal to the area of the black dashed rectangle: Φ_2_ *t*_st2_ = Φ_1,3_ *t*_a_ *.* (H) Graphical representation of Φ_2_ and how it relates to the rate of decline in Φ. At E11.5, Φ declines rapidly as the first cohort of erythroblasts progresses through state 2, as suggested by data in (A) and (D). At E12.5–E13.5, Φ declines sufficiently rapidly so that it plateaus prior to completion of state 2, as suggested by (F). By E14.5, the rate of decline has slowed down and remains constant throughout state 2. Throughout, Φ_2_ is directly proportional to *t*_a_ (from [G]), and both are inversely related to *x*_2_ (from [A], and see text).

### Model Fitting and Selection of Likely Feedback and Feedforward Interactions

Each model topology was described as an ODE system corresponding to a unique set of feedback and feedforward interactions (Figure 2A). The numerical values for each of the *p_i_* and *p_k_* parameters in a given model were obtained by fitting of the model to the experimental data (Figure 2B; Protocol S1, section 2.3). The constraints on the model-fitting process were that the difference between all experimental and simulated data would be minimized, and that the values of all state transitions, state variables, and first-order transition parameters, *p_i_*, would be non-negative. We opted to allow the sign (whether positive or negative) of the nonlinear transition parameters, *p_k_,* to be determined by data regression.

Rather than describe a specific erythroid intercellular interaction network, our aim was to identify individual, significant feedback or feedforward interactions that are critical to the network's regulation, regardless of the network's final, complete form. With this aim in mind, we developed a comprehensive analysis of the 298 model topologies. In particular, rather than rank specific model topologies, we ranked the 12 feedback and feedforward interactions that constitute the models. Each of the 12 feedback/feedforward interactions participates in 67 of the 298 models we tested, either by itself, or in combination with one or two other interactions. We devised four criteria, or metrics, that test the performance of each interaction within the context of each of the 67 model topologies in which it participates.

We assumed that an individual interaction, if important, would contribute substantially to the dynamic output of the network, as well as to network robustness. This assumption was based on observations suggesting that single feedback or feedforward interactions may significantly influence the dynamic output of biological networks, such as oscillations or switch-like behavior [39–43]. The four criteria we used to identify potentially significant interactions were: fitness, robustness, consistency, and control. The fitness property requires that an interaction be present more frequently in the subset of models displaying higher fit to experimental data. The robustness property requires that it also be overrepresented in models whose fit to the data is robust to parameter variations. The consistency property requires that the parameter *p_k_* describing a significant interaction have a consistent sign, either positive or negative, and that its magnitude be distributed within a relatively narrow range, across all the models in which it is present. Finally, the control property suggests that variations in the parameter *p_k_* describing a significant interaction should strongly influence the fit of the model to the data. An interaction where this is not the case would not be expected to be of much consequence to determining the network output and thus the experimental dataset.

To examine these properties for each potential feedforward and feedback interaction, we carried out multivariate analysis on each of the 298 models (Protocol S1, section 2.4). We first measured the goodness of fit of each model prediction to the experimental data, expressed as the sum of squared residuals (SSR; Figure 2B), using the optimal numerical values for each of the model parameters. We then varied all of the parameters in a given model simultaneously, assigning each parameter a random value within a 5% range of the optimal fitted value. The goodness of fit of the resulting new model prediction was again measured, yielding a new SSR value. This analysis was repeated 200 times for each model topology, yielding 200 predictions, each with its associated SSR value. Therefore, in all, we analyzed 298 × 200 = 59,600 unique parameter sets. We plotted the set of SSR values for each model in the form of a histogram (Figure 2C). We postulated that the variance of the histogram, *v_M_,* is inversely related to the robustness of a model to parameter variations. The mean value of the SSR histogram, *f_M_,* is inversely related to the goodness of fit of the model predictions to the experimental data.

**Figure 3:**
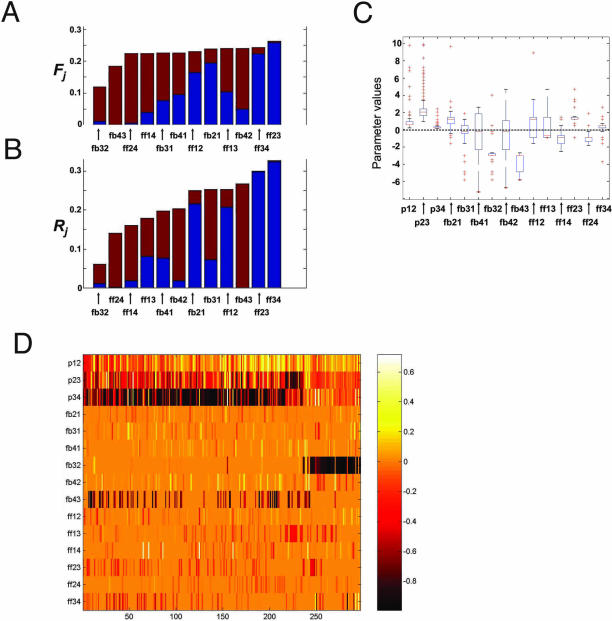
The Possible Feedforward and Feedback Interactions between Developmental States Are Analyzed by Applying Fitness, Robustness, Consistency, and Control Criteria (A) All possible feedforward and feedback interactions are rank-ordered according to whether they are mostly present in the models of higher fit to the data in Figure 2A. Specifically, the fitness metric *F_j_* is calculated for each interaction *j* by summing the inverse values of *v_M_*, determined as described in Figure 2C, over all models *M* where the interaction *j* is present. The models are then ranked according to the values of *F_j_*. (B) All possible feedforward and feedback interactions are rank-ordered according to whether they are mostly present in the models of higher robustness. Specifically, the robustness to parameter variation metric *R_j_* is calculated for each interaction *j* by summing the inverse values of *f_M_* , determined as described in Figure 2C, over all models *M* where the interaction *j* is present. The models are then ranked according to the values of *R_j_*. (C) The consistency of parameter values corresponding to particular feedforward and feedback interactions is evaluated by plotting the parameter values for each model *M* in which the corresponding interaction is present. Each box has lines at the lower quartile (blue), median (red), and upper quartile (blue) values. The whiskers show the extent of the rest of the data. Outliers (red plus signs beyond the ends of the whiskers) indicate data with values more than 1.5 times the interquartile range away from the top or bottom of the box. (D) A correlation matrix relating the control exercised by each feedback or feedforward interaction in each model in which it is present. Each row in the matrix corresponds to a single parameter, describing a single type of interaction. Each column represents one specific model topology. Models are arranged from left to right in descending order of their best fit to the experimental dataset. The color bar indicates the value of the control metric, defined as the correlation coefficient between the parameter values and their associated SSR values, obtained during the multivariate analysis illustrated in Figure 2C. Note that for ff23, ff34, and fb43 interactions, there is clustering of high negative correlation values for high-ranked models, suggesting the importance of these interactions in influencing the fitness of the high-ranked models.

We used the results of this multivariate analysis to evaluate the role of each of the potential feedforward and feedback interactions in the network. We first asked whether certain interactions occur more frequently in models with better fitness and/or higher robustness. We examined this by calculating, for each interaction *j,* a goodness of fit metric, defined as 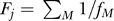 , where the summation is over all the models *M* (where *M* = 67) in which this particular interaction *j* is present (Figure 3A). Similarly, we calculated for each interaction a robustness metric, defined as 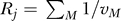 (Figure 3B). We next evaluated the consistency of each interaction by assessing the distribution of values of each parameter across the models in which the interaction it describes is present (Figure 3C). Finally, we assessed the extent to which each interaction controls a model's output, using the above multivariate analysis. For each interaction in each model, we calculated a correlation coefficient that relates the values assigned to the parameter in question in each of the 200 simulations to the resulting SSR values. The results of this analysis were compiled in a correlation matrix (Figure 3D).

### Interactions Emerging as Significant in the Erythroid Developmental Network

Of the twelve potential feedback and feedforward interactions we considered during the implementation of the algorithm, only two, ff23 and ff34, satisfied all the required criteria. Both assume a relatively narrow range of positive values and score high on the fitness, robustness, and control criteria (Figure 3A–3D). Of these, ff23 was estimated to be at least twice as strong as ff34. The discrimination provided by the fitness metric was relatively poor, since there was less than a 10% difference between the ten top-ranked interactions (Figure 3A). If we disregard the fitness criterion, an additional interaction, fb43, emerges as potentially significant, ranking high in terms of robustness and control, and assuming consistently strongly negative values (Figure 3B–3D).

We tested these conclusions by repeating the above analysis on altered datasets (Protocol S1, section 4). In particular, since the precise age of individual embryos can be determined only approximately, we tested the sensitivity of our analysis and conclusions using datasets in which the estimates of the time points in the original dataset were varied by ±0.4 d, or specific time points were interchanged. We also tested the effect of alteration of the details of the algorithm used (Protocol S1, section 5). In particular, we performed alternative analyses that relied more heavily on data in states 2 and 3, since cells in states 1 and 4 are present at lower numbers and only at the start and end of the developmental wave, respectively (Figure 1). We also explored if the predicted likelihood of specific interactions would be altered if larger (50%) perturbations in the parameter values were used in the robustness analysis. Throughout these different analyses, the interactions ff23, ff34, and fb43 emerged repeatedly as the most likely in the erythroid developmental network, with ff23 ranked high most consistently.

Both ff23 and ff34 represent a special case of feedforward interactions, where the source state regulates itself (ff[*j*][*i+*1], where *i = j*). The parameters describing ff23 and ff34 have consistently positive values (Figure 3C). In each case, therefore, the effect of these interactions is to increase transition from the source state (Protocol S1). The interaction ff23 will increase in proportion to the square of the relative number of cells in state 2. It will cause increased transition of cells from state 2, with a resulting decrease in the number of cells in state 2 relative to the number of cells in state 3. Therefore, ff23 will tend to limit the relative number of cells in state 2, and may be described as a negative autoregulatory interaction. The biological mechanisms giving rise to increased transition from state 2 may be either increased differentiation into state 3 or increased apoptosis of state 2 cells. The modeling procedure does not allow us to distinguish these two possibilities. Similar arguments apply to ff34, which is a negative autoregulatory interaction proportional to the square of the relative number of cells in state 3, tending to limit the relative number of cells in state 3.

The fb43 interaction has a negative parameter value, and its effect is therefore to decrease the transition from state 3 to state 4, in proportion to the product of the relative number of cells in states 3 and 4 (Protocol S1). The fb43 interaction may be expected to offset, or moderate, the effect of ff34.

### FAS Mediates Apoptosis of State 2 Cells

To begin to evaluate the biological plausibility of the predictions made by the computational algorithm, we first considered tissue architecture within fetal liver. Erythroblasts in tissue are found within anatomical units known as erythroblastic islands, where they form concentric rings around a central macrophage. We found that, at E15.5, 50% and 30% of all state 2 and 3 cells, respectively, are adjacent to cells of the same state (Protocol S2, section 2.2B). This architecture is therefore consistent with autoregulatory interactions between cells of the same state, such as ff23 or ff34.

Next, we considered candidate molecules that might mediate the ff23 or ff34 interactions. As indicated above, a likely underlying process described by these interactions is apoptosis arising from cell–cell interactions within the same developmental state. FAS is a cell-surface receptor of the tumor necrosis factor (TNF) receptor family. It triggers apoptosis when activated by its ligand, FASL, expressed on the surface of adjacent cells [44]. Although the role of FAS has been best described in immune cells, FAS is also expressed by many other cell types, including cultured erythroid cells [14,45]. Very recently, we found that a fraction of adult spleen, but not bone marrow, erythroblasts also express FAS and FASL [46]. However, the expression and potential function of FAS/FASL in fetal liver in vivo during the onset of erythropoiesis had not been determined. Indeed, although FAS and FASL are expressed by several embryonic tissues, to date no clear developmental role has emerged for these molecules [47].

We examined the possibility that FAS-mediated apoptosis, triggered by FASL on neighboring cells, may form the basis for the negative autoregulatory interactions predicted by our modeling. We measured FAS and FASL expression as well as an early marker of apoptosis, ANXA5 (Annexin V) binding, in fetal livers freshly isolated from embryos between E11.5 and E15.5. Figure 4 summarizes 23 independent experiments, examining 29 litters containing a total of 189 embryos. The relative number of cells within each state changes rapidly over this period (Figure 4A), consistent with the original dataset (Figure 2A). Associated with this change are rapid changes in ANXA5 binding and FAS expression, particularly in state 2, where the fraction of ANXA5^+^ cells (Av_2_^+^; Figure 4B; Protocol S2, section 2.3; glossary of symbols in Table 1) and the fraction of FAS^+^ cells (Φ_2_; Figure 4C and 4D) vary in a pattern that resembles an oscillation. There is a trough in both Av_2_^+^ and Φ_2_ at E12.5, coinciding with a peak in the relative number of cells in state 2 (*x*_2_; Figure 4A). Measurement of both FAS expression and ANXA5 binding in the same embryos showed Av_2_^+^ and Φ_2_ to be positively correlated (Figure 4E). Unlike in state 2, the fraction of ANXA5^+^ or FAS^+^ cells in states 1, 3, or 4 did not vary significantly with development (Figure 4B–4D), and there was no correlation between ANXA5 binding and FAS expression in these states (Figure 4E). These results suggest that FAS mediates apoptosis within state 2, but not other states. We also noted that, throughout development, FASL expression within states 2 and 3 closely correlated with FAS expression (Figure 4F).

**Table 1:**
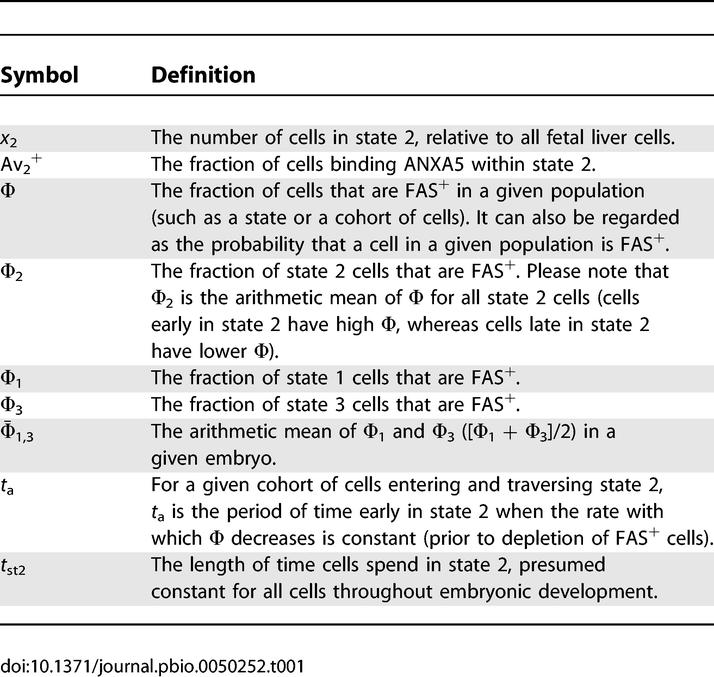
Glossary of Symbols Used in the Text

**Figure 5:**
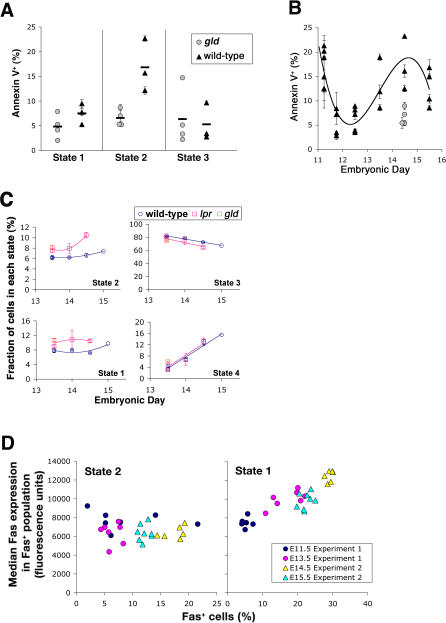
FAS Mediates Apoptosis in State 2 (A) Fraction of cells that are ANXA5^+^ in E14.5 *gld* embryos (circles) compared with strain- and age-matched wild-type embryos (triangles) in each of states 1, 2, and 3. Each data point represents the mean ± standard error of the mean for a litter of embryos; four litters containing 18 *gld* embryos were compared with three litters containing a total of 23 wild-type embryos; differences for state 2 were significant at the *p* < 0.0001 level using a two-tailed Student's *t*-test (non-equal variance). (B) State 2 ANXA5 binding data shown in (A), plotted together with the ANXA5 binding data for the entire E11–E15.5 period from Figure 4B. (C) Distribution of cells in the four fetal liver states shows an increase in state 2 cells in E14.5 and E13.5 *lpr* (magenta) and *gld* (orange) embryos, compared with strain- and age-matched wild-type embryos (blue). Each data point is the mean ± standard error of the mean of 5–12 embryos from the same litter. There is a 1.58-fold increase in *x*_2_ in *gld* and *lpr* embryos at E14.5 (*p* < 0.0001) and a 1.29-fold increase at E13.5 (*p* < 0.0001). (D) Median FAS expression per cell in the FAS^+^ cell population in states 1 and 2. Each point represents data from an individual embryo. Two experiments are shown; in each, two litters of different embryonic ages were examined simultaneously. No normalization was performed on the fluorescence data.

We assessed the role of FAS further, by measuring ANXA5 binding in embryos that carry an inactivating mutation in either FAS *(lpr)* or FASL *(gld)*. Measurements were made at E14.5, when *x*_2_ and Av_2_^+^ are relatively unaffected by small variations in the true age of embryos. We found a 2.5-fold decrease in Av_2_^+^ in *gld* embryos (from 17% to 7%, *p* < 0.001; Figure 5A and 5B). There was little or no change in ANXA5 binding in states 1 and 3. We also found a 1.58-fold increase in the relative number of state 2 cells, *x*_2_, in *gld* and *lpr* embryos at E14.5 (*p* < 0.0001) and a 1.29-fold increase at E13.5 (*p* < 0.0001), compared with matched wild-type embryos of the same age (Figure 5C). There was little change in the proportions of cells in other states. These data confirm that FAS-mediated apoptosis occurs principally in state 2, where it accounts for a substantial portion of ANXA5 binding.

Since ANXA5 binding in state 2 is largely due to FAS-mediated apoptosis, the trough in Av_2_^+^ at E12.5 is likely to be due to the trough in Φ_2_ at this time (Figure 4A and 4B). We sought to distinguish two potential mechanisms that might account for the trough in Φ_2_. FAS^+^ cells may be lost as a result of FAS-mediated apoptosis. Alternatively, a signaling event may suppress FAS expression in state 2 cells. In the latter case, the decrease in the number of FAS^+^ cells might be expected to be associated with a decline in the mean level of FAS expression per cell. However, we found that FAS expression per cell within the FAS^+^ population remained constant in spite of large variations in the size of this population (Figure 5D). Therefore, the trough in state 2 FAS^+^ cells at E12.5 is likely to be due to their loss through apoptosis, consistent with results obtained above that suggest the presence of FAS-mediated apoptosis in state 2.

### FAS-Mediated Apoptosis in State 2 Is Autoregulatory, Since Its Rate Is Proportional to *x*_2_^2^

Apoptosis of FAS^+^ cells in state 2 could, in principle, be triggered by any FASL^+^ cells within fetal liver, whether erythroid cells in any of states 1 to 4, or non-erythroid cells that form up to 10% of state 1. To identify the source of FASL^+^ cells responsible for triggering FAS-mediated apoptosis in state 2, we examined the rate of this process. If FAS-mediated apoptosis is due to an ff23-type, autoregulatory interaction, where both FAS^+^ and FASL^+^ cells are within state 2, its rate should be proportional to *x*_2_^2^ (see equation 2 above, and table in Protocol S1 detailing the rate dependency for each feedback/feedforward interaction). Specifically, the interaction between state 2 FAS^+^ and FASL^+^ cells should, by mass action, be proportional to the product of their respective numbers, or ([FAS^+^] × *x*_2_) × ([FASL^+^] × *x*_2_) = [FAS^+^] × [FASL^+^] × *x*_2_^2^, where [FAS^+^] and [FASL^+^] are the proportions of state 2 cells expressing FAS and FASL, respectively.

Our data show that, between E11.5 and E15.5, *x*_2_ varies over a 5-fold range (Figure 4A). Remarkably, we found that throughout this period, Φ_2_ is inversely proportional to *x*_2_ (Figure 6A). Further, Av_2_^+^ is also an inverse function of *x*_2_ throughout most of development (Figure 6B), except at E11.5 (Figure 6C), where Av_2_^+^ is higher, for a given *x*_2_, than later in development. This effect at E11.5 may be due to a slower clearance of apoptotic cells in very early fetal livers.

The analysis below shows that the inverse relationship between Φ_2_ and *x*_2_ is a consequence of a FAS-mediated cell loss whose rate is proportional to *x*_2_^2^, strongly supporting an autoregulatory interaction between state 2 cells as the cause of their apoptosis.

To assess the rate of FAS^+^ cell loss, we considered how the proportion of FAS^+^ cells, Φ, decreases within a given cohort of cells that have entered state 2 together and are advancing through the state. (Erythroblasts are presumed to enter state 2 from state 1 continuously. We define a “cohort” as all erythroblasts that enter state 2 together at a given point in time.) The first such cohort of differentiating erythroblasts enters state 2 at ≈E11.5. The wide range of *x*_2_ values found in E11.5 embryos (Figure 6A and 6D) reflects the different extents to which this first cohort of cells has progressed within state 2 in different embryos; in embryos with higher *x*_2_, the first cohort of cells has advanced further in state 2 than in embryos where *x*_2_ is low (Protocol S2, section 2.4D). Therefore, the inverse relationship that we observe between *x*_2_ and Φ_2_ in E11.5 embryos (Figure 6A and 6D) suggests that FAS^+^ cells are lost rapidly as this first cohort of erythroblasts advances through the state (Figure 6D and 6H). The longer this first cohort of erythroblasts has spent in state 2, the lower the proportion of FAS^+^ cells (Φ) within the cohort. In the ensuing E12.5–E15.5 period, it is likely that a similar process continues for each cohort of cells entering and traversing state 2. For each such cohort, Φ is highest at the time cells enter state 2, and decreases as the cells traverse state 2. Since there is little FAS-mediated apoptosis in states 1 and 3, we used the measured fractions of FAS^+^ cells in state 1 (Φ_1_) and state 3 (Φ_3_) as estimates of Φ when cells enter (*t =* 0) and exit (*t = t*_st2_) state 2, respectively (Figure 6E).

Figure 6E illustrates how Φ might decrease as a function of time within a cohort of cells traversing state 2. (This may also be regarded as equivalent to the distribution of Φ for all state 2 cells at a given embryonic age, with the *x*-axis denoting the length of time, *t,* that a particular cell has spent in state 2, and the *y*-axis denoting the probability, Φ, that such a cell is FAS^+^). To assess the rate with which Φ decreases, we compared Φ_2_, the arithmetic mean of Φ for all cells in state 2, with Φ_1_ and Φ_3_ *.* If Φ decreases at a constant rate throughout the time cells spend in state 2 (dashed red line, Figure 6E), Φ_2_ should equal the average of Φ_1_ and Φ_3_ (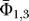 , where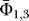 *=* [Φ_1_ *+*Φ_3_]/2). If, however, an initial rapid loss leads to depletion of FAS^+^ cells before state 2 is complete (solid red line, Figure 6E), 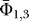 would be higher than Φ_2_. We found that the ratio of 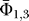 to Φ_2_ alters with embryonic age in a manner similar to *x*_2_ (Figure 6F; Protocol S2, section 2.5A and 2.5B). In embryos younger than E14.5, the ratio 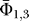 /Φ_2_ is larger than one, suggesting that, for a given erythroblast cohort traversing state 2, an initial rapid decline in Φ slows down as FAS^+^ cells are depleted (Figure 6F and 6H).

We assumed that prior to FAS^+^ cell depletion, Φ decreases at an approximately constant rate, for a period of length *t*_a_. The area under the curve described by Φ (shaded red, Figure 6G, panel i) approximates the area of a triangle of base *t*_a_ (shaded blue, Figure 6G, panel ii); this area is given by 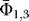 *t*_a_ (Figure 6G, panel ii). Since Φ_2_ is the mean value for Φ throughout state 2, it is equivalent to the height of a rectangle (black dashed lines, Figure 6G, panel ii) whose area is equal to that of the blue triangle, and whose base is the entire length of state 2, *t*_st2_. The equivalence in the areas of the blue triangle and black rectangle in Figure 6G, panel ii, gives the result that *t*_a_ *,* the length of the period when FAS^+^ cell loss is constant, is directly proportional to Φ_2_:

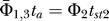
or

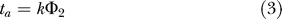
where *k* = *t*_st2_/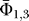 (Φ_1_, Φ_3_, and *t*_st2_ are relatively constant). Equation 3 also shows the ratio 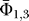 /Φ_2_ to be equivalent to *t*_st2_/*t*_a_. From this and from Figure 6F, at E12.5, *t*_a_ is one-half to one-third of *t*_st2_, increasing with embryonic age (see Figure 6H).

The direct proportionality between *t*_a_ and Φ_2_ (equation 3) makes it possible to deduce that *t*_a_ is also inversely proportional to *x*_2_ (equation 5 below), since we found experimentally (Figure 6A) that Φ_2_ is inversely proportional to *x*_2_:

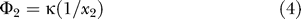
where κ is the proportionality constant in Figure 6A. Combining equations 3 and 4:

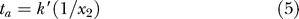
where *k′ =* κ*t*_st2_/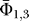
_._

The rate of decline in Φ during the period *t*_a_ is represented by the slope of the blue triangle's hypotenuse in Figure 6G, panel ii, and 6H, or (Φ_1_ *−* Φ_3_)/*t*_a_. The rate of loss of FAS^+^ cells, *dC*/*dt,* is given by the rate of decline in Φ, multiplied by *x*_2_ (since Φ represents the fraction of cells that are FAS^+^ during state 2):

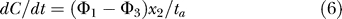



Since *t*_a_ is inversely proportional to *x*_2_ (equation 5), it is possible to re-write equation 6, expressing *dC*/*dt* as a function of *x*_2_ , and giving the result that the rate of loss of FAS^+^ cells from state 2 is proportional to *x*_2_^2^:

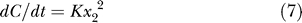
where *K =* (Φ_1_ *−* Φ_3_) 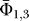 /κ*t*_st2_.

Of note, the proportionality constant, relating the rate of FAS^+^ cell loss to *x*_2_^2^, contains the term Φ_1_^2^ (equation 7). This term represents the product of the available fractions of FAS^+^ and FASL^+^ cells when cells enter state 2, since FAS and FASL expression are linearly related (Figure 4F). Thus, the rate of loss of FAS^+^ cells in state 2 is found experimentally to be proportional to [FAS^+^] × [FASL^+^] × *x*_2_^2^, as predicted for an autoregulatory ff23.

The inverse relationship we also found between Av_2_^+^ and *x*_2_ can be explained if, as is likely, ANXA5^+^ cells are cleared fast compared with the length of state 2. If so, as cells traverse state 2, the proportion of ANXA5^+^ cells will closely reflect Φ, and their mean, Av_2_^+^_,_ will relate to *x*_2_ in the same way as Φ_2_. The inverse relationship between Av_2_^+^ and *x*_2_ therefore provides an independent assay confirming that the rate of loss of FAS^+^ cells is inversely related to *x*_2_^2^.

### The FAS-Mediated Autoregulatory Interaction Increases the Robustness of Erythropoiesis

The ff23 interaction was selected based on criteria that included robustness of the erythropoietic network to perturbations in parameter values. It therefore follows that the absence of ff23 should result in increased sensitivity to such perturbations. The *lpr* and *gld* mice we examined under laboratory conditions are housed in a stable, controlled environment, and are genetically identical, inbred mouse strains. They are therefore subjected to relatively few external perturbations. Nevertheless, we found that there is higher variability between individual *lpr* or *gld* embryos at a given embryonic age than in otherwise genetically identical wild-type embryos of the same age (Table 2).

**Table 2:**
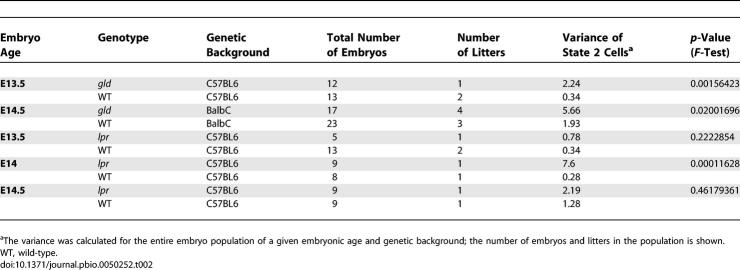
Increased Variance in the Number of State 2 Cells in Embryos Mutant for FAS or FASL

Specifically, we examined the dataset of *lpr* and *gld* embryos presented in Figure 5 (embryos were on C57BL6 background in Figure 5C, and on BalbC background in Figure 5A). We compared the variance in the number of cells in state 2 (*x*_2_), where the ff23 interaction is exerted, for all embryos at a given embryonic age and on a given genetic background. Strikingly, the variance between *lpr* or *gld* embryos was consistently higher than in wild-type controls, reaching statistical significance (using an *F*-test) in three of the five comparisons (Table I). Therefore, the inherent noisiness of the developmental process was sufficient to perturb the number of cells in state 2, and this noisiness was mitigated by the regulatory ff23 interaction mediated by FAS/FASL. It is plausible to suggest that state 2 cells in *lpr* and *gld* embryos might deviate even more significantly from their programmed developmental trajectory in response to severe perturbations under nonlaboratory conditions.

These findings provide an additional and more direct a posteriori justification for using robustness as one of the criteria for identification of new interactions in developmental networks.

## Discussion

The work we present here aims to tackle a key challenge. In many biological systems, multiple candidate regulators have been identified by bioinformatics and other methods. Given the complexity of biological networks, how can their principal regulatory features be identified? We were particularly interested in identifying interactions that endow a network with robustness to external and internal perturbations (represented by alterations of parameter values in the corresponding dynamical model), a property that is encountered frequently in developmental networks. We approached this problem by hypothesizing that, although a large number of interactions might be present in any given system, only a small subset is responsible for its robust regulatory behavior. This hypothesis guided us in developing a novel computational algorithm that identified a key negative autoregulatory interaction that controls the rapid growth phase of erythropoietic tissue in the developing embryo. We show that cells at an early erythroid differentiation stage, here termed state 2, undergo FAS-mediated apoptosis as a result of an intercellular interaction between FAS^+^ and FASL^+^ cells in the same state. Further, we find that apoptosis proceeds at a rate that is proportional to the square of the number of cells in state 2, a property that would buffer fluctuations in erythropoietic tissue growth and ensure it progresses close to its preprogrammed developmental trajectory.

The combined biological and computational approach we applied here could be used to identify homeostatic interactions regulating other tissues with rapid growth or turnover, such as skin, intestinal epithelium, or tumor metastasis.

### Novel Features of the Computational Algorithm

An intrinsic and unique aspect of our developmental network reconstruction approach is that it explicitly selects for interactions that endow the developmental system with certain key regulatory properties. First, we explicitly assume that the underlying developmental process is robust to perturbations that might arise as a result of disease or fluctuations in gene dose, temperature, or nutrients. Robustness of developmental processes, though widely assumed and confirmed in multiple studies [48], including several combining modeling and experiment [1,49–52], has not been explicitly used for developmental network reconstruction. Here we tested the performance of each interaction in the context of many potential network topologies; we then developed a metric that ranks the extent to which each interaction endows the networks in which it participates with resistance to external perturbations of network parameters (the “robustness” criterion; Figure 3B). Second, by using a differential equations description of the system, rather than a probabilistic approach, we were able to evaluate the rates of different processes. This allowed us to develop a metric that selects for interactions whose strengths are relatively unaffected by the precise topology of the rest of the network (the “consistency” criterion; Figure 3C). This has clear biological relevance: given genetic variability within a population, the precise network topology may differ in different individuals. However, interaction(s) ranked high by the consistency metric might be relatively unaffected by this type of variation and be present with comparable strength in the network of all individuals. Two other metrics (“fitness” and “control”; Figure 3A and 3D) selected for interactions that scored high on regulating how well the networks in which they participate fit the experimental dataset, and on the extent to which they control and maintain this fitness in the face of perturbations.

Requiring that putative interactions satisfy all of the above criteria can strongly constrain the number of these interactions. Indeed, we have found that only two interactions were consistently ranked high, with only one of them retaining the high ranking during additional perturbations of the original experimental dataset or the details of the computational analysis. The benefit of this highly selective ranking is that a relatively high confidence can be placed in the interactions chosen. On the other hand, by relaxing some of the criteria, one can expand the list, progressively testing for lower ranking interactions. In this process, different weights could be placed on different criteria. For example, by relaxing the fitness criterion, one can predict the potential importance of the fb43 interaction. The particular set of interactions identified is likely to be strongly dependent on the nature of the developmental process and, to a smaller degree, the particular dataset used for network reconstruction.

An important feature of the algorithm is that it deals with the relative number of cells in each of the developmental states, without explicitly breaking the rate of change in cell number down into its components, namely, the rates of cell death, cell division, and cell differentiation. Consequently, when the pivotal ff23 interaction was identified, the algorithm did not have the power to specifically predict which of these three processes is responsible for the interaction. The molecular candidates for mediating the ff23 interaction had to be identified subsequently, through reasonable assumptions about the system's biology. The level of abstraction at which we chose to develop the models underlying the algorithm was a result of the type of biological measurements that can be made reliably and reproducibly in erythropoietic and other developmental networks. There is no simple way to measure the rates of cell death, cell division, or cell differentiation in tissue in vivo at the present time. By contrast, the flow-cytometric datasets that we used could reliably and rapidly document the rate of change of the relative number of cells in each state during development. In the future, technological advances and increased knowledge of the erythropoietic system may allow the reconstruction of a more detailed mechanistic model. It is noteworthy, however, that in spite of these limitations, the algorithm we present had the power to provide a strong and precise prediction that successfully guided our subsequent biological investigation. We therefore suggest that dynamic measurements of cell frequency, which are relatively easy to obtain, may serve as the basis of predictive computational modeling in many other tissues.

### The Role of FAS-Mediated Apoptosis in Erythropoietic Tissue Growth

Between E11.5 and E15.5, fetal liver mass increases over 100-fold, a rate ten times faster than overall embryonic growth in the same period (Protocol S2, section 2.1). Small deviations from the optimal amplitude and duration of this burst in fetal liver growth could be catastrophic. The autoregulatory interaction we identified between FAS^+^ and FASL^+^ cells within state 2 leads to apoptosis of state 2 FAS^+^ cells at a rate that is proportional to the square of the relative number of cells within that state (*x*_2_^2^). Therefore, FAS^+^ cells within state 2 constitute a reserve progenitor pool that can be rapidly tapped whenever *x*_2_ is inappropriately low. Conversely, an inappropriately fast growth in the number of state 2 cells, or *x*_2_, would be damped rapidly by a sharp increase in apoptosis.

It was previously thought that, unlike adults, embryos do not possess an erythropoietic reserve, because of their higher vulnerability to mutations that lower erythropoietic rate [53–55]. This vulnerability can still be accounted for by the much smaller erythropoietic reserve in the embryo compared with the adult. Indeed, the principal function of the adult erythropoietic reserve is to increase erythropoietic rate in response to tissue hypoxia. By contrast, our study suggests that the role of fetal reserve is to stabilize the relative number of erythroid progenitors in state 2 and hence erythropoietic rate, so that it adheres to the developmentally programmed rate. We recently found that adult erythroblasts also express FAS and FASL, raising the possibility that an ff23-type interaction may help maintain adult baseline erythropoietic rate. Interestingly, FAS expression in adult erythroblasts is down-regulated during erythropoietic stress [46].

The erythroblastic island architecture (Protocol S2, section 2.2) maintains close apposition of erythroblasts and is likely to be critical in facilitating the FAS-mediated apoptosis between state 2 cells. Simple mixing of cells from different states in vitro, in the absence of the island structure, does not reproduce the dependence we observed in vivo of FAS-mediated apoptosis on *x*_2_ (Protocol S2, section 2.6).

### A Novel Role for FAS-Mediated Apoptosis in Fetal Development

To date, apoptosis had been implicated in the development of a unique set of tissues, where tissue function depends on selection—from amongst all available progenitors—of cells with specific characteristics, such as optimal cell connectivity in neural tissue, specific antigen receptors in lymphocytes, or the sculpturing of tissue architecture [56–58]. Here we show that apoptosis may also participate in development solely as a homeostatic regulator. Surviving erythroblasts presumably only differ from those that undergo apoptosis in that they lack FAS. How the expression of FAS (and FASL) is diversified within the progenitor populations merits additional investigation.

FAS and FASL have clear homeostatic functions in immune cells in the adult [59]. Their pattern of expression had suggested non-immune, developmental functions [47] in the central nervous system [60–62] and fetal lung [63,64], but no deficits in these organs in *lpr* and *gld* mice were found. The role we identify here for FAS/FASL in fetal erythropoietic development raises the possibility of homeostatic developmental functions for these molecules in other tissues.

## Materials and Methods

### Implementation of the algorithm.

The details of the algorithm development and implementation are given in Protocol S1.

### Mice.

C57BL6j and BalbCj *lpr* (B6.MRL-*Tnfrsf6lpr*/J) and *gld* (B6Smn.C3-*Tnfsf6gld*/J) mice were purchased from the Jackson Laboratory (http://www.jax.org/). Timed pregnancies were set up and embryos analyzed on indicated days. Fetal liver cells were processed on ice or at 4 °C throughout the procedures outlined.

### Determination of embryonic age.

Wild-type embryos were each allotted an embryonic age equal to that calculated from the timing of gestation adjusted ±0.5 d. The addition or subtraction of 0.5 d was done based on morphological appearance of the embryo and on the relative developmental status of the erythropoietic system, judged from the CD71/Ter119 histograms. In the case of comparisons between *lpr, gld,* and wild-type embryos, embryonic age was determined by the timing of gestation, and adjusted ±0.5 d based on variations in embryo morphology as well as embryo weight. The comparisons between proportions of cells in each state in *lpr, gld,* and wild-type embryos were carried out on E13.5 and E14.5, a time when the relative proportions of cells in states 1 and 2 in wild-type embryos vary little with embryonic age (Figure 2).

### Antibody staining and flow cytometry.

Freshly isolated fetal livers were mechanically dissociated and strained through a 70-μm strainer in the presence of phosphate-buffered saline and 5% fetal calf serum. Cells were immunostained at 4 °C in phosphate-buffered saline and 5% fetal calf serum in the presence of rabbit IgG (200 μg/ml, Jackson ImmunoResearch Laboratories, http://www.jacksonimmuno.com/) to block Fc receptors. Cells were incubated with 1 μg/ml PE-conjugated anti-Ter119 (BD Biosciences, http://www.bdbiosciences.com/) and 1 μg/ml biotin-conjugated anti-CD71 (BD Biosciences) antibodies for 20 min, followed by 10 min of incubation with allophycocyanin-conjugated streptavidin (Molecular Probes,http://probes.invitrogen.com). Cells were also stained with either 7AAD (Viaprobe, BD Biosciences) or DAPI (Roche, http://www.roche.com/) in order to exclude dead cells from analysis. In multi-parameter analysis that included staining for FAS or FASL, cells were treated similarly but stained simultaneously for 1 h with PE-conjugated anti-Ter119, FITC-conjugated anti-CD71 (BD Biosciences), 5 μg/ml biotin-conjugated anti-FAS (JO2 clone, BD Biosciences), or 5 μg/ml biotin-conjugated anti-FASL (MFL3 clone, BD Biosciences). This was followed by incubation with allophycocyanin-conjugated streptavidin and DAPI or 7AAD, as above. The same antibody specificities were also used with different fluorochrome combinations, yielding very similar results. Cells were analyzed for four-color fluorescence using either a FACSCalibur (BD Biosciences) or an LSRII (BD Biosciences) flow cytometer. Data were analyzed using FlowJo software (TreeStar, http://www.treestar.com/). ANXA5 staining was carried out according to manufacturer's instructions (BD Biosciences).

### Cell sorting.

Cell sorting of fetal liver cells was carried out on a DakoCytomation MoFlo (Dako, http://www.dako.com/).

### In vitro mixing experiments.

Cells from freshly isolated fetal livers were kept at 4 °C and stained for TFRC and LY76. Cells from each of the states 1 to 4 were sorted. Cells from the indicated states were mixed at the indicated proportions, and plated in 96-well format in IMDM, in the presence of 4 μl/500 ml of 2-mercaptoethanol, 20% fetal calf serum, and 0.05 U/ml EPO (Amgen, http://www.amgen.com/). Cells were incubated at 37 °C for 0, 2.5, or 5 h. Cells were then placed at 4 °C and apoptosis measured using ANXA5 binding and CASP3 activation. The latter was measured using the CaspGlow kit (BioVision, http://www.biovision.com/) according to manufacturer's instructions. Analysis was carried out using the LSRII flow cytometer (BD Biosciences).

## Supporting Information

Protocol S1 Supplementary Computational Methods and Results (3.4 MB PDF)

Protocol S2 Autoregulatory FAS–FASL Interaction in Erythroid Development (3.0 MB PDF)

## Abbreviations

E[number]: embryonic day [number]
ODE: ordinary differential equation
SSR: sum of squared residuals
