# Supplementary material for: Negative Autoregulation by FAS Mediates Robust Fetal Erythropoiesis
Source: PLoS Biol. 2007 Sep 25;5(10):e252. doi: 10.1371/journal.pbio.0050252 (PMC1988857; doi:10.1371/journal.pbio.0050252)
Supplement: Supplementary file 1 [file pbio.0050252.sd001.pdf]

Protocol S1

1.1. Definition of erythropoietic developmental states using *in vivo* Embryonic Ter119/CD71 Time Course

Developmental processes in general and erythropoiesis in particular involve a gradual acquisition by cells of key morphological features and differentiation markers. These may be used to classify developing progenitors into discrete developmental stages. Such discretization aids the structured analysis of the developmental processes. In this study, we based the discretization procedure on the expression profiles of two specific differentiation markers: Ter119, an antigen expressed on cell surfaces of more mature erythroid progenitors, and CD71, the transferrin receptor, whose expression *transiently* increases with differentiation (*1*). Generating experimental histograms of time dependent expression levels of these two surface markers measured via flow cytometry analysis, and plotting them in two dimensions (Supplementary Figs. 1-14, WinMDI v2.8 software was used for quantification of results) provides a series of snapshots of the number of cells in different states of expression of CD 71 and Ter119, as a function of developmental time. These histograms provide us with an abstract 'phase portrait' or 'phase diagram' of the system, which can potentially be used to investigate the 'trajectory' of the developing cells, dynamics of differentiation events as well as any other interesting or unusual features, e.g., relative accumulation (expressed as higher proportion) of cells in some regions of this diagram. In other words, one might find (which is indeed the case, as shown below) that developing cells rarely display certain combinations of CD71 and Ter119 levels, while other combinations are abundantly present at most of the time points investigated.

As can be seen in Supplementary Figs. 1-14, the expression levels of CD71 and Ter119 indicate that there is indeed a general 'trajectory' or 'erythropoietic wave' corresponding to differentiation events. The 'front' of this wave propagates by first moving up CD71 level, with Ter119 being relatively constant, then moving up Ter119 levels, with CD71 being relatively constant, and finally moving down CD71 levels, with Ter119 being constant. Relatively speaking, there are very few cells remaining in the wake of the wave, suggesting a very pronounced 'wave crest' populating different differentiation states. There are also identifiable regions of relative cell accumulation along this wave. For example, in all histograms, at low Ter199 levels, cells tend to fall into two separate and well localized regions of preferred CD71 expression levels (quantified more fully below). Similarly, at high Ter119 expression levels, there are preferred CD71/Ter119 expression values translated into two separable localized regions in the histograms. These regions of preferred cell 'accumulation' can help separate the developmental process into a discrete number of states, subject to a further assumption that cells can transition from state to state during differentiation. Using the CD71/Ter119 histograms, the cells in each state so defined, can be gated and used for statistical analysis. We have thus used these histograms along with the information of cell size to define 4 distinct developmental states, which might serve as convenient separation of erythropoiesis events into differentiation stages.

Specifically, we have adopted the state definition based on three lines drawn in the histograms separating the Ter119/CD71 expression plane into 4 distinct regions (see Supplementary Figs. 1-14). The first of these lines was drawn by

initially gating the cells at low Ter119 levels and then finding minima separating two peaks on the histograms of CD71 expression levels (these histograms along with the CD71 expression level histograms for all expression level of Ter119 are shown for all time points in Supplementary Figs. 1-14). By definition adopted here, this line (horizontal) separated states 1 and 2. The second line (vertical) was drawn similarly, by assaying Ter119 expression at high CD71 levels (not shown). This line separated states 2 and 3. The final line (horizontal) designed to separate states 3 and 4, was harder to identify, since no obvious separation between low and high levels of CD71 expression was seen for high Ter119 expression levels. However, two self-consistent observations indicated that there is indeed a difference between (high Ter119, high CD71) and (high Ter119, low CD71) stages. First, by looking at the data for embryonic days 13 through 14.5, one can see that there is a relative slowing or 'lingering' of the erythropoietic wave front at high CD71 and high Ter119 levels. Additionally, and more significantly, there is an identifiable separation on the erythropoietic wave, after which cells cease to decrease their size and change other characteristics (see Sup. Fig. 15), indicating mature state representing state 4 in our analysis. One can draw a line outlining the position of the front during the slow-down of the wave or another line indicating where cell size change becomes undetectable. Interestingly, these two lines are localized to almost the same region, leading to a more defined separation between (high Ter119, high CD71) and (high Ter119, low CD71) states, and thus allowing us to introduce separation between states 3 and 4 (Supplementary Fig. 15). We have thus drawn a second horizontal line separating these states. All three separation lines described above are shown in each of the panels in Supplementary Figs. 1-14. Some changes in absolute positions of the lines should be attributed to somewhat different flow cytometry settings adopted for different experiments corresponding to different time points.

The next step was to weave together a variety of data points which were taken at various stages of the erythropoietic differentiation. One should remember that the erythropoieitc wave is a result of amalgamation of data from different embryos sacrificed at different estimated periods of their developmental time. We assigned approximate times to each of these data sets, placing them in evenly spaced intervals (0.5 embryonic days), and then later tested the sensitivity of the results to this assumption (Section 4). The time course thus represented an overall dynamic profile of erythroid cell differentiation, as defined by the acquisition and loss of cell surface markers CD71 and Ter119, with correlation to cell size.

1.1.1 Embryonic Day 12.5

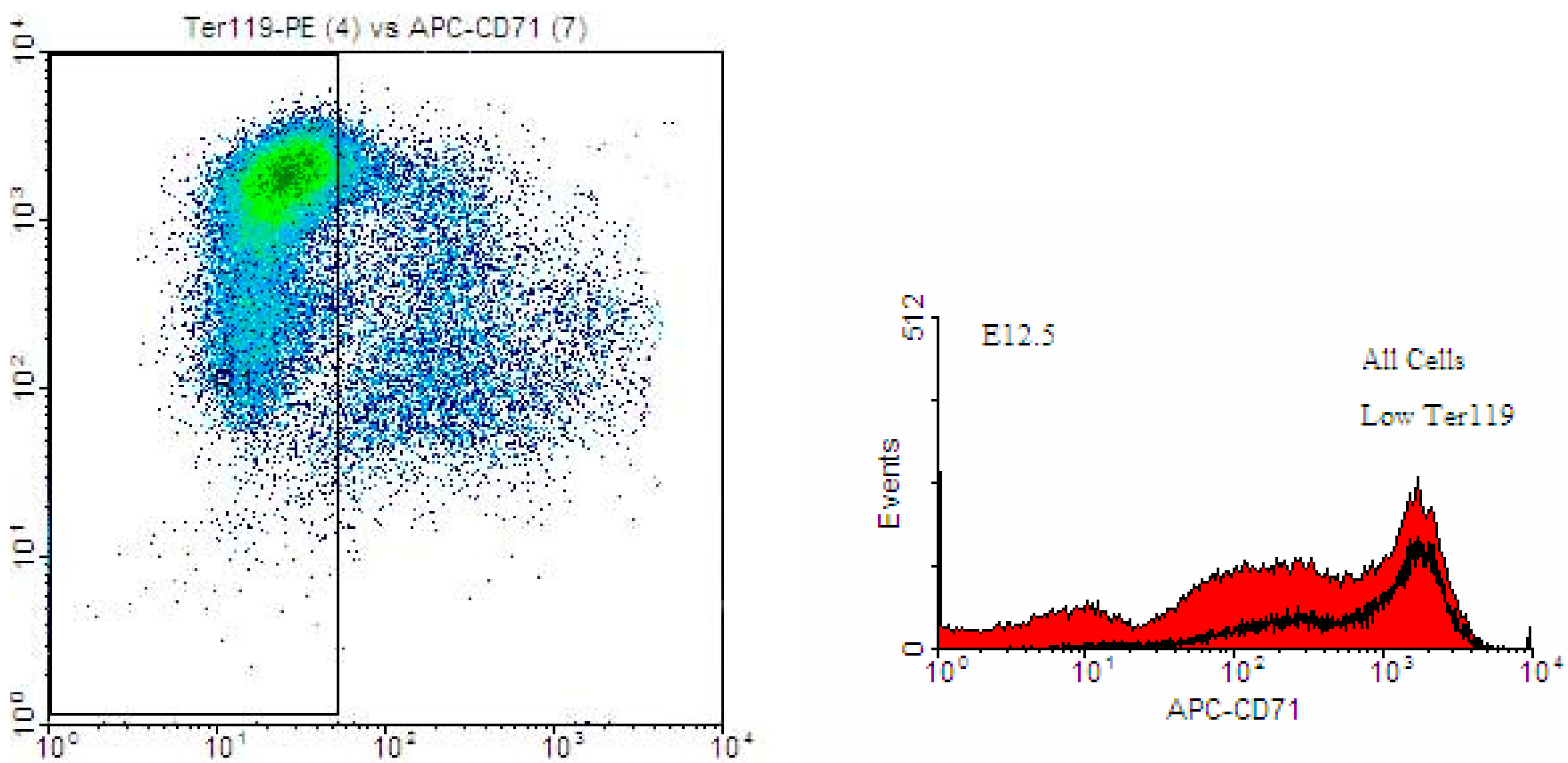

Supplementary Figure 1. Low CD71 for E12.5

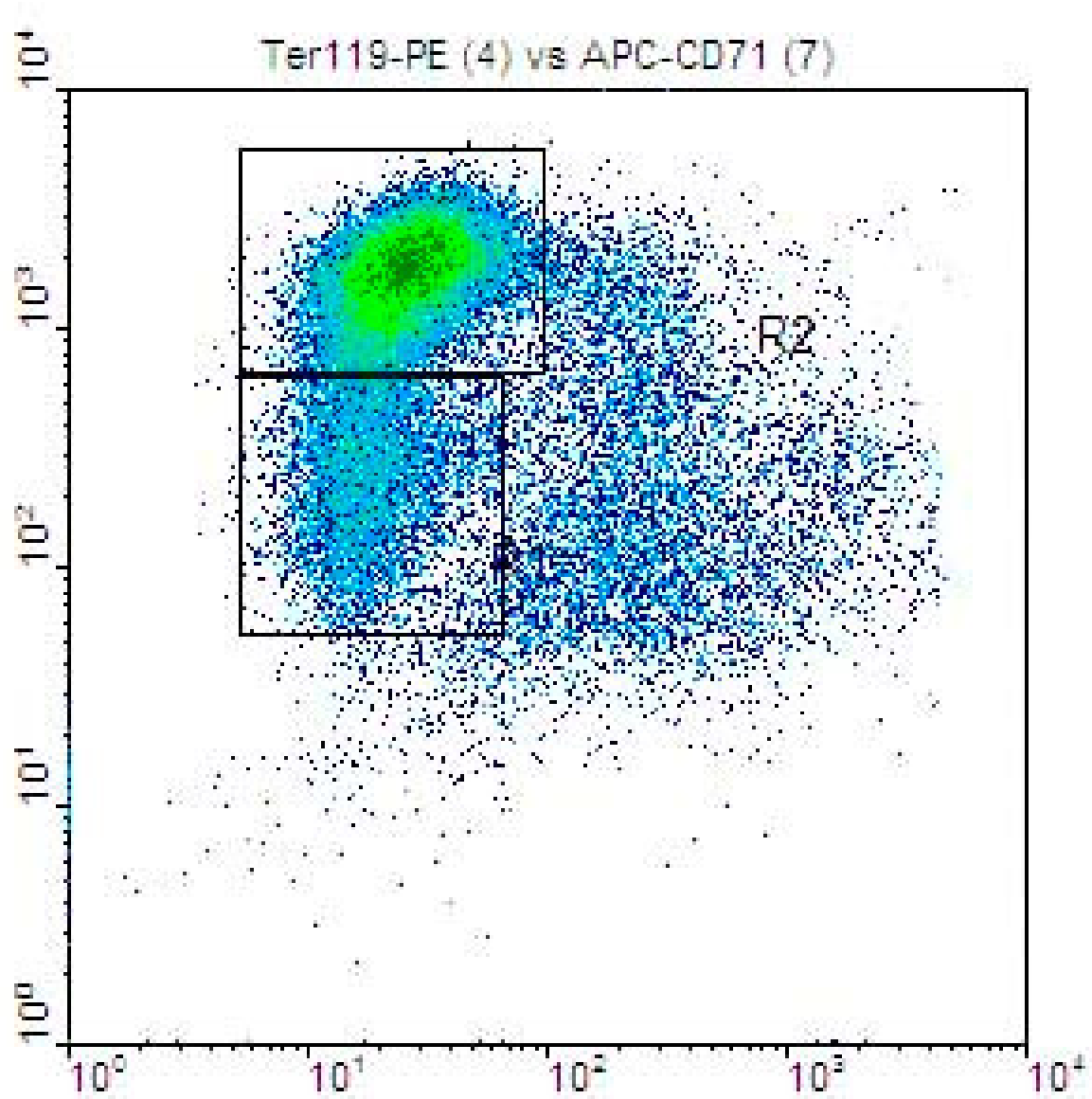

Quad	X-Mean	Y-Mean	Events	**%Gated**
(1) UL	26.7	1607.2	20424	24.51
(2) UR	0	0	0	**0**
(3) LL	19.4	209.6	10757	12.91
(4) LR	0	0	0	**0**

Supplementary Figure 2. Gating Statistics for E12.5

1.1.2 Embryonic Day 13.0

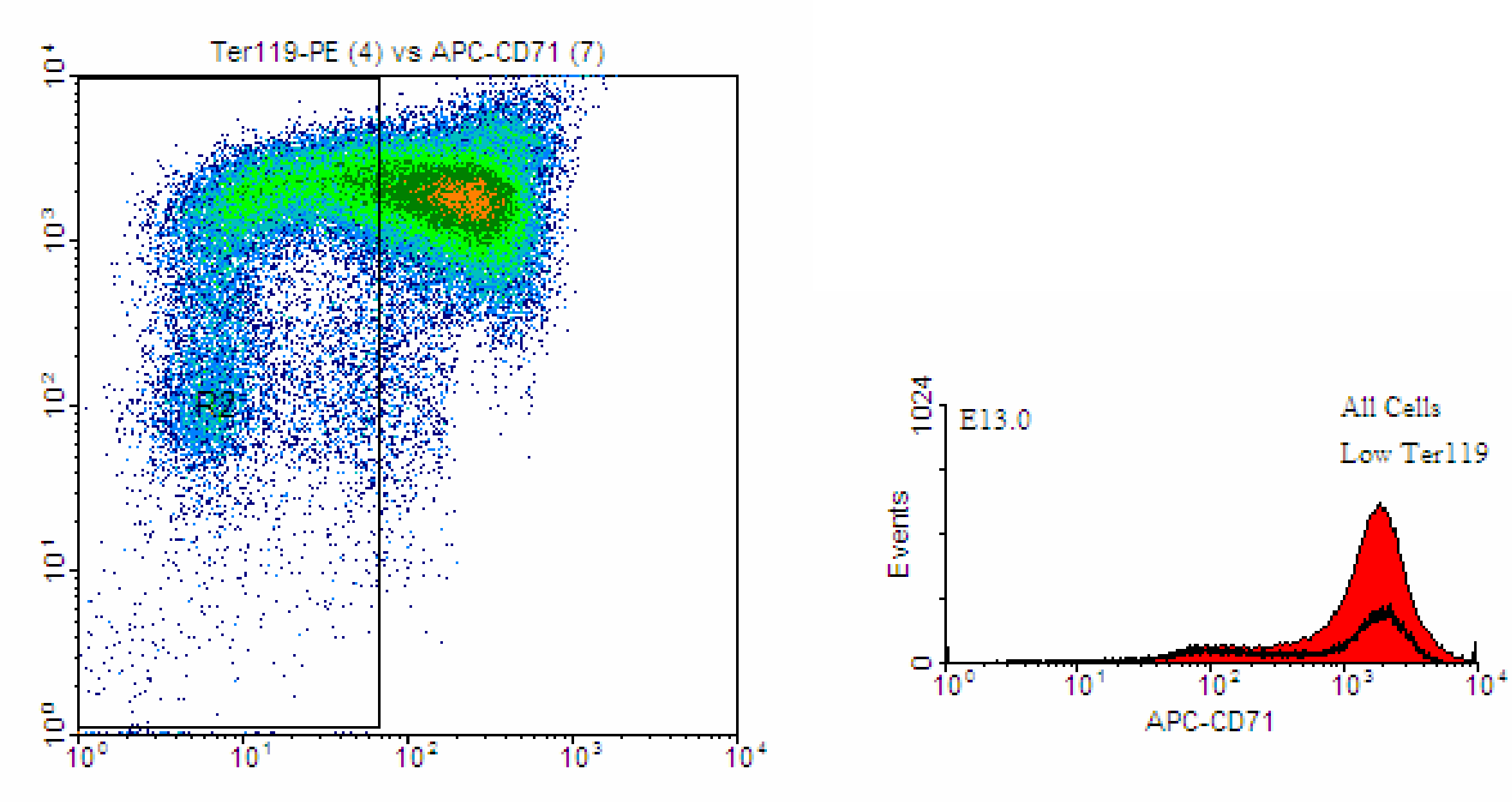

Supplementary Figure 3. Low CD71 for E13.0

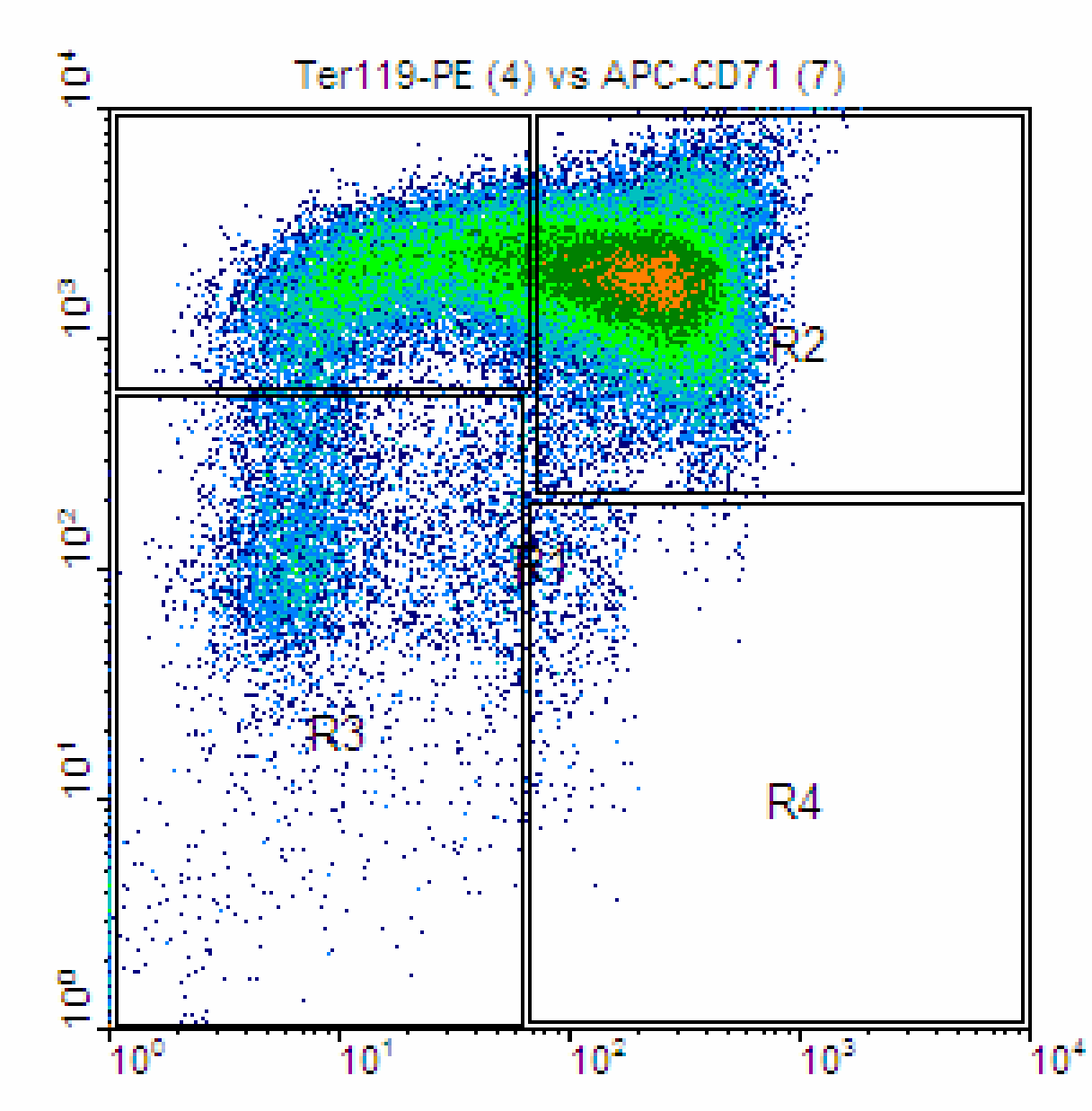

Quad	X-Mean	Y-Mean	Events	**%Gated**
(1) UL	20	1804.1	25717	**25.72**
(2) UR	218.7	1650.8	54191	**54.19**
(3) LL	9.4	79.1	15972	**15.97**
(4) LR	129.4	58.1	2442	**2.44**

Supplementary Figure 4. Gating Statistics for E13.0

1.1.3 Embryonic Day 13.5

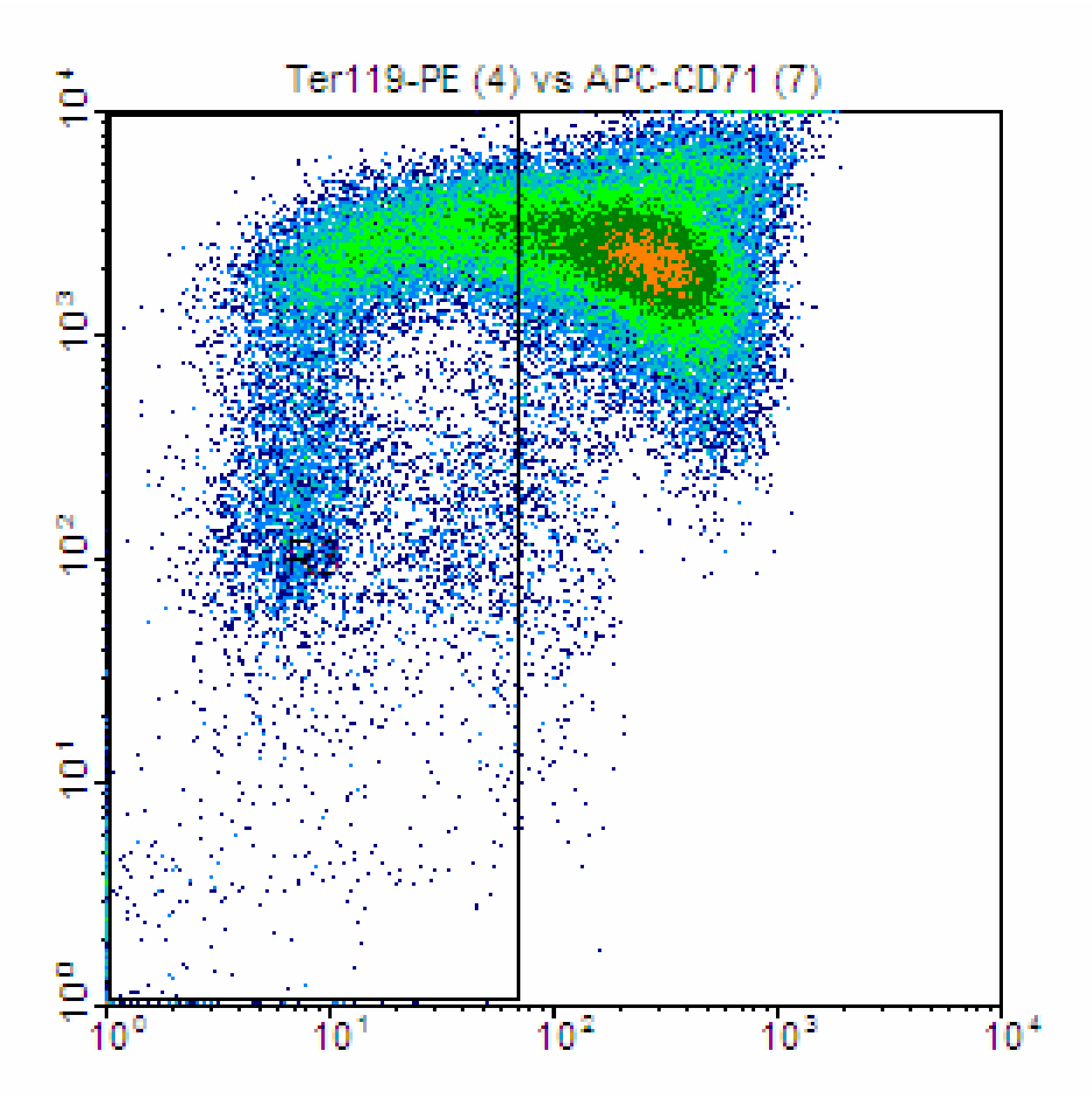

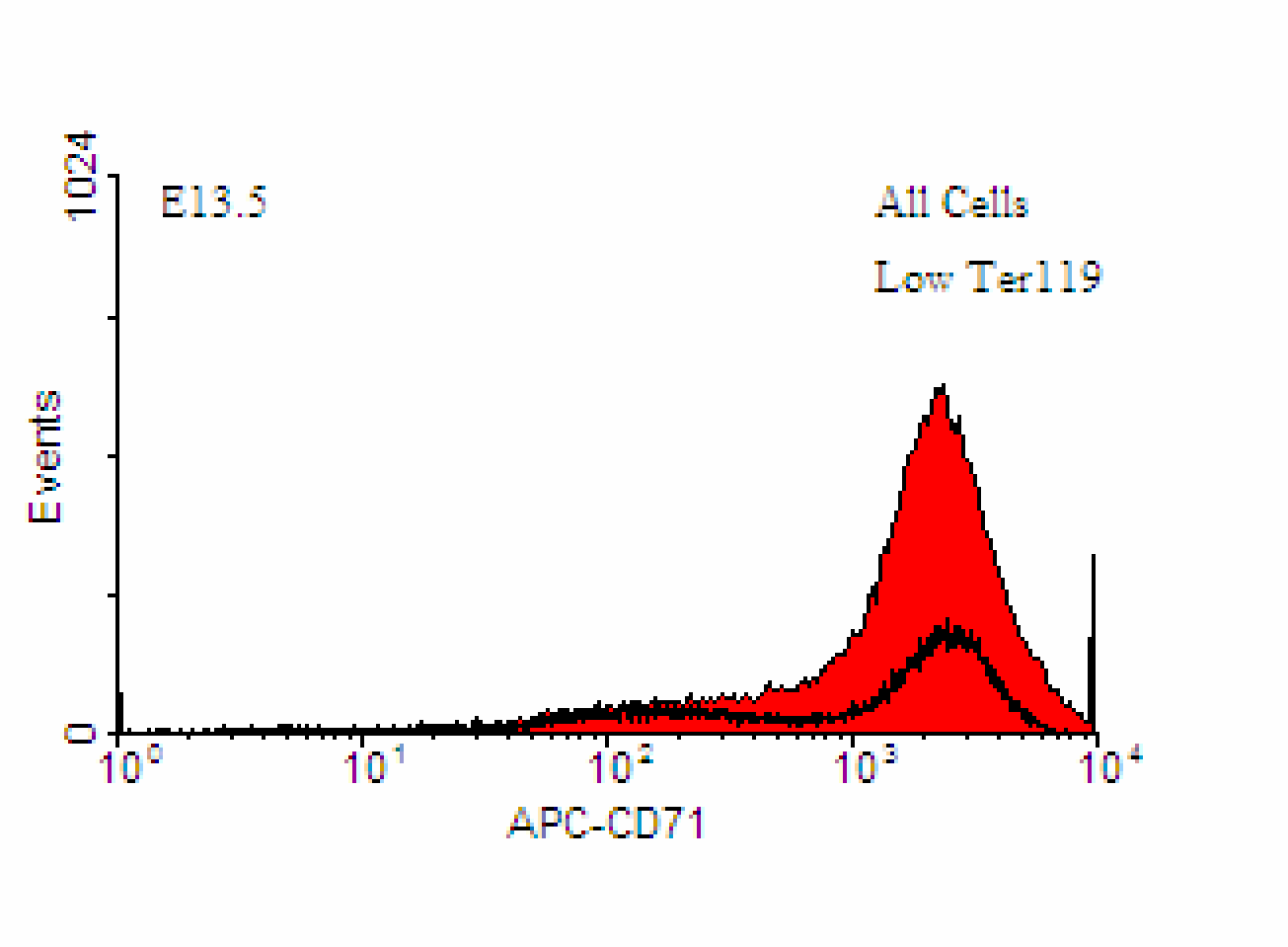

Supplementary Figure 5. Low CD71 for E13.5

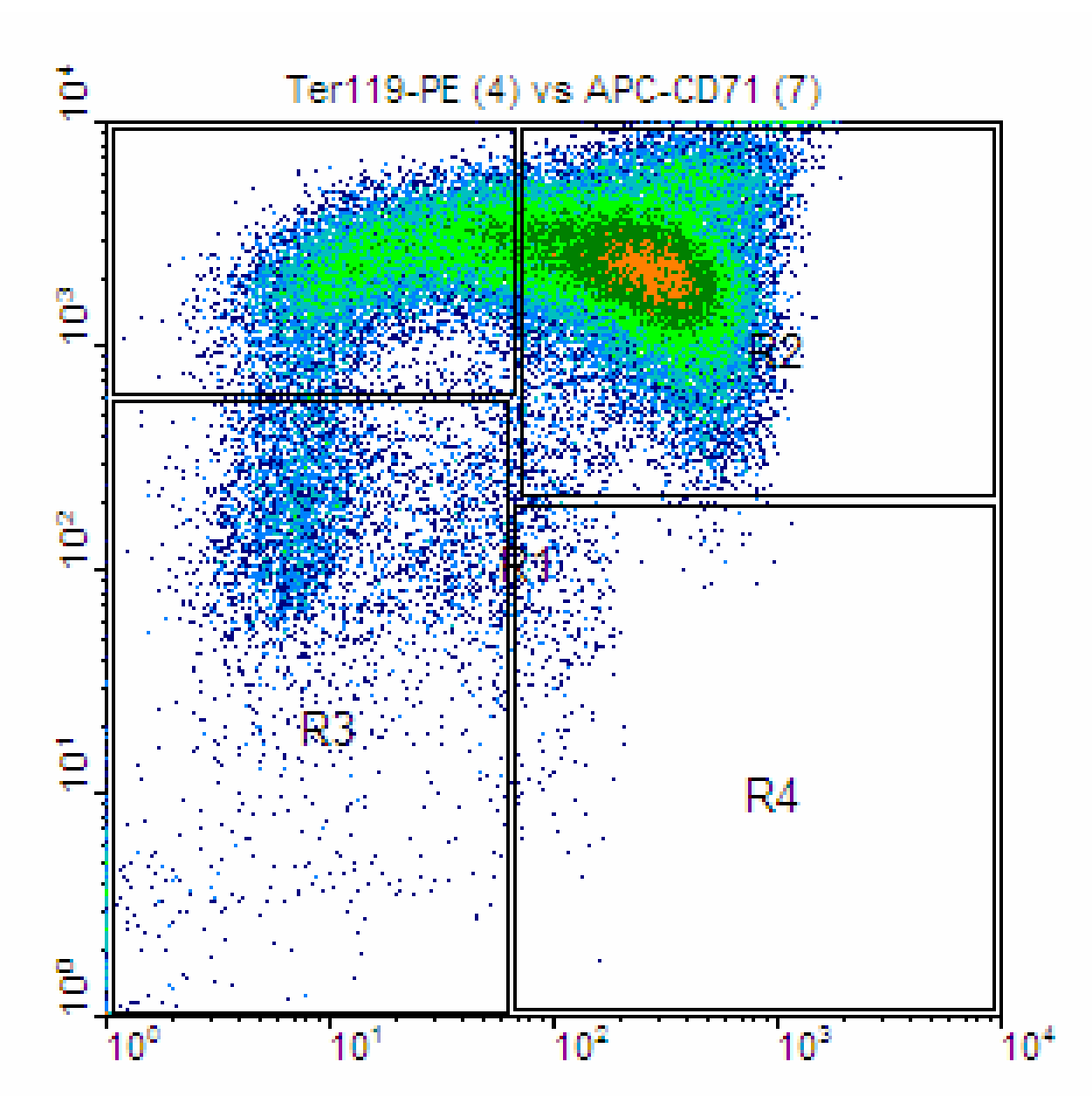

Quad	X-Mean	Y-Mean	Events	%Gated
(1) UL	20	2217.3	23173	**23.17**
(2) UR	264.8	2049.6	59840	**59.84**
(3) LL	9.9	79.0	13180	**13.18**
(4) LR	138.5	55.5	1981	**1.98**

Supplementary Figure 6. Gating Statistics for E13.5

1.1.4 Embryonic Day 14.0

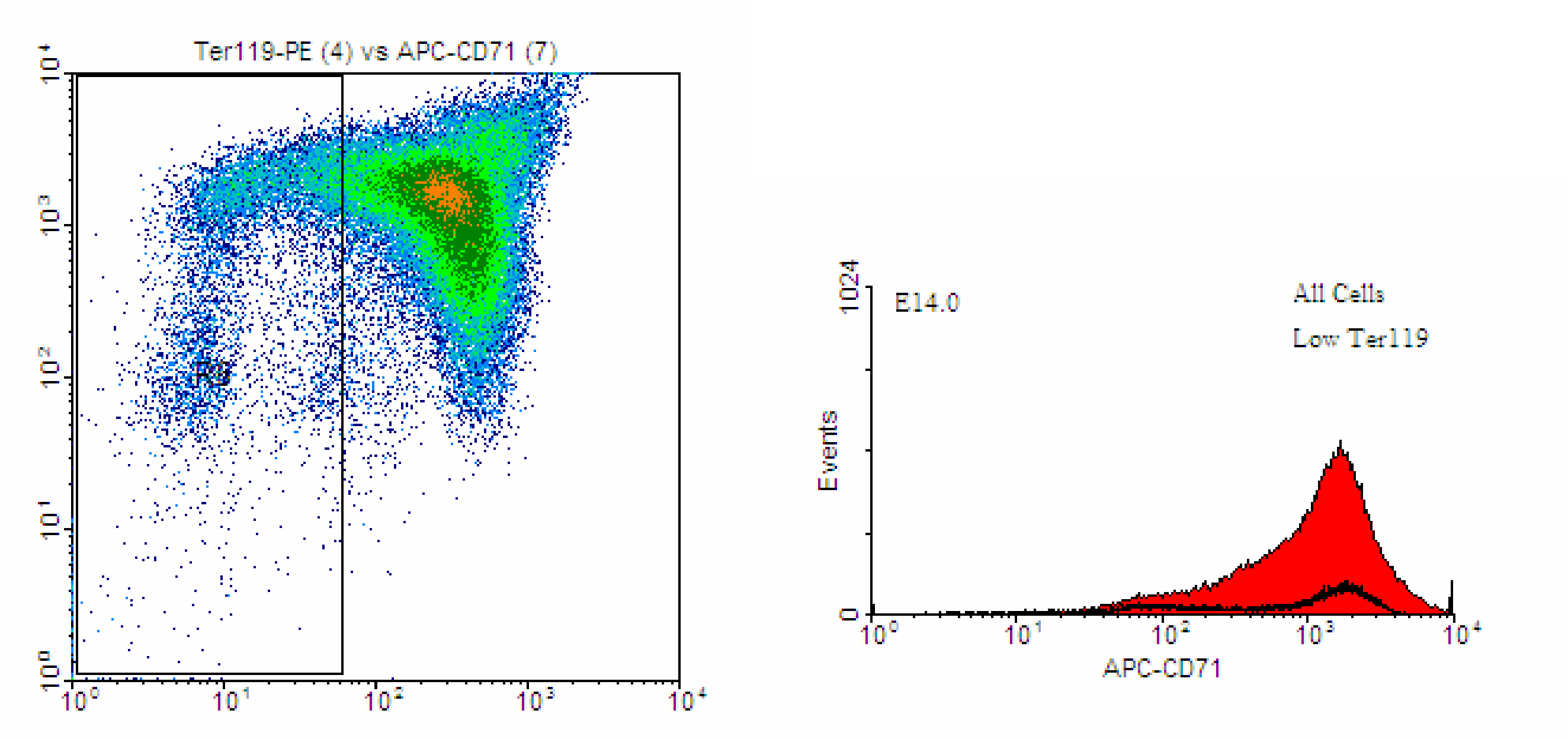

Supplementary Figure 7. Low CD71 for E14.0

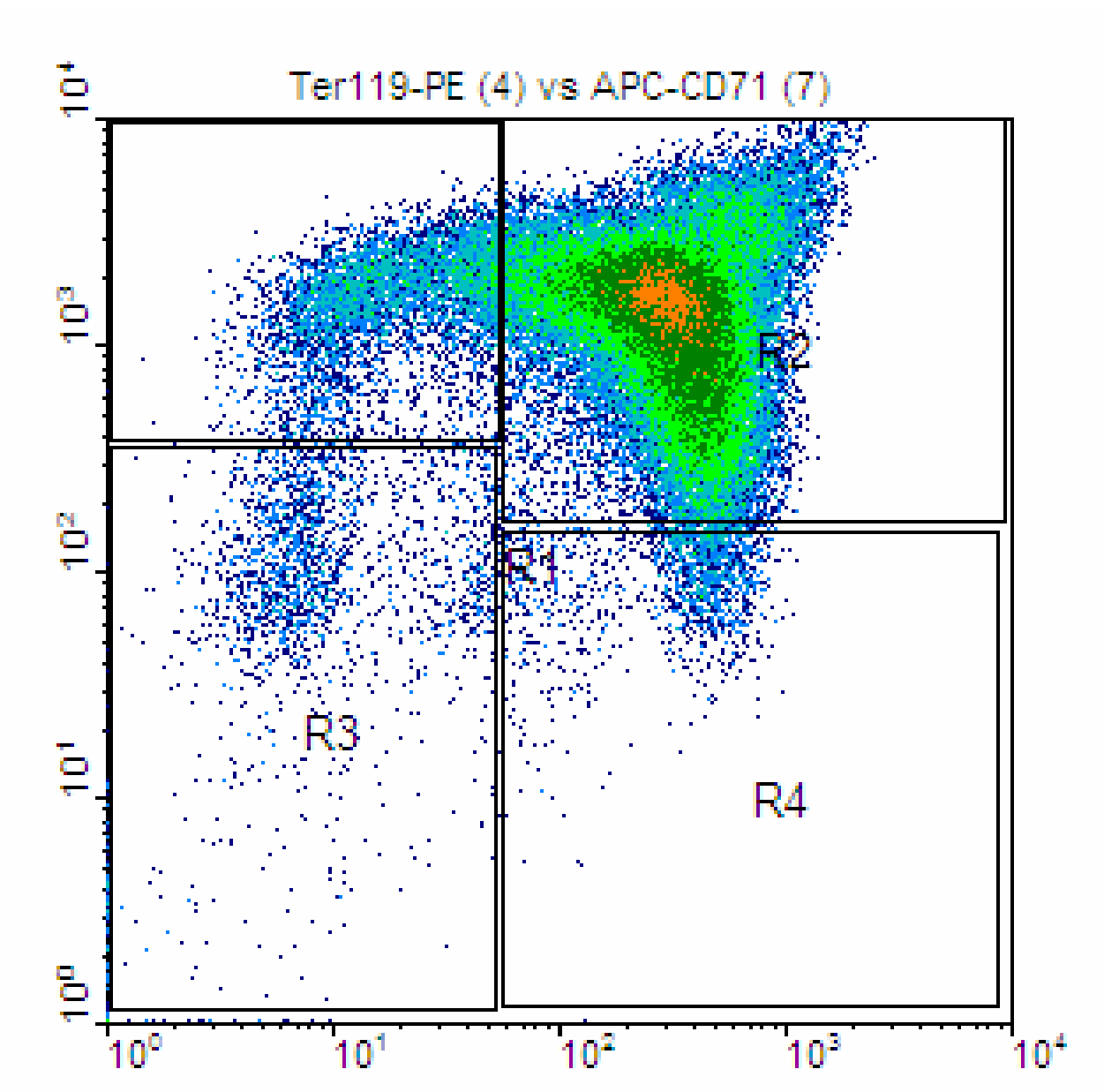

Quad	X-Mean	Y-Mean	Events	**%Gated**
(1) UL	17.6	1455.9	11624	**11.62**
(2) UR	308.7	1234.3	75444	**75.44**
(3) LL	9.3	60.9	7647	**7.65**
(4) LR	269.7	66.6	4485	**4.49**

Supplementary Figure 8. Gating Statistics for E14.0

1.1.5 Embryonic Day 14.5

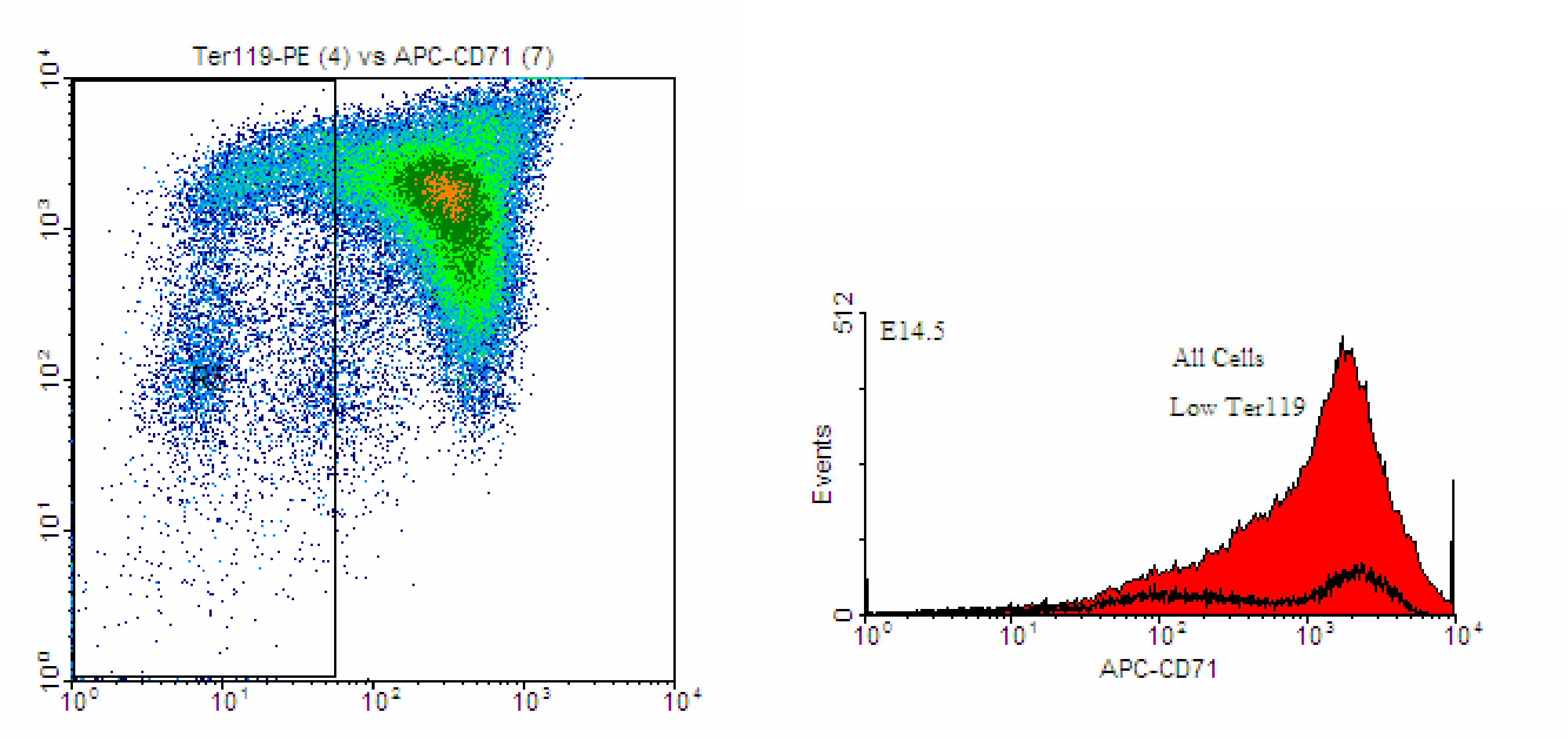

Supplementary Figure 9. Low CD71 for E14.5

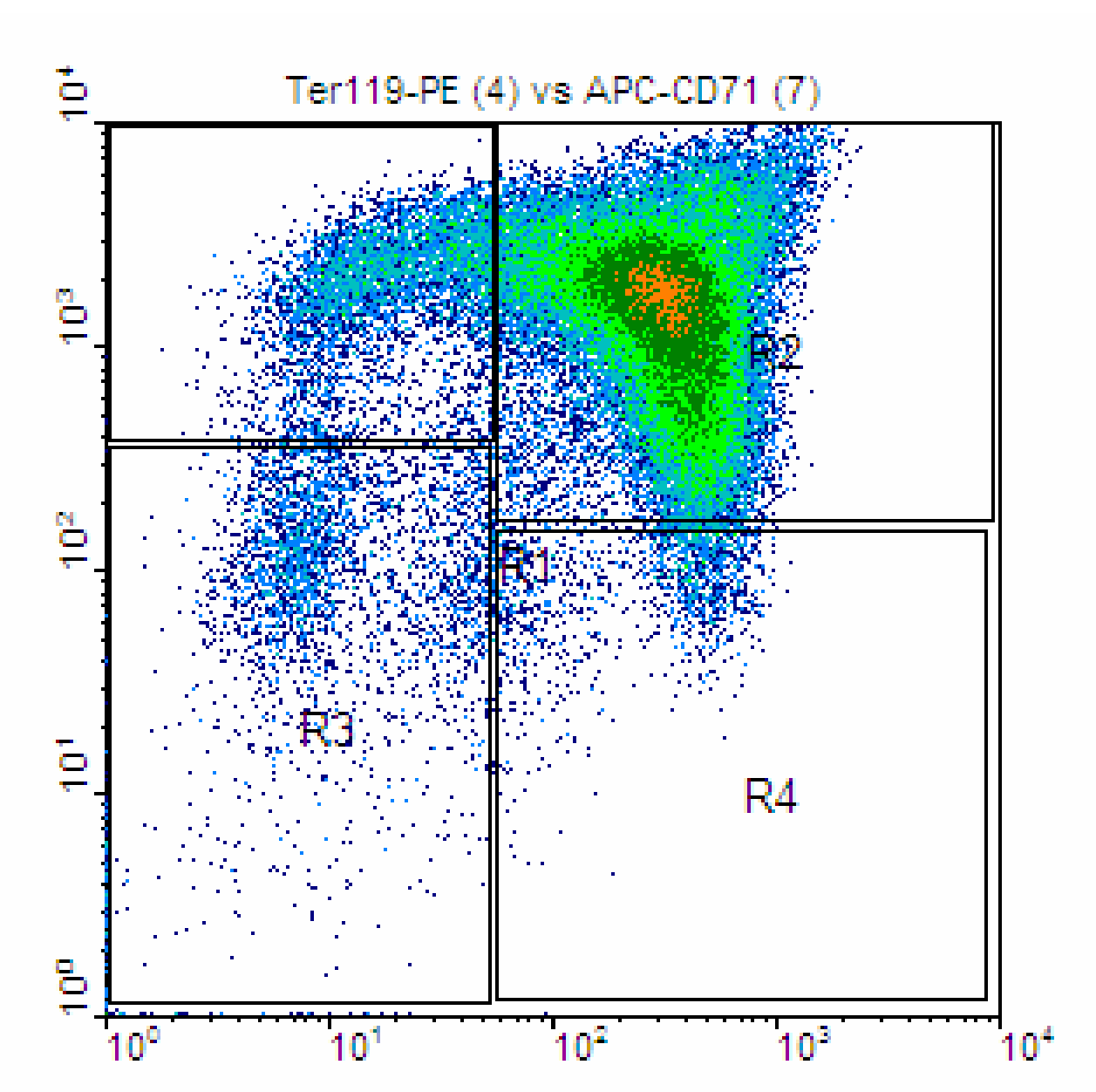

Quad	X-Mean	Y-Mean	Events	%Gated
(1) UL	18.0	1661.1	11879	**11.88**
(2) UR	306.5	1356.0	72359	**72.36**
(3) LL	10.6	59.9	10093	**10.09**
(4) LR	240	61.3	4671	**4.67**

Supplementary Figure 10. Gating Statistics for E14.5

1.1.6 Embryonic Day 15.0

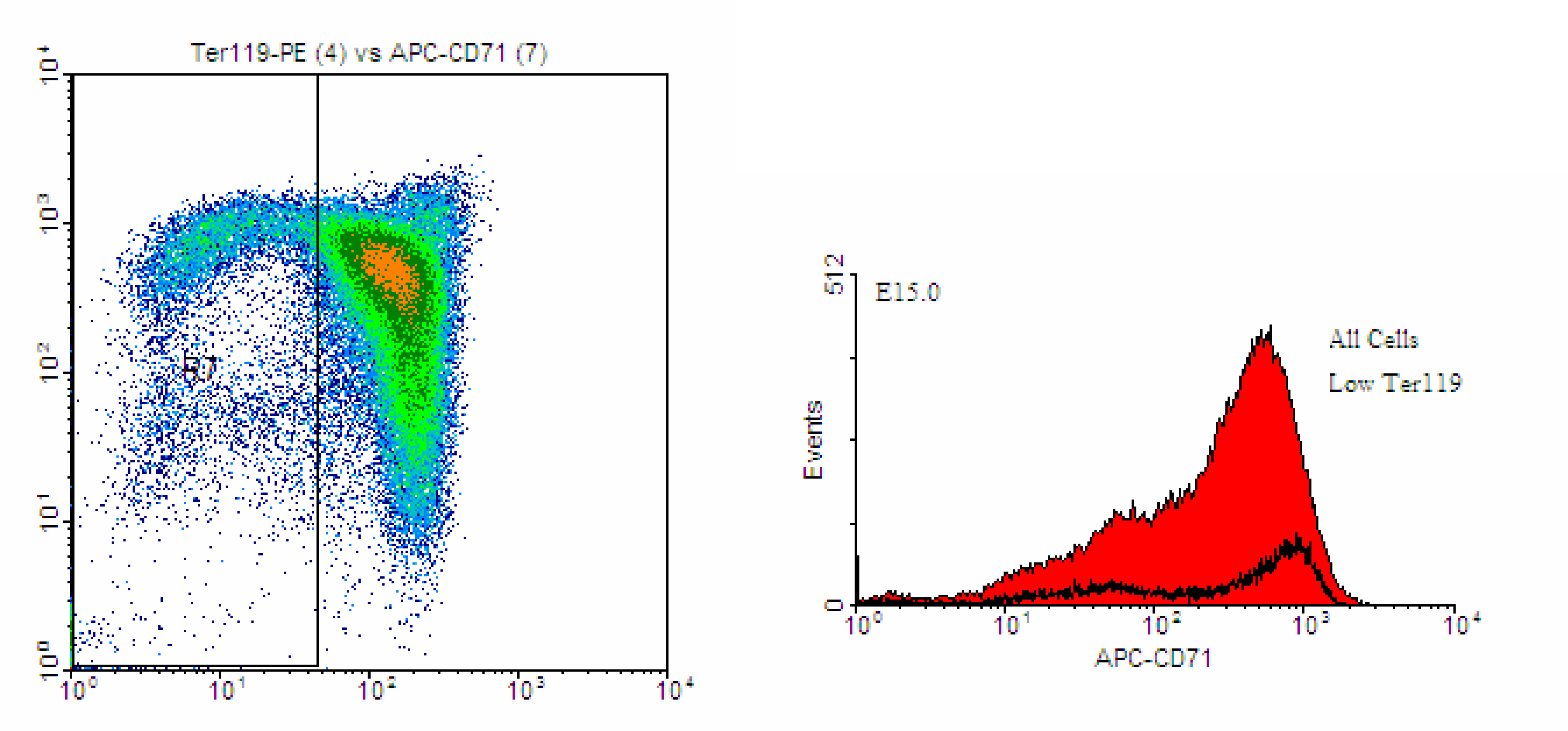

Supplementary Figure 11. Low CD71 for R15.0

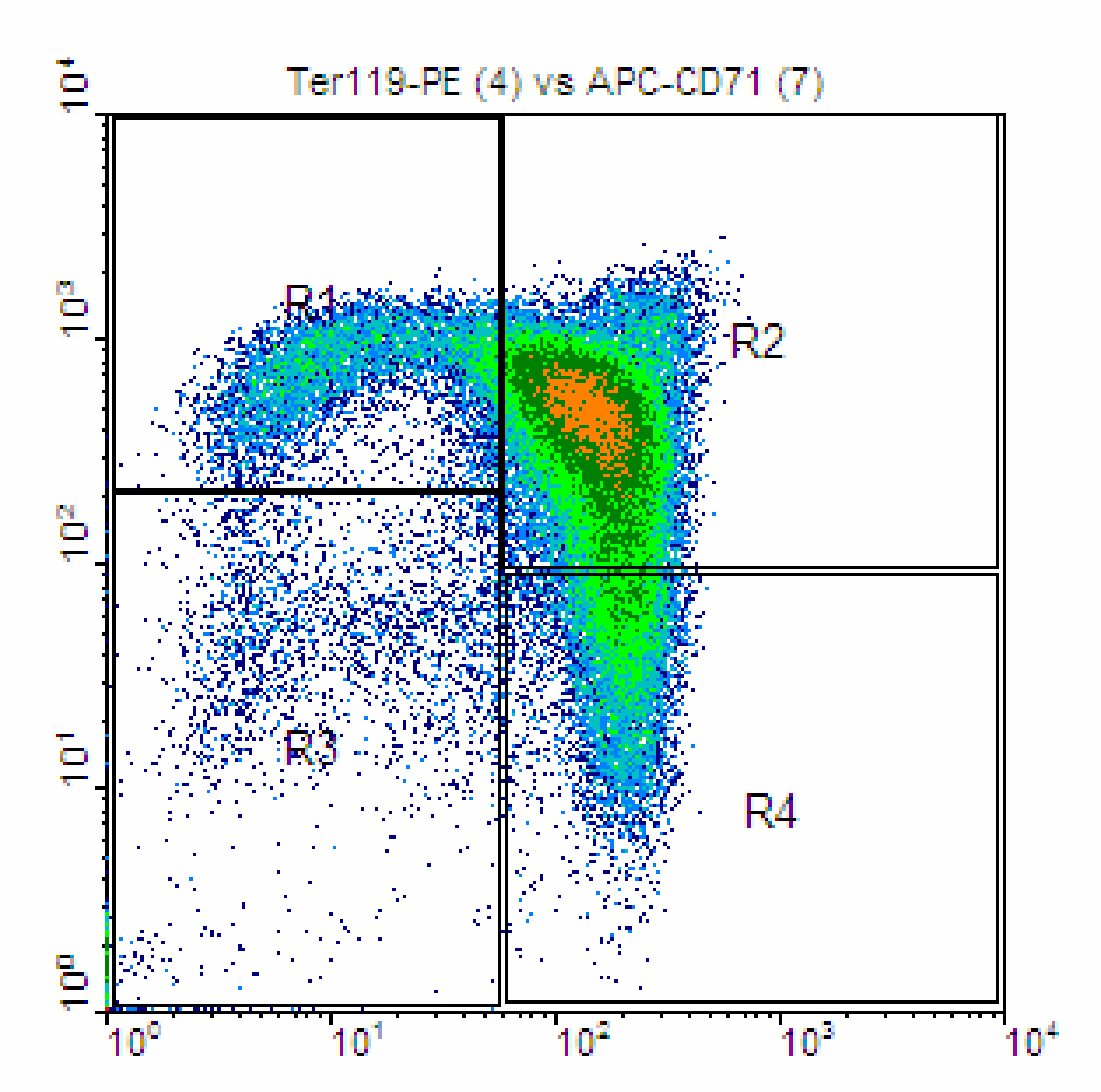

Quad	X-Mean	Y-Mean	Events	**%Gated**
(1) UL	14.3	661.4	13815	13.82
(2) UR	151.7	388.3	59028	59.03
(3) LL	10.1	32.9	9422	9.42
(4) LR	185.1	31.2	16542	16.54

Supplementary Figure 12. Gating Statistics for E15.0

1.1.7 Embryonic Day 15.5

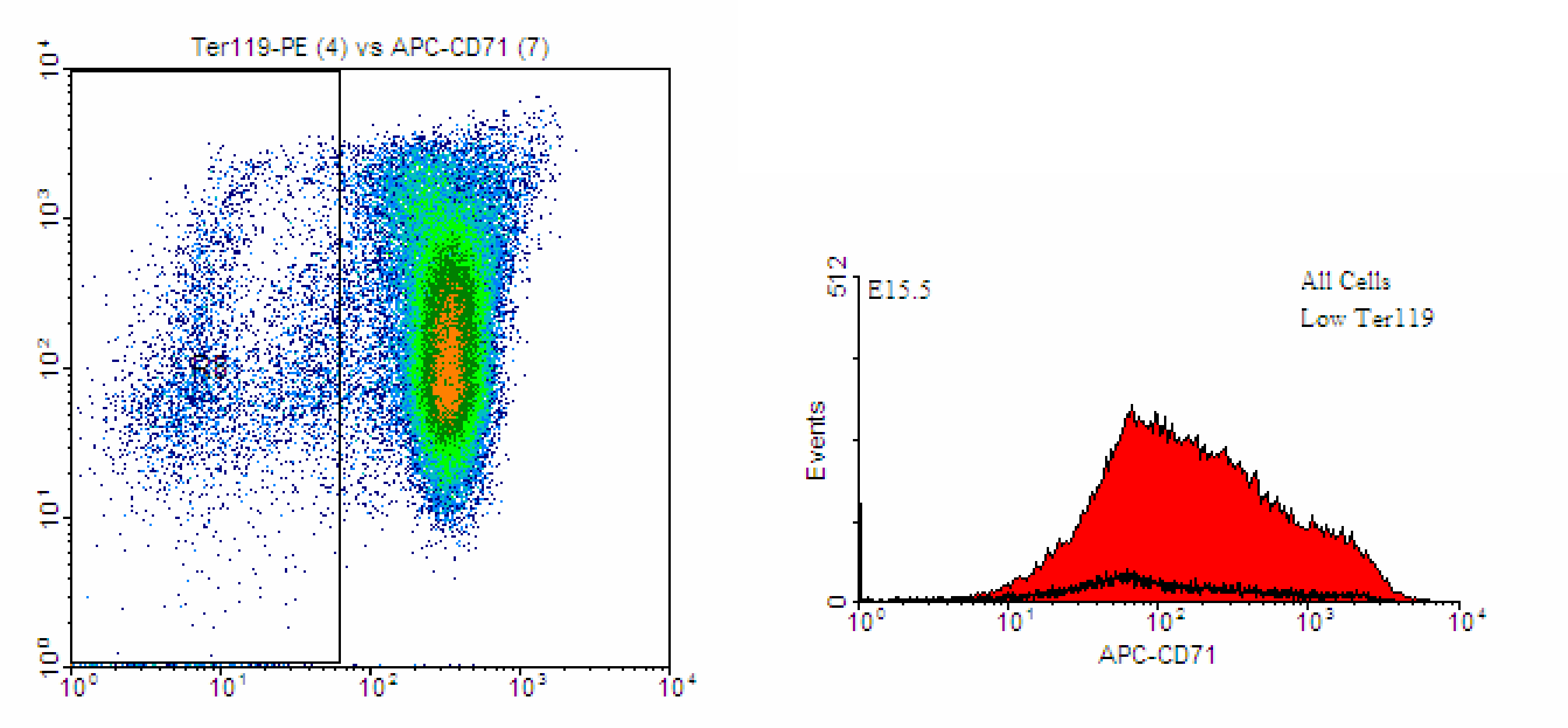

Supplementary Figure 13. Low CD71 for E15.5

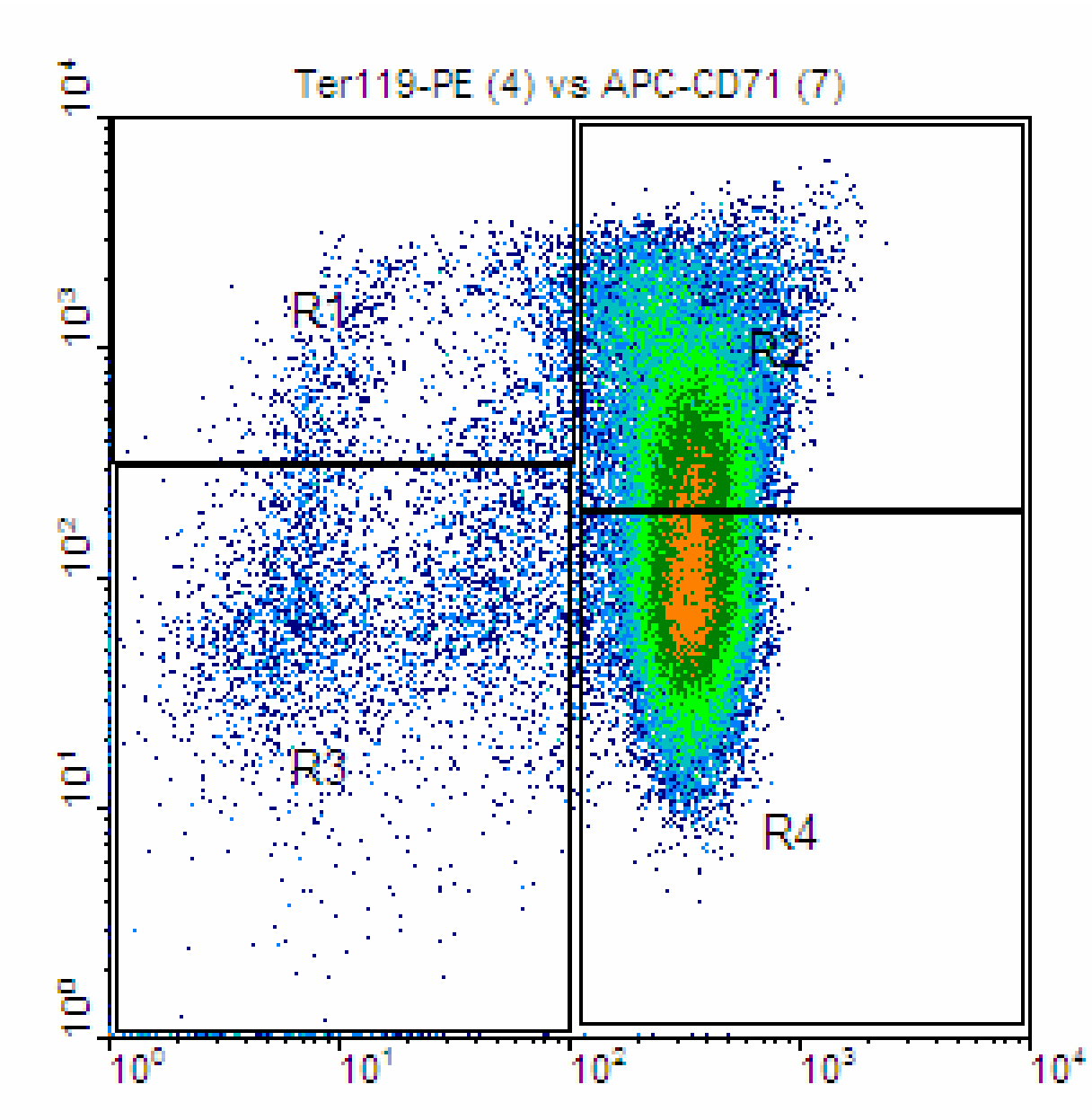

Quad	X-Mean	Y-Mean	Events	%Gated
(1) UL	25.8	885.1	5038	5.04
(2) UR	342.5	555.2	37889	37.89
(3) LL	14.3	50.4	11692	11.69
(4) LR	331.0	69.6	45985	45.99

Supplementary Figure 14. Gating Statistics for E15.5

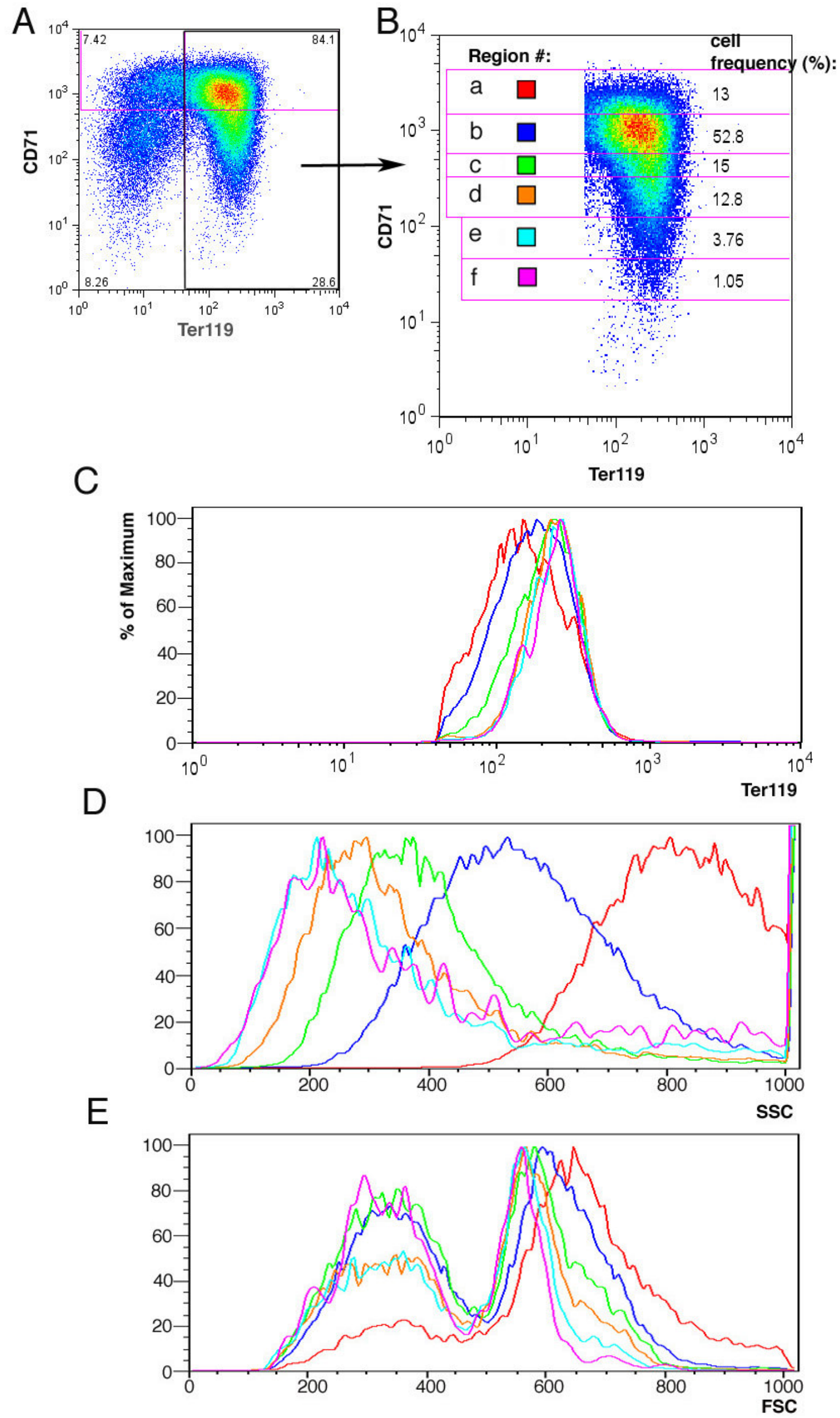

Supplementary Figure 15. Determination of the boundary between States 3 and 4

E14.5 fetal liver cells were labeled with antibodies against CD71 and Ter119. Viable cells (negative for the DNA dye 7-AAD) were selected (A). Ter119-high cells were gated and divided into regions of decreasing CD71 expression labeled *'a'* to *'f'* and color-coded as indicated (B). Histograms of Ter119 expression (C), side-scatter (SSC, D), and forward scatter (FSC, E) were constructed for cells in each region. These indicate a gradual increase in the mean Ter119 expression, reaching a maximum in regions *'e'* (light blue) and *'f'* (violet). Regions *'e'* and *'f'* also have similar histogram characteristics with respect to SSC, a function of cellular complexity, where they both reach a minimum. The FSC histograms demonstrate two populations, related to the cell-cycle status of the cells. Cells in S-phase and G2/M are likely to make up the higher FSC population. The position of the peak in the higher FSC population is related to cell size, and is the lowest for cells in regions *'e'* and *'f'*. Cells in regions *'e'* and *'f'* also have the highest proportion of cells in the lower FSC population. Taken together, cells in regions *'e'* and *'f'* represent the most mature erythroid precursor cells with relatively uniform characteristics. We therefore chose cells in regions *'e'* and *'f'* to constitue state 4, and the boundary between regions *'e'* and *d'* in this figure to represent the boundary between states 3 and 4.

Each state now represents a *proportion* of the overall cell population, determined by the gating coordinates of Ter119 and CD71 expression (Table 1). Due to rounding inherent to the analysis in the WinMDI software, the data was re-normalized, making a small adjustment on the magnitude of each of the states. The results are shown together in Table 1, which was used for the subsequent analysis. We have also altered these data somewhat to test the sensitivity of results to various alterations of this dataset, as explained below.

Table 1. Normalized distribution of cells among states

Time, days	State 1	State2	State 3	State4
0	0.345	0.655	0	0
0.5	0.162	0.262	0.551	0.025
1	0.134	0.236	0.610	0.020
1.5	0.076	0.117	0.761	0.046
2	0.102	0.120	0.731	0.047
2.5	0.095	0.140	0.597	0.168
3	0.116	0.050	0.377	0.457

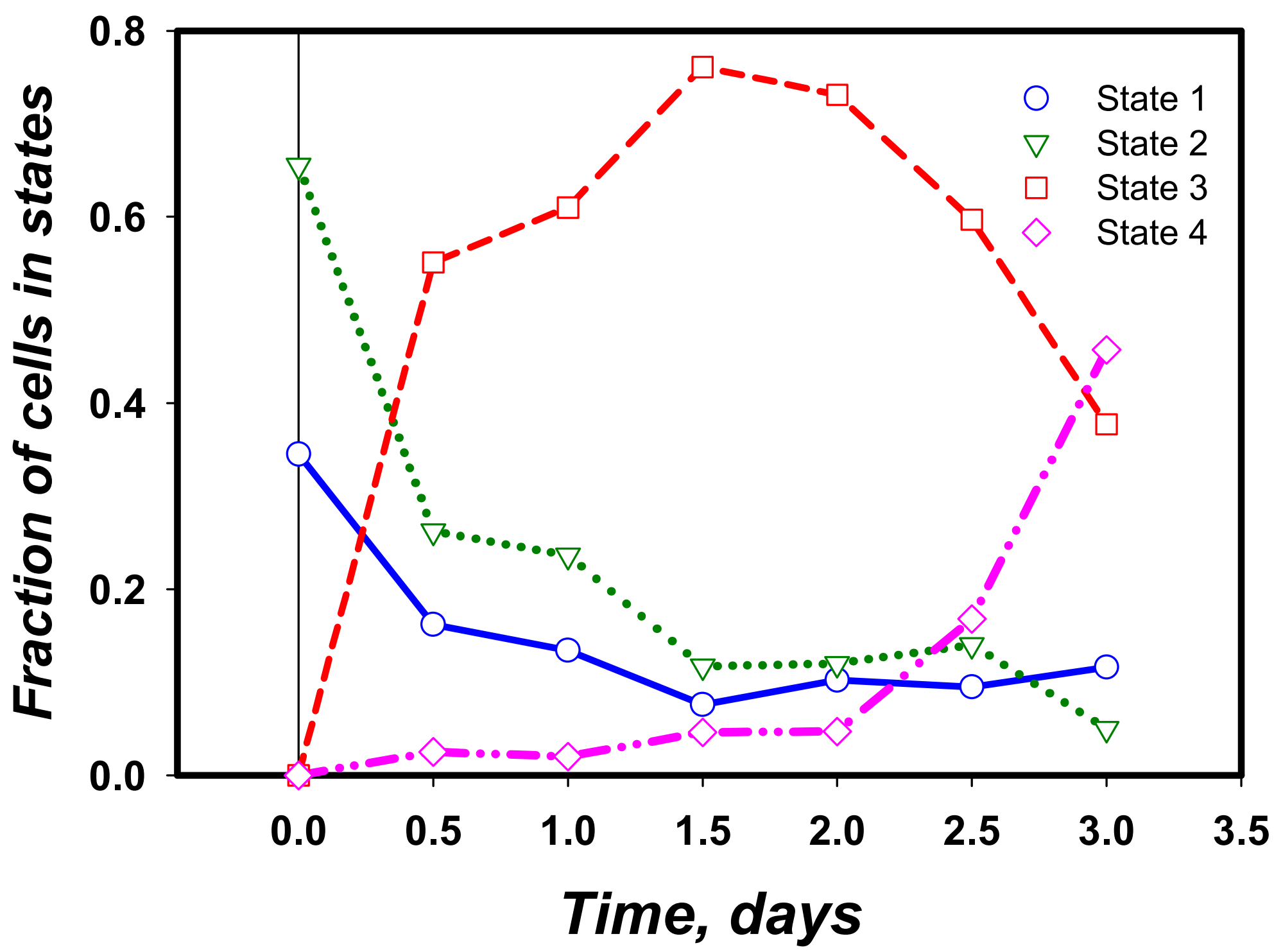

Supplementary Figure 16. *In Vivo* Embryonic Time Course of Changes in Relative Cell Numbers in Each State

Fractions of cells in each state are determined based on Supp. Figs. 1-15.

Based on these experimental data, we wanted to determine the most likely underlying mechanisms of interaction between cells in different states. We approached this problem by testing in a comprehensive fashion different hypotheses, each assuming different number and types of interactions between cells in states 1 through 4. These hypotheses were expressed as mathematical models in the form of ordinary differential equations (ODEs). We have thus explored a wide space of potential cell-cell interaction 'topologies', limited only by the combinatorial possibilities. As explained more in detail below, these models were tested for their ability to both represent the experimental data accurately, as well as remain resistant to uncertainty in model parameter values. The main assumption of our analysis was that interactions between cells in different states frequently present in 'good' models ('goodness' of a model will be further defined below).are also present with high probability in the real system. Furthermore, to deal with uncertainty in the experimental measurements, we identified the cell-cell interactions that are robust to variation of times assigned to specific measurements, and to other alterations to the basic experimental data set. The overall analysis was manifested as a large scale computational process that iteratively constructed the hypothetical models, and tested them for suitability for representing the true control architecture of red blood cell differentiation.

1.2 Model Development

1.2.1 General mathematical description

In flow cytomtery experiments, the data corresponding to different parts of the resultant histogram are commonly represented as percentages of the total number of cells in a sample analyzed. The sample size normally does not carry any information about the absolute number of cells in the tissue (e.g., liver, spleen or bone marrow). Thus we have dealt with essentially normalized data sets for the numbers of cells in each state and at each time point. We have expressed this fact by normalizing the total number of cells in all states at each time point to unity.

As described in detail below, we analyzed 298 possible model network topologies defined by what inter-state cell-cell interactions can take place. A common feature of all these networks is the core assumption of irreversible transfer of erythroid progenitors from state 1 to state 2 to state 3 to state 4 during differentiation. To each network topology we have assigned a system of ordinary differential equations describing how the time derivatives of the *normalized* numbers of cells in each state depend on the numbers of cells in other states and the particular specifics of assumed cell-cell interactions (see Eq. 1 below). These equations can be expressed as a set of dependencies of cells in each state on the vectors of normalized cell numbers in different states and vectors of parameters determining state transitions, i.e., as vector fields.

The vector field for every system of equations is thus a balanced input-output relationship governed by the nonlinear rates of transition, $h_{i,i+1}$. The system is mass-balanced, i.e. we assume that the total number cells at each time point remains unity. We assume that the only driving force is the initial conditions, as there are no exogenous inputs nor is there exit from the system, i.e., the cells in state 4 are assumed to be in the final differentiation state on the time scale considered.. Further constraints were added in subsequent analysis to retain non-negativity of both states and transitions, as described below.

Equations 1. Generalized Model Vector Field

$$\frac{dx_1}{dt} = -h_{12}(\vec{x}, \vec{p})$$
$$\frac{dx_2}{dt} = h_{12}(\vec{x}, \vec{p}) - h_{23}(\vec{x}, \vec{p})$$
$$\frac{dx_3}{dt} = h_{23}(\vec{x}, \vec{p}) - h_{34}(\vec{x}, \vec{p})$$
$$\frac{dx_4}{dt} = h_{34}(\vec{x}, \vec{p})$$

In Eq. 1, the state variables (or simply states) x_i represent the proportions of cells in each of the above defined differentiation states. It is assumed that there are transitions between states representing steps in cell differentiation. The transition rates, $h_{i,i+1}$, are the rates, with which cells can transit from state to state. They are subject to directionality condition due to the presumed absence of de-differentiation. The transition rates can depend on the state variables in different ways. If there are no cell-cell interactions involving the two states, between which the transition occurs, the rate of transition is simply proportional to the state variable of the 'source' state, from which the transition occurs. If there are cell-cell interactions, than this rate can depend on products of the 'source' state variable and other state variable or on the square of the 'source' state variable. Thus $h_{i,i+1}$ can be a nonlinear function of any state, always containing a term reflecting a first-order state dependency (due to baseline differentiation rates unmodulated by inter-cellular signaling) and, possibly, a sum of state variable products and quadratic state dependencies. The first three parameters: {p_1, p_2, p_3}, representing the rates of unregulated differentiation (linear state functions described above) are present in each model topology, and are always non-negative. The transition function $h_{i,i+1}$ is also influenced by a second type of interaction, due to feedback or feedforward regulation of cells in the source state, x_i , by cells in any other state, x_j **.** The effect of such an interaction on the transition function may be either positive or negative, and is proportional to the product of the cells in the two interacting states, $x_i x_j$. In the special case where $j=i$, the interaction is autoregulatory and is proportional to x_i^2. Twelve parameters, p_k $(k \in 4\text{-}15)$, represent the rate constants for each of these potential interactions, which are classified as feedforward [ff$(j, i+1)$, if $j \leq i$] or feedback [fb(j, i), if $j>i$] interactions, as detailed in Supplementary Table 3. The transition function $h_{i,i+1}$ is therefore the sum of all these potential interactions (Eq. 2 below). Each of the feedforward or feedback inter-state interaction parameters (p_4-p_{15}), can be positive or negative, to be determined by regression for each model topology, and thus are of sign that can vary with the model topology. Together, these parameters contribute to each overall state transition, and define the "connectivity" or "structure" of each hypothetical model.

Equation 2. Generalized Transition Function

$$h_{i,i+1}(\vec{x}, \vec{p}) = p_i x_i + p_i \sum p_k x_i x_j$$

The level of abstraction for the generalized transition function is partially based on the fact that the experimental dynamics are simple, and should not require long series and sums in order to reproduce them computationally.

1.2.2 Generation of different 'model topologies'

In all, in our analysis, 298 different possible differentiation network models and the corresponding vector fields were constructed by 'turning on/off' each of 12 different parameters regulating up to three nonlinear state dependencies (representing cell-cell interactions between cells in states 1-4) that we assumed could exist simultaneously in one model. The assumption that the number of regulatory interactions is limited to three is based both on the desire to limit the combinatorial explosion in the number of possible state dependencies, and on the more general argument that multiple and dense interactions between elements of any network can lead to instabilities (*2*). In a similar fashion, a recent network reconstruction analysis considerably limited the number of possible links in potential networks, obtaining nevertheless excellent results (*3*). More precisely, the model networks were constructed and investigated as follows. As there are 4 distinct erythropoieic states (as defined above), there can exist 12 different potential interactions between these states (each state can interact with 3 others). These interactions can be both positive and negative, further diversifying possible network topologies. We opted to allow the sign of each parameter to be determined by data regression, and allowed only one, two, or three total connections to exist simultaneously in any individual model. This reduces our total network model number (without explicit specification of the sign of each interaction) to:

Equation 3
$$\binom{12}{1}+\binom{12}{2}+\binom{12}{3}=12+66+220=298$$

1.2.3 Model fitting to the experimental data

Each vector field varying in the structure of its transition functions was generally described by templates of 1's and 0's which signal to the algorithm to either vary the parameters to minimize an objective function (Equation 4) consisting of similarity and penalty functions, or remain zero, thereby defining a characteristic regulatory scheme. The similarity function $f(\bar{x},\bar{p})$, is the squared difference between the experimental data and each fitted trajectory. It represents the sum of the squared difference between the experimental data and the fitted curves for all 4 states (f is a single value – the sum of the squared residuals for all four curves at once, for a total of 28 points). This fitting was performed for each separate model. The fitting was performed using different methods, including Gradient Descent and Random Parameter Search with similar results, but the more efficient algorithm that was also more effective in terms of avoiding local minima in optimization was Direct Version 3.2 written by Dan Finkel (http://www4.ncsu.edu/~definkel/research) based on (*4*). This algorithm was thus chosen as the main fitting engine to be used in the study. It was designed to solve global optimization problems with bound constraints and a real-valued function. The algorithm (abbreviated from **DI**viding **RECT**angles), a modification of the Lipschitzian optimization, globally converges to the minimal value of an objective function given the boundaries on each parameter, and a set of input arguments defining how to carry out the optimization (i.e. the number of iterations and function evaluations, set

a 25 and 300, respectively in our simulations). Requiring no knowledge of the objective function gradient, the algorithm divides the parameter space (restricted by parameter boundaries) into equivalents of two-dimensional rectangular regions and evaluates the objective function at the center of each divided region. The algorithm chooses which of the regions contains the optimum, and then divides this region in the next iteration. This process continues until the parameter space is optimally defined, i.e. the function optimized approaches a constant minimum in a small region of parameter space. This algorithm was used as written, with only minor modifications as to how parameters are sent to the module. In addition to minimizing the difference between the experimental data and the simulated trajectory, the parameter set was also required to retain non-negativity of each state $\left|\gamma_1(\vec{x},\vec{p})\right|$, transition $\left|\gamma_2(\vec{x},\vec{h})\right|$, and first-order parameters (Equation 5). Each of these functions is a simple penalty function which finds the most negative value that violates the constraints, and calculates a weighted value that is added to f, to further constraint the parameter set, $\vec{p}$ (gives a number between 0 and 1, which can be further weighted by α and β) in this optimization procedure. Moreover, the algorithm was run for 300 function evaluations (which on average was roughly 100 iterations past convergence) to ensure that each model parameter set had reached convergence. The primary reason for using this global optimization method was to avoid the potential systematic error of landing in local minima repeatedly, thereby severely biasing the results.

Equation 4. Parameter Determination

$$\min\left(f(\vec{x},\vec{p})+\alpha\left|\gamma_1(\vec{x},\vec{p})\right|+\beta\left|\gamma_2(\vec{x},\vec{h})\right|\right)$$

Equation 5. Constraints on Parameters

$$\vec{x}\in[x_i\geq 0]\forall i=1,\ldots,4, \qquad \vec{p}\in[p_i\geq 0]\forall i=1,2,3, \qquad \vec{h}\in[h_i\geq 0]\forall i=1,2,3$$

Equation 6. Penalties for violating constraints

$$\gamma_1(\vec{x},\vec{p})=\exp(\min(x))-1, \qquad \gamma_2(\vec{x},\vec{h})=\exp(\min(h))-1$$

1.2.4 Multivariate sensitivity analysis

Once the parameter set defining the best fit to the experimental data for a particular network model ('fitted set') was determined, we performed multivariate sensitivity analysis based on a percent change of the all the parameters in the fitted set varied simultaneously. Since we were interested in the local control properties for each parameter, only small, local perturbation of 5% of the nominal parameters value (obtained from fitting) were tested. The purpose was not to find bifurcation boundaries or other features of the global system behavior, as we were not interested in cases where the simulated dynamics were dissimilar to the experimentally observed behavior. The only criterion was that the perturbation be sufficient to determine sensitivity of a given model to variation of different parameters. Thus, for each model we compared the results of 200 distinct implementations, each time sampling the parameter values from a uniform distribution contained within +/- 5 % of the nominal value found in the fitted set. In the event that a parameter set broke any of the aforementioned

constraints, the parameter set was discarded, and another simulated in its place. For each parameter generation, the resultant trajectories describing the proportions of cells in each state were compared to the corresponding experimental data, and the sum of squared residuals (SSR) value was computed for all four curves representing the evolution of all four states (Supplementary Supplementary Figure 16). The results were assembled for all 298 topologies to be used in subsequent model ranking and parameter control analysis (Section 3, below).

In addition, for each of the 200 simulations, and 200 corresponding SSR values, a correlation coefficient was calculated for the values of each parameter and the corresponding SSR metric. To ensure that the statistics didn't reflect unbounded trajectories, each trajectory was checked for non-negativity, both in the state dynamics x, and in the state transitions, h. In the event that a simulated trajectory or its transition was negative, that parameter set was discarded, and another was generated.

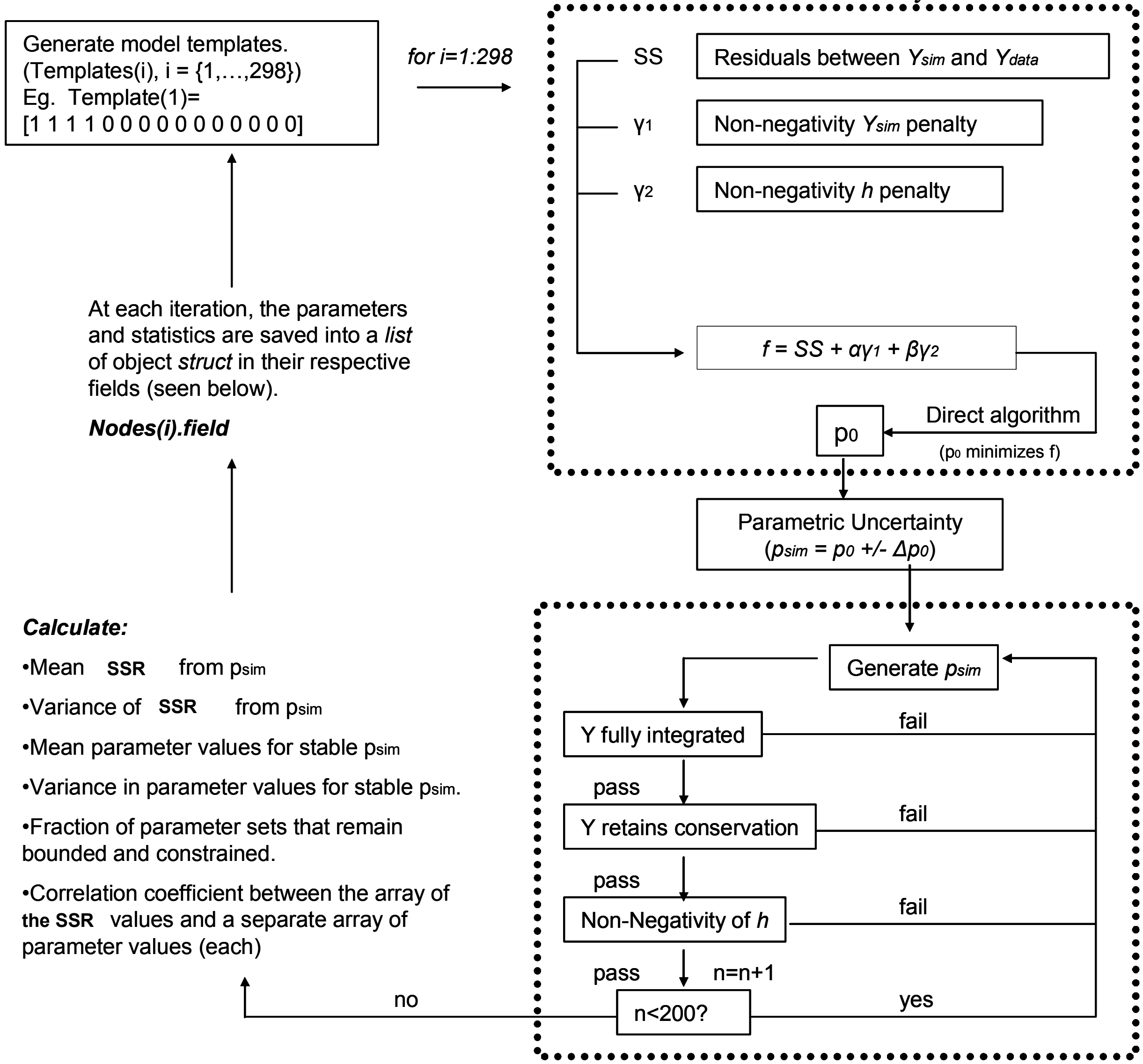

node_number:	1
parameters:	[0.4870 1.4609 0.2263 -0.5947 0 0 0 0 0 0 0 0 0 0 0 0]
lower_bounds:	[0.4626 1.3879 0.2150 -0.6244 0 0 0 0 0 0 0 0 0 0 0 0]
upper_bounds:	[0.5113 1.5340 0.2377 -0.5649 0 0 0 0 0 0 0 0 0 0 0 0]
sum_residuals*:	0.8742
Rsquare_val:	0.8578
param_means:	[0.4865 1.4656 0.2259 -0.5968 0 0 0 0 0 0 0 0 0 0 0 0]
param_vars:	[2.1660e-004 0.0016 4.6413e-005 2.9153e-004 0 0 0 0 0 0 0 0 0 0 0 0]
rsq_mean:	0.8561
rsq_var:	8.9120e-006
stability:	1
correlation:	[0.8552 0.0210 -0.2966 0.2573 0 0 0 0 0 0 0 0 0 0 0 0]
num_variations:	200
Significant:	'not'
Transitions:	'passed'

*Here, the sum of residuals is the residuals + the penalties (which should be 0 if they pass the "Transitions test")

Supplementary Figure 17. Simulation Process Flow Chart. The dashed boxes represent iterative processes for *each* model template.

To implement the above procedures, we developed a MATLAB package (Mathworks, Inc., v6.1, release 12.1, available upon request) that iteratively constructed different polynomial vector fields representing different hypothetical

a

FB32 (+)

$\dot{x}_1 = -.56*x_1$

$\dot{x}_2 = .56*x_1-1.7*x_2*(1.+.50*x_3)$

$\dot{x}_3 = 1.7*x_2*(1.+.50*x_3)-.19*x_3$

$\dot{x}_4 = .19*x_3$

FB31 (+), FB41 (-), FB43 (-)

$\dot{x}_1 = -1.3*x_1*(1.+.50*x_3-7.2*x_4)$

$\dot{x}_2 = 1.3*x_1*(1.+.50*x_3-7.2*x_4)-2.4*x_2$

$\dot{x}_3 = 2.4*x_2-.56*x_3*(1.-5.6*x_4)$

$\dot{x}_4 = .56*x_3*(1.-5.6*x_4)$

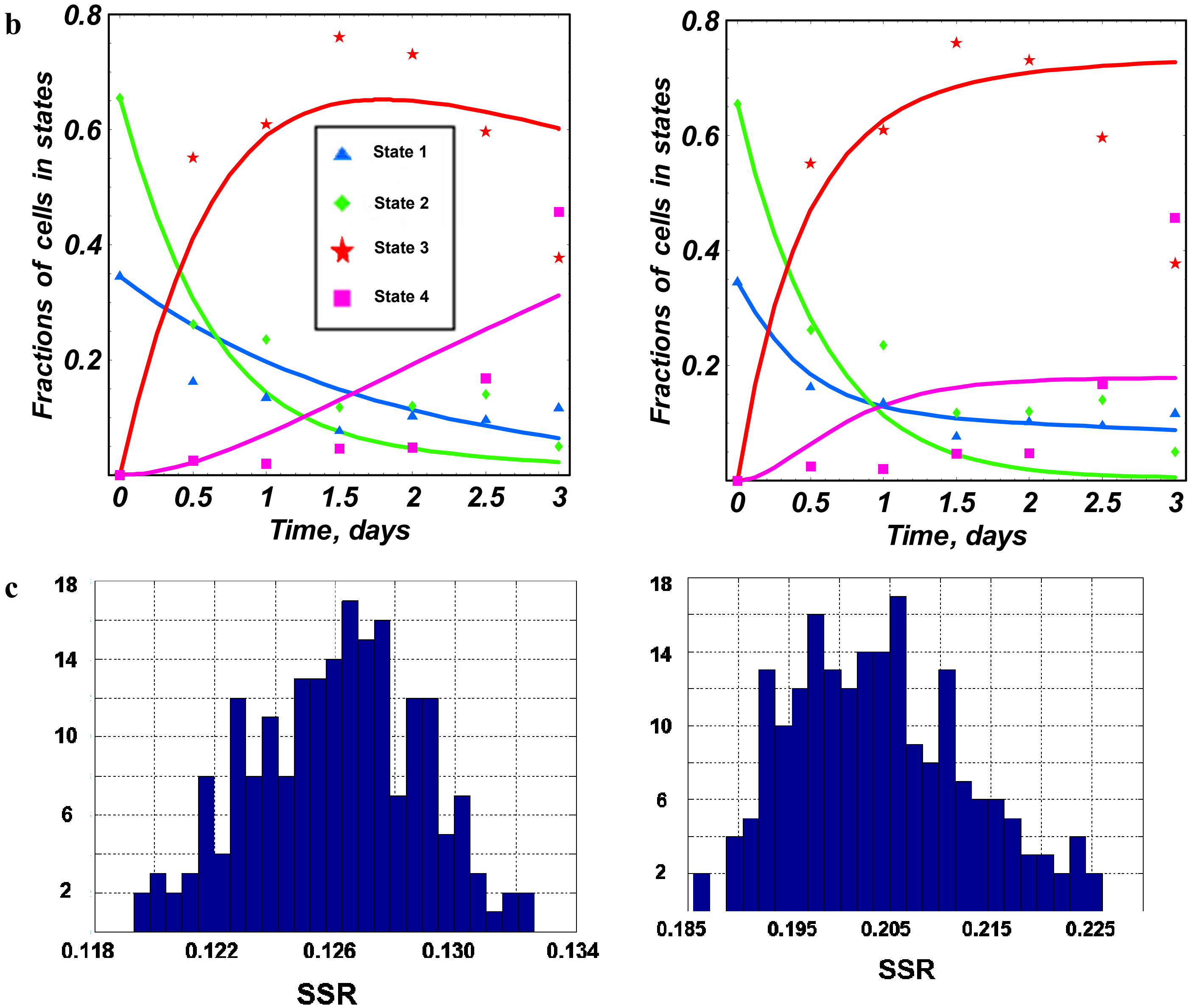

Supplementary Figure 18 Model Construction. a) Examples of two hypothetical models representing different assumed interactions with coefficient values reflecting the best fit to the experimental data; b) Experimental data plotted with fitted curves described in a). Depending on the particular set of feedback loops, a model can result in a superior or inferior fit, which then influences its ability to represent the experimental data subject to perturbation; c) Distribution of the Sum of Squared Residuals (SSR) corresponding to the models in a), given 5% parametric uncertainty. SSR is calculated between experimental data and simulated model curves, as we are only interested in the ability of each topology to represent the experimental data. This figure is a part of Fig. 2 of the main text.

models, each time performing a nonlinear regression to identify model parameters and numerical simulations for multivariate parameter sensitivity analysis. The general process for simulating each model topology is mapped out in Supplementary Fig. 17 and illustrated for two different model topologies in Supplementary Fig. 18.

1.3. Model Discrimination and Rank-Ordering

As a result of model generation described in Section 2.2, we obtained model trajectories corresponding to 298 x 200 = 59,600 implementations of 298 distinct model topologies. Comparison of these trajectories to the experimental data yielded values of the sums of squared residuals (SSR) corresponding to each implementation. An mentioned above, the main hypothesis of this study is that one can use these SSR values to determine the importance of different parameters defining cell-cell interactions between cells in different states for accurate and robust reproduction of the actual experimental dynamics. In other words, we hypothesize that a) the parameters defining different interactions can be rank-ordered in terms of their contribution to the fit and the robustness of the fit and b) the higher the parameters are on this rank-ordered list, the more likely it is that the corresponding cell-cell interactions are present in the real developmental system. In this section we detail the procedures that are based on this hypothesis.

The 200 implementations obtained for each studied topology result in 200 SSR values which can be binned and plotted as a histogram (see e.g., Supplementary Fig. 18). The peak of the histogram corresponds to the most common goodness of fit yielded by a particular model topology. The variance of the SSR value distribution given by the histogram reflects the sensitivity of the goodness of fit to parameter variation. The models with relatively low peak histogram values are considered to give better fits to the experimental data. The models with relatively low histogram variance values are considered to display robustness of the fit to parameter variation. Since one knows for all model topologies whether or not a specific interaction between states is present or not, one can ask the question: do certain parameters occur more frequently in models with better fitness and/or high robustness to parametric variation? If a quantitative metric giving an answer to this question is developed, one can rank-order the parameters according to the values of this metric and postulate the relative likelihood that a certain cell-cell interaction is present in the developmental system under investigation.

We thus used the properties of the histograms of the types shown in Supplementary Fig. 18 to define the metrics. The goodness of fit for a particular model was defined as the position of the peak of the histogram. Then for each parameter, we analyzed the goodness of fit of the models, in which the interaction described by this parameter is present. Since we wanted to give higher weights in the rank ordering to parameters occurring in models providing better fits, we have calculated for each parameter the following metric:

Equation 7 $$F_j = \sum_{Mi} \frac{1}{f_M},$$

where f_M is the mean of the distribution represented by the histogram of the SSR values for M-th model, for the *j*-th parameter of interest.

Another way of rank-ordering model parameters is to segregate the parameters, which appear to endow resistance to parametric uncertainty or robustness. The underlying assumption is that if a model should not only fit the data well, but also do so robustly, so that the fit is not easily altered if the parameters defining the model are slightly altered. Thus we again considered for each parameter *j* the models, in which this parameter is present. We further calculated the robustness metric R_j, which is the summation of the inverse variance values of the SSR value histograms corresponding to each of these models *M*.

Equation 8 $$R_j = \sum_M \frac{1}{v_M}$$

Therefore, the robustness metric value is greatest for the parameters participating in the models with fits generally resistant to parametric uncertainty. We have rank-ordered the parameters according to the metrics *F* and *S* and plotted the results in Supplementary Fig. 19. We have represented the relative input of models, in which the values of the corresponding parameters are either positive or negative, into the sums defining the metrics, by using different colors. Thus one can see whether the signs of the corresponding interactions are either positive or negative. One can see that 1) there is a good correspondence between two ways to rank-order the parameters and 2) the robustness metric has greater variability allowing for a better discrimination of the relatively important parameters. Thus we have chosen to primarily focus on the robustness metric in our further analysis.

A note on the analysis: Although it might appear that ranking models based on SSR statistics alone ignores the fact that different models have different numbers of parameters and therefore this analysis should be normalized by the number of parameters, as done in e.g., Akaike or Bayesian Information Criteria, this is in fact not true. We stress that our analysis does not rank models, but rather, it ranks interactions (the network links). For each such interaction, we calculate the metrics R_j and F_j that rely on the performance of the entire set of all models in which it is present. Because any given interaction participates in an equal number of models of any given complexity (that is, models that contain one, two or three interactions), the process of ranking interactions described below is properly normalized. Specifically, each of the 12 possible feedforward and feedbaclk interactions participates in 67 models, of which 1 contains that interaction alone, 11 contain that interaction together with one other, and 55 (=11x10/2) contain this interaction together with two other. Thus, although the analysis of specific models is uncorrected for the number of parameters, the analysis of the entire set of models containing a specific interaction is unbiased: each relies on the same number of models of any given complexity.

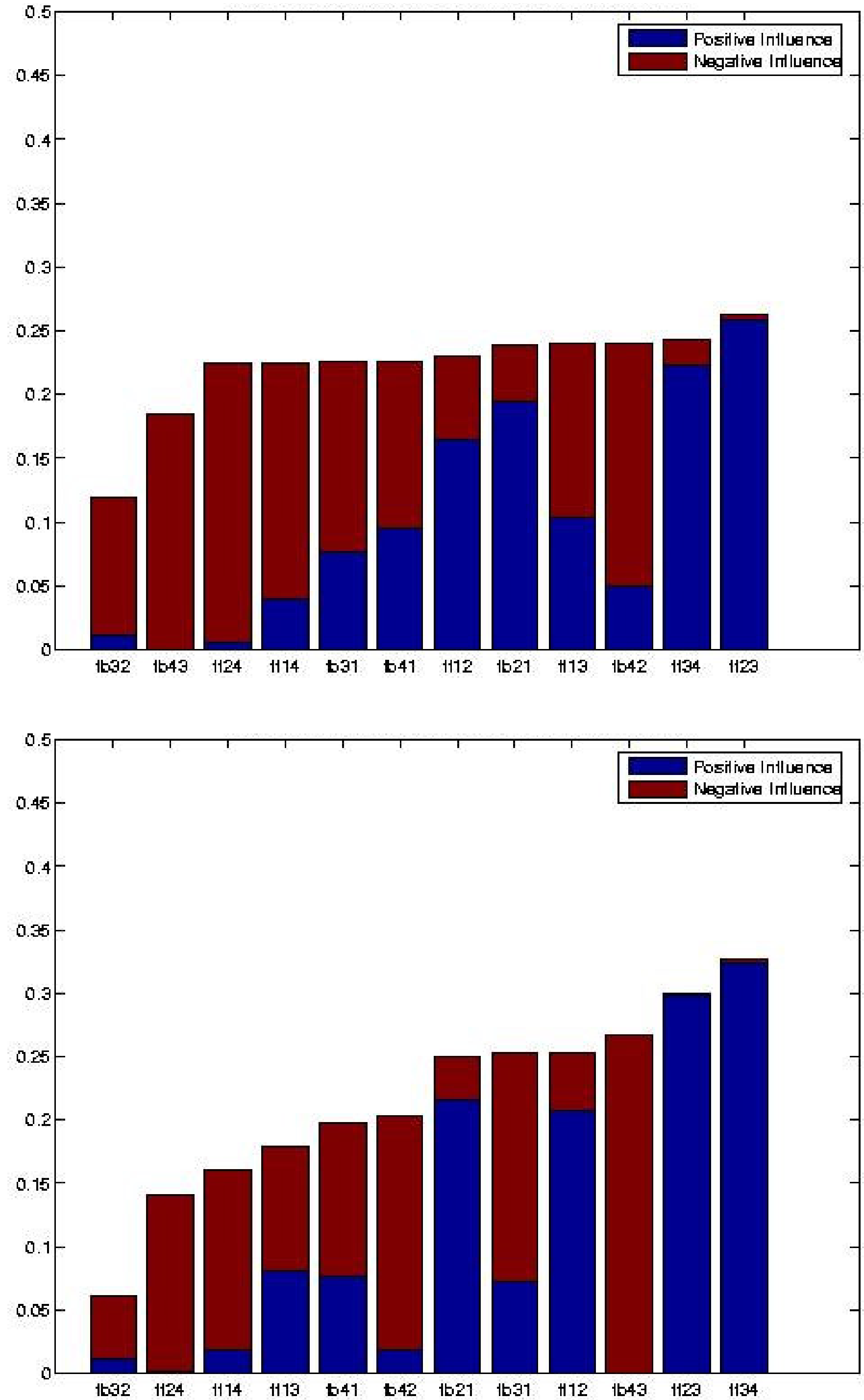

Supplementary Figure 19. Rank Ordering of Model Parameters by SSR Magnitude and Variance. The metrics F and R defined in Equations 7 and 8 are plotted on the Y-axes of the upper and lower panels respectively. The colors indicate the ratio of positive (blue) or negative (red) values of a given parameter in all the models, in which the state relationship characterized by this parameter exists. The state relationships are described as feedback (fb) and feedforward (ff) interactions. In feedback interactions the rate of transition *out of* the state denoted by the second number is modified in proportion to the number of cells in the state denoted by the first number (Equation 2). In feedforward interactions the rate of transition *into* the state denoted by the second number is modified in proportion to the number of cells in the state denoted by the first number (Equation 2). The bars are ordered according to the values of F and R for each parameter. This figure is a part of Fig. 3 of the main text.

Finally, one can determine variation of which of the parameters can affect the fit the most for a given model topology. Since for each topology we have both 200 different values of all the parameters defining this topology and 200 SSR values, we can determine the correlation coefficients between the parameter and SSR metric values. If the correlation coefficient is high, the parameter is likely to affect the model fit significantly, if it is low, the value of the parameter is of little consequence. This metric (*C*) thus represent the degree of ***control*** a parameter exerts on the goodness of fit. (This metric is distinct from the control coefficient used in Metabolic Control Analysis and usually defined as a fractional partial derivative of a metabolic flux with respect to the concentration of the enzyme or substrate participating in the reaction.)

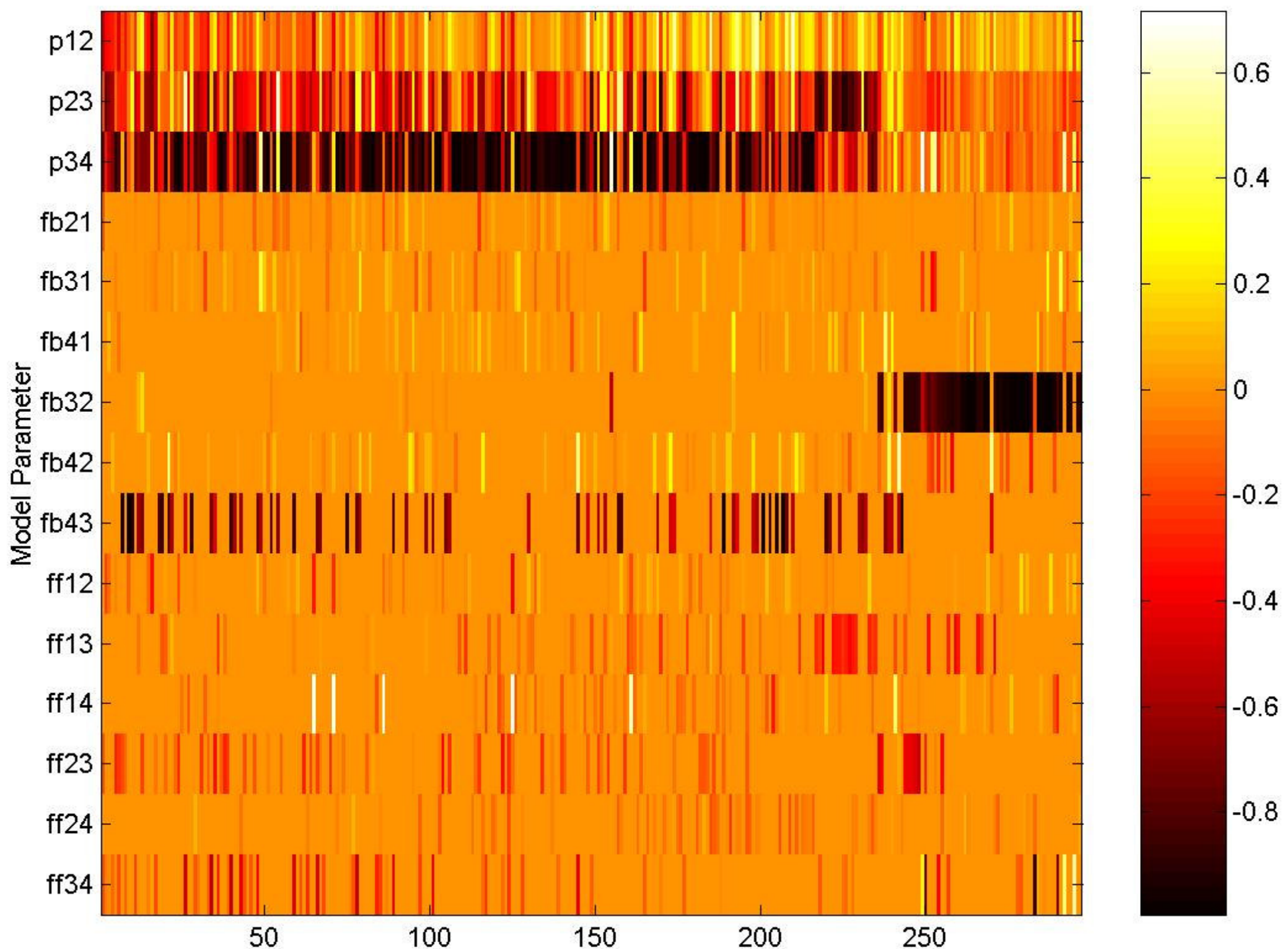

Supplementary Figure 20. The Correlation Matrix for 298 models analyzed. Each column represents a model, or regulatory topology, and each column is an interaction present in that model. The models are sorted by the variance in their SSR due to systematic parameter variation, in ascending order. The color bar indicates the value of the control metric defined as the correlation coefficient between the model parameter value and the SSR for each model. The interactions are defined as in Sup. Fig. 19, with the addition of p12, p23, p34 denoting the rates of transitions from the state indicated by the first number to the state indicated by the second number in the absence of additional feedback and feedforward interactions involving the corresponding states. These definitions are preserved in subsequent figures. This figure is a part of Fig. 3 of the main text.

Supplementary Figs. 19 and 20 suggest immediately that some parameters have significantly higher values of *S* than most other parameters, including ff23 (mostly positive), ff34 (mostly positive) and fb43 (mostly negative). The opposite is true of fb32 (mostly negative). Additionally, variations of all these parameters from their nominal values negatively affects the fit, according to the negative values of C in Fig. 20. From Fig. 20 it also becomes more evident that ff23, ff34 and fb43 are

present more frequently in more robust models, whereas fb32 is present mostly in the least robust models. The rest of the parameters show mixed distributions and often mixed sign in Supplementary Fig. 20, making any judgment about their putative roles in the underlying erythropoiesis network difficult. These preliminary results were tested by determining their sensitivity to alterations of either the experimental dataset or the methods of fitting and analysis (Section 4). The final discussion of results was made following this further analysis.

Since each fitting procedure also results in specific values of the parameters in various models, we have plotted the values of the parameters to determine if any characteristic values emerge (Supplementary Fig. 21). The interesting features of this dataset include relative magnitude of transition rates: p23 > p12 > p34. Additionally, it shows relatively high magnitudes of fb43, ff23 and fb32. As noted above, fb32 occurs mostly in models of low rank and thus probably does not occur in the true erythropoiesis regulatory network.

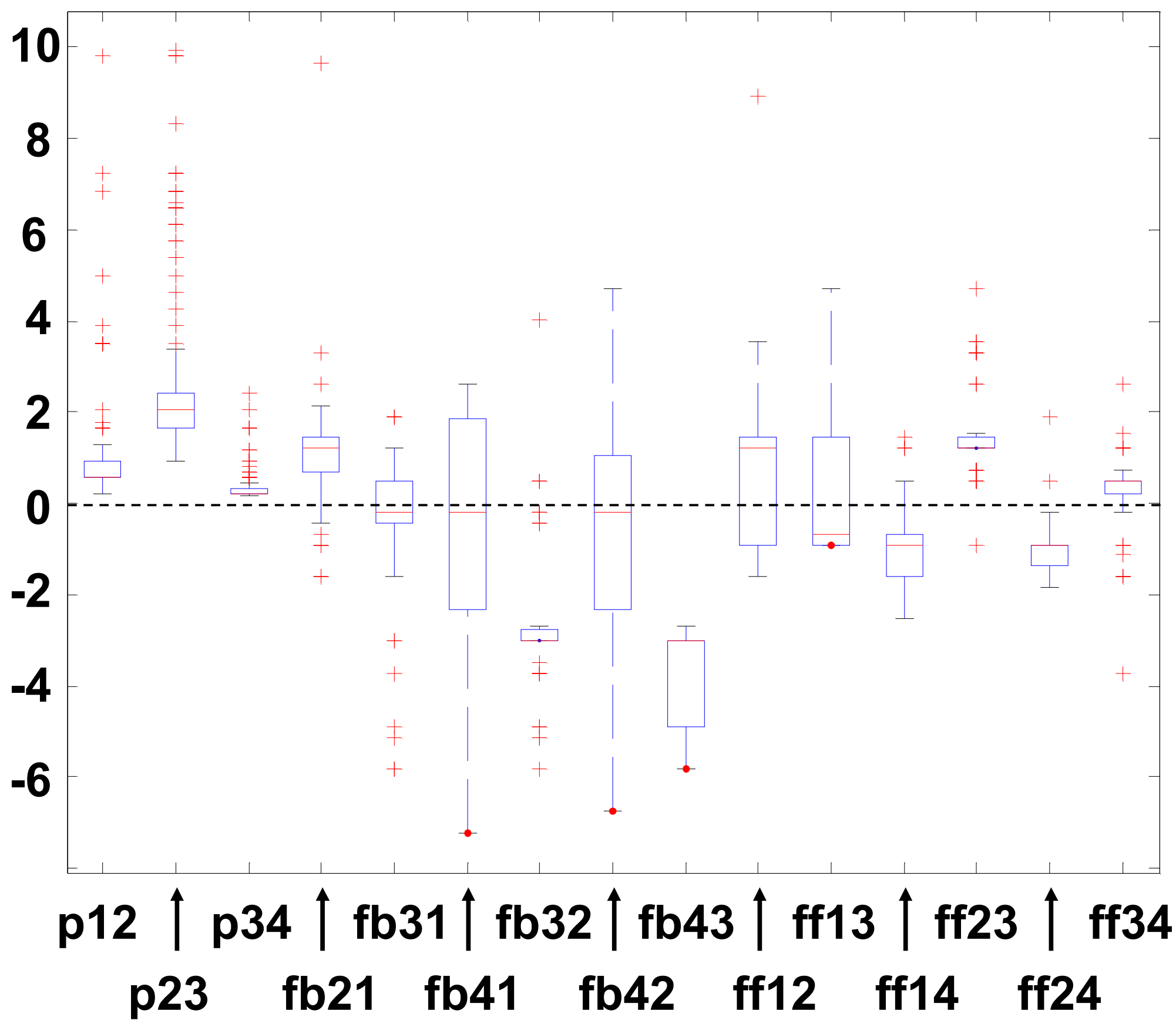

Supplementary Figure 21. Box Plot of parameter magnitudes for each model topology. The red, middle line is the median for all of the parameter values, and the upper and lower box boundaries are the upper and lower quartiles, respectively. The whiskers show the extent of the rest of the data, and the red points are the outliers. It can be seen that certain parameters have a small spread, indicating that they retain the same magnitude even in the presence of one or two additional regulatory loops. The units of the parameter values expressed on the ordinate are 1/day. This figure is a part of Fig. 3 of the main text.

1.4 Development of Scaled and Permuted Data Sets

In the analysis above we have considered the time course of erythroid development based on the approximation of cell surface expression over developmental time. Inherent in the approximation is some uncertainty in time measured in units of embryonic days, since each time point corresponded to a separate embryo and absolute measurement of developmental time is frequently impossible. To study the sensitivity of results to uncertainty in time measurement, we constructed hypothetical data sets (based on the set in Section 1) that were scaled randomly along the time axis, and data sets containing permutations of certain data points. More specifically, considering the current resolution in time to be 0.5 days, we added an uncertainty of up to +/- 0.4 days, and repeated the simulations. Moreover, to ensure accuracy in the selection of the points, certain points were removed, and the results were simulated again. The results are detailed in this section.

1.4.1 Randomly distributed time points

Considering the resolution in time for the original data set to be 0.5 days, in each of the derived data sets, we varied the time coordinate for points E13.0-E15.0 randomly between +/- 0.2 days and +/- 0.4 days of the original coordinates. The resultant trajectories vary in slope and magnitude over the experimental time course. We then analyzed the error in the metric *R* profiles in response to these dataset perturbations.

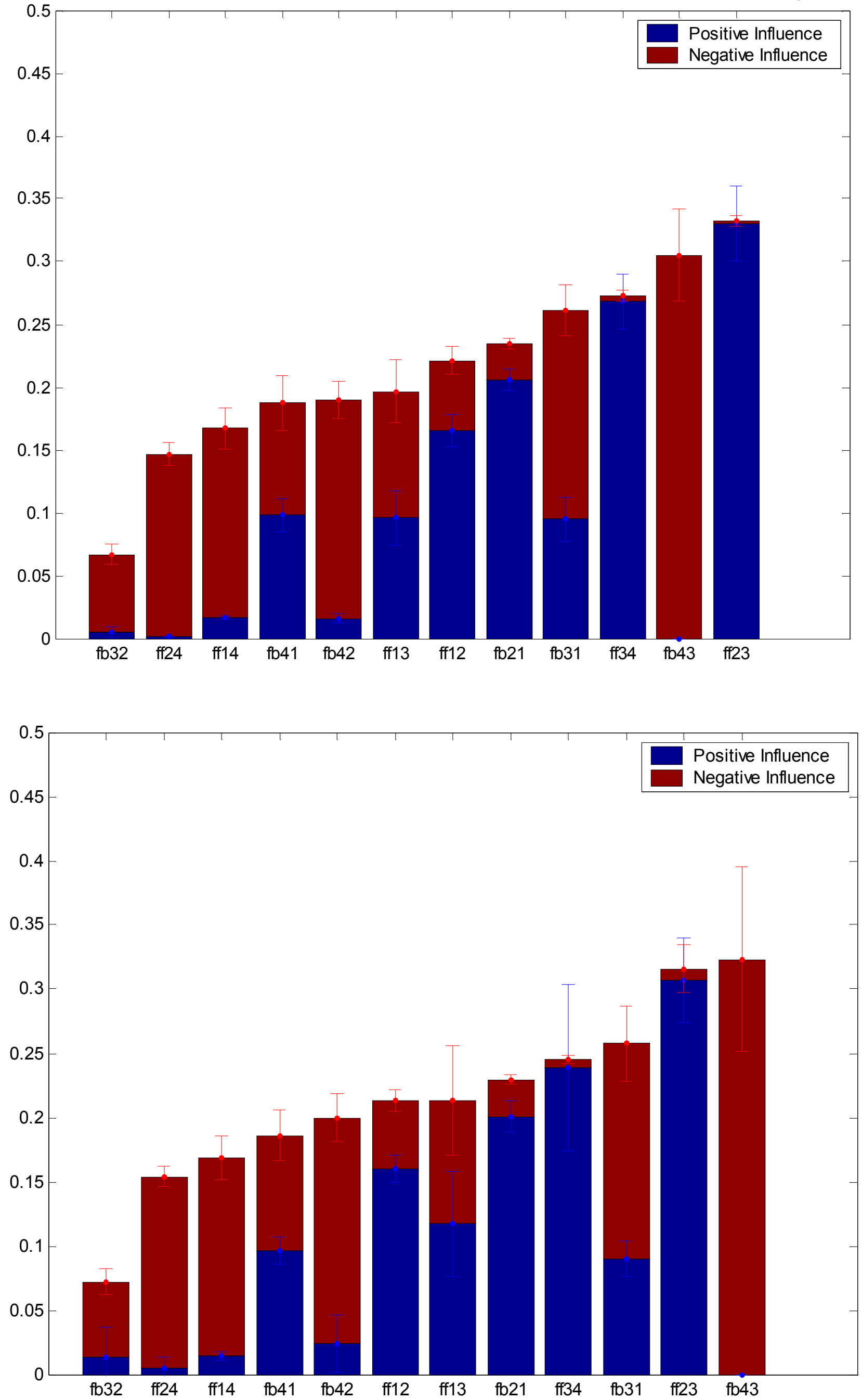

Figure 22. Ranking of interactions according to the *R* metric in randomized measurement time simulations. The time coordinates of the experimental data were varied randomly +/- a) <0.2 days and b) <0.4 days. Error bars represent standard deviation of the fractions of positive and negative parameter valies calculated for 3 independent analyses of the type shown in Fig. 19. The means are obtained by averaging across the 3 indpendent analyses.

The results show that the metric *R* profiles display little sensitivity to this variation. Therefore it is likely that although some properties of the distribution of *R* may alter, the relative rankings of ff23, ff34, fb43 (all high) and fb32 (low) are not sensitive to this data set perturbation.

1.4.2 Removing E14.5, 3 days to full differentiation

Because cells collected from E14.0 and E14.5 embryos exhibit very similar expression levels, we studied the possibility that they might have been collected from the embryos of the same stage, and therefore removed E14.5, while keeping all other time points the same (a separate simulation was performed where each of the time points that occurred later where presumed to occur 0.5 days earlier). The results of a simulation (Supplementary Fig. 23) suggest that the removal of E14.5

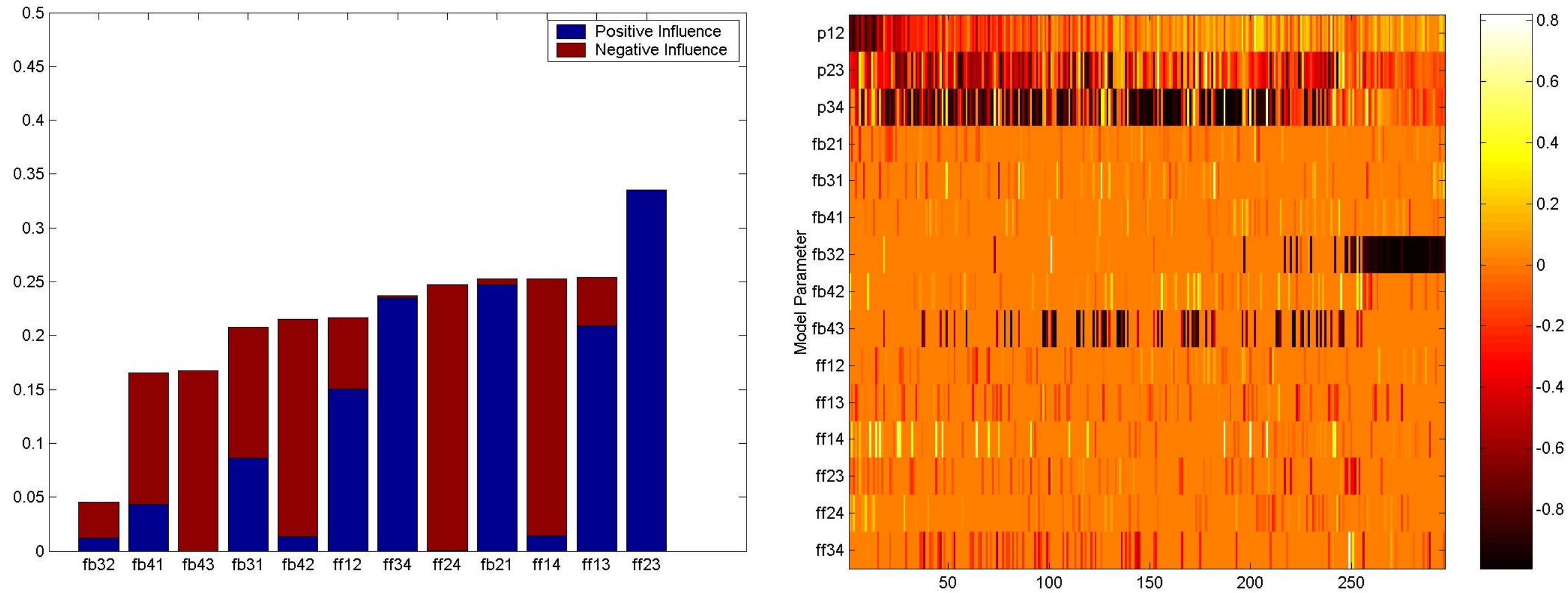

Supplementary Figure 23. Ranking of interactions according to the S metric and the corresponding control matrix with E14.5 removed, full differentiation in 3 days.

data (along with retaining the time coordinates of the other data points) leads to several effects. First, the robustness metric values for the bulk of the interactions (except ff23 and fb32) now are nor as spread as before, essentially clustering around 0.2. Second, there is a significant change in relative values of *S* in the intermediate robustness value range leading to changing of ranks, not surprising in view of the aforementioned little robustness value distribution. Thus ff34 and fb43 are now ranked as less significant. Importantly, however, ff23 is again ranked as the most significant with the robustness metric 0.3, further suggesting the importance of this interaction. The control diagram also reveals that ff34 and fb43 lose their control properties in highly ranked models, but ff23 retains a significant amount of control.

1.4.3 Removing E14.5, 2.5 days to full differentiation

In order to complement the analysis in section 4.2, where E15.0 and E15.5 were kept constant with respect to time, we also assumed that the entire interval, the differentiation terminating with Ter119^{HIGH}, CD71^{LOW} occurs over 2.5 days, as opposed to 3.0 days. The results show that although now ff23 is still ranked topmost in terms of its significance, its robustness metric value, 0.3, is more closely matched by many other possible interaction mechanisms, so that the overall spread of robustness metric values (except fb32 and ff24) is very narrow, making the ranking results less reliable. Nevertheless, both ff23 and ff34 are ranked at the top again.

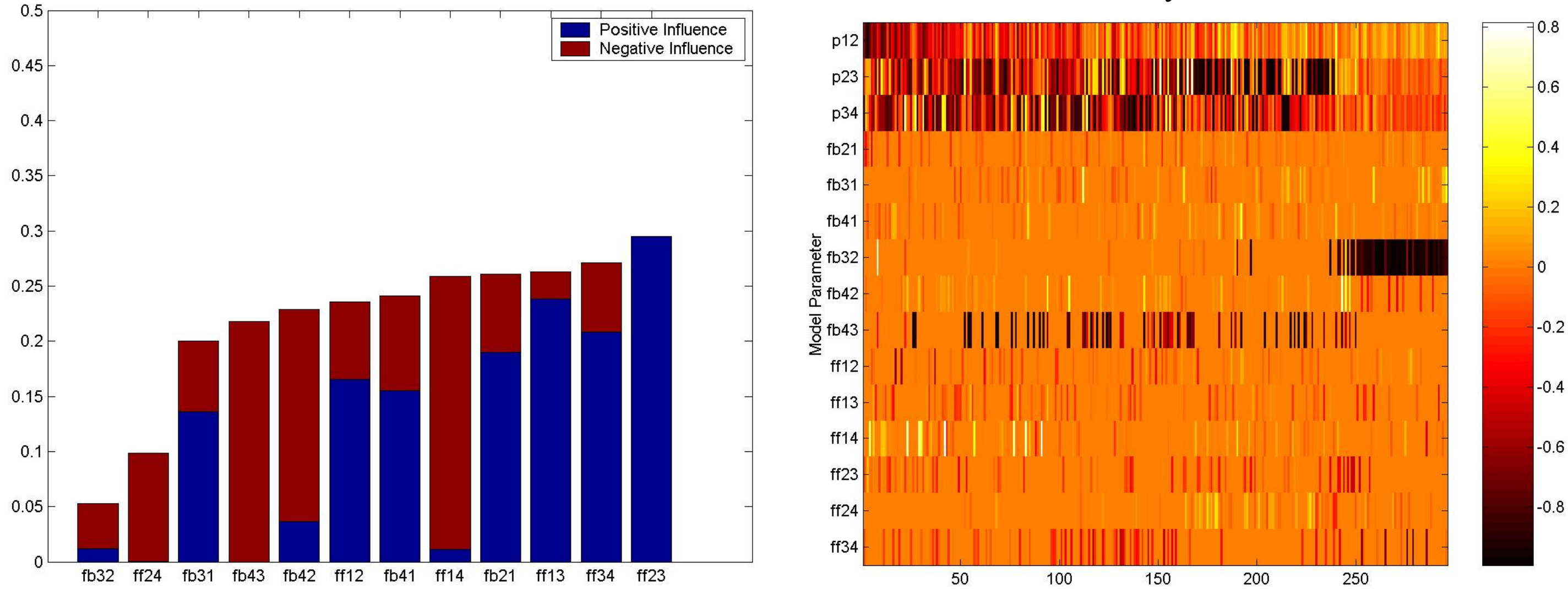

Supplementary Figure 24. Ranking of interactions according to the R metric and the corresponding control matrix with E14.5 removed, full differentiation in 2.5 days

1.4.4 Removing E13.5, 2.5 days to full differentiation

As an additional perturbation we have removed the dataset for another intermediate day, E13.5, again compressing the whole time course to 2.5 days. Again, we observed that ff23 and ff34 have retained their high ranks, whereas the significance of fb43 decreased.

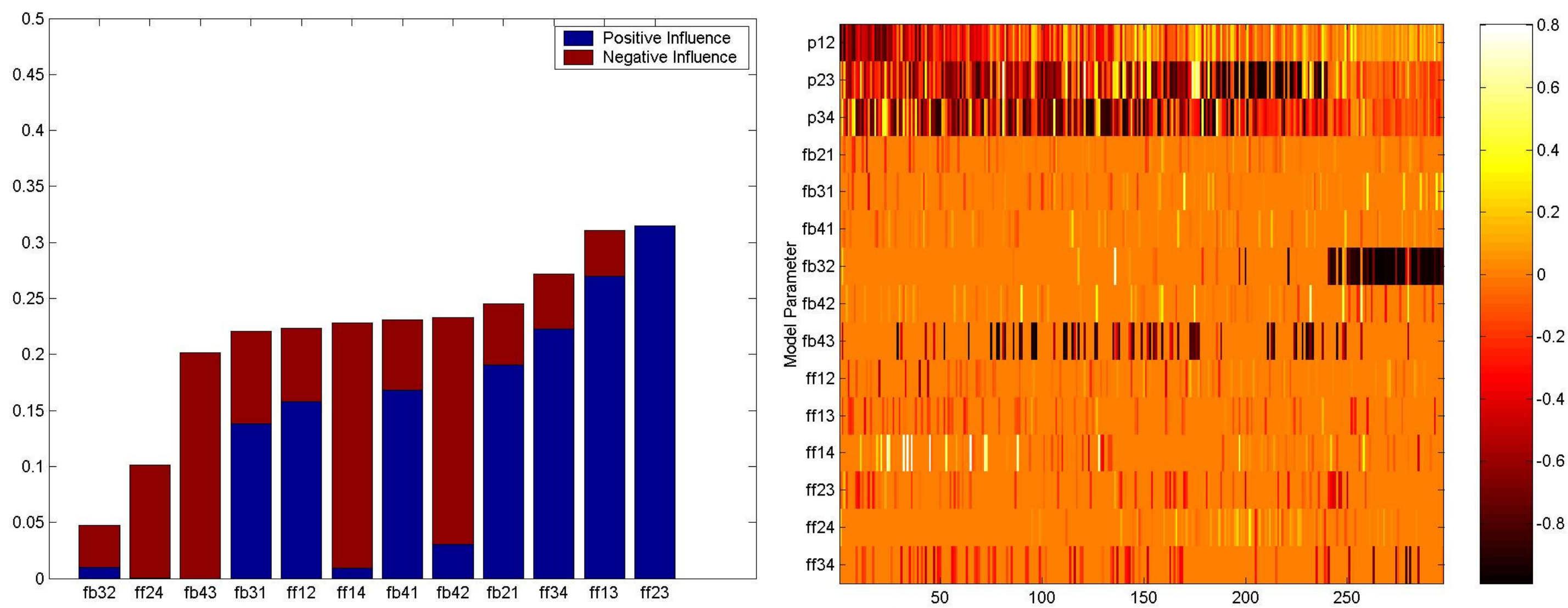

Supplementary Figure 25. Ranking of interactions according to the *R* metric and the corresponding control matrix with E12.5 removed, full differentiation in 2.5 days

In summary, removal of time points corresponding to intermediate embryonic days does not affect the prediction of the importance of ff34 and, especially, ff23, interactions, while reducing the significance of fb43.

1.5. Testing Computational Methods

We also studied the sensitivity of the results to exact computational methods of determining the predominant model topologies. In alternative analyses, we studied suitability of relying on particular criteria for model selection. In the algorithm we used, data on fractional cell numbers in all four states were used in fitting a model to the experimental data and estimation of residuals of the fitted model and 200 corresponding model perturbations. However, the precise gating of the first and last stages is subject to more uncertainty compared to the stages 2 and 3, based on the magnitude of ratios of cells in these stages in each of the experimental data points. The first stage and the last stage primarily occur in the first and last time point measurements respectively, and as the proportions of cells in these states tend to be low, there is a greater possibility of error, e.g., due to minor changes in gating. Therefore, as an alternative method, we tested the role of increasing reliance in treating regression in some cases, and sensitivity in others, on the middle two stages, which appear predominantly throughout the course of embryonic development. That is to say, we tested using only SSR values from the intermediate stages to determine a set of parameters, and/or using only the intermediate stages in calculating the sensitivity. The results are shown in Supplementary Figs. 26-29.

2 State Regression, 2 State Sensitivity Analysis

Due to propagation of the erythropoietic wave, comparatively less data exist on the dynamics of cell numbers in states 1 and 4. We thus tested if the results would be affected if the analysis relied primarily on states 2 and 3. As the system is conserved, every parameter is represented even in this reduced form, as the differentiation equations for stages 2 and 3 contain all transitions, h12, h23, and h34 (Equations 1 and 2). Therefore, parameter optimization still reflects the first and last stage, but is weighted much less in their favor. As can be seen in Supplementary Fig. 26, the two predominant mechanisms become even more dominant, and are highly favored as the regulatory connections in this system.

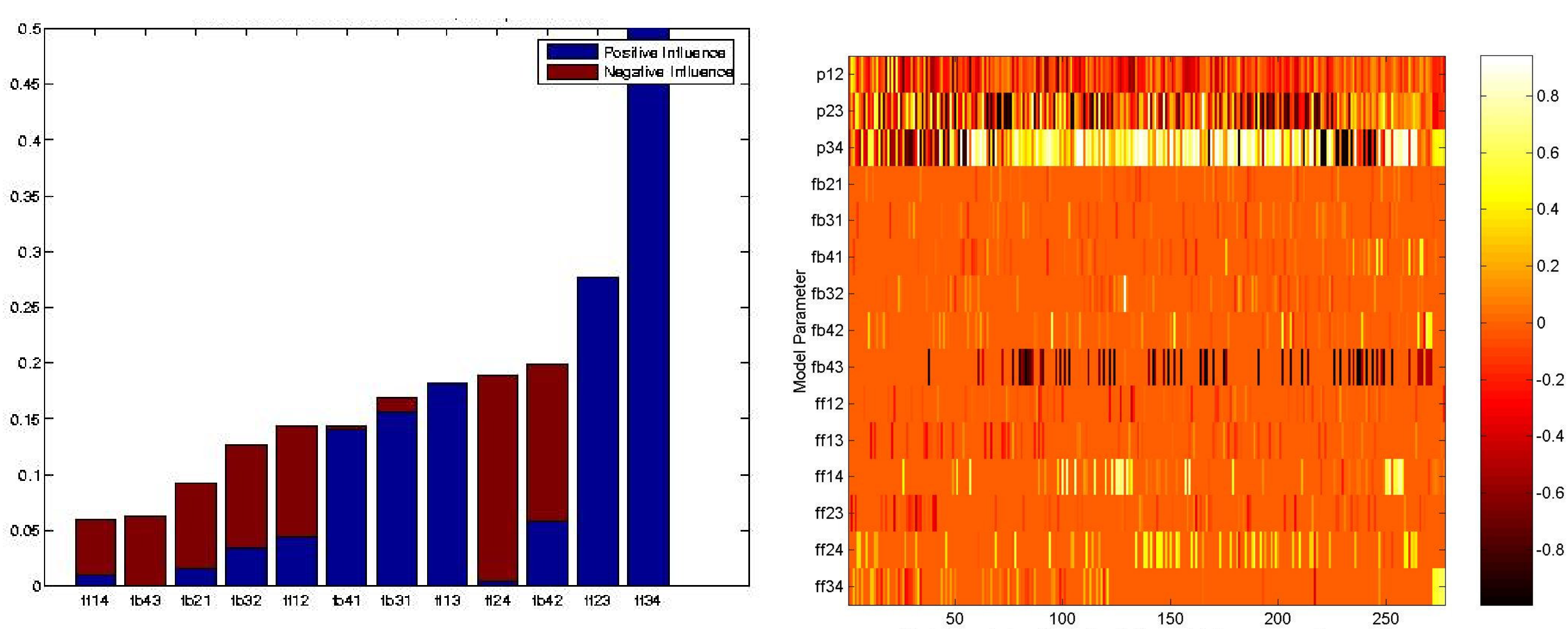

Supplementary Figure 26. Ranking of interactions according to the *R* metric and the corresponding control matrix with states 1 and 4 removed in the fitting procedure

2 State Regression, 2 State sensitivity, 50% parameter variation

We initially chose to test only local perturbations of parameter values (5%). To test if the previous results hold at very large perturbation we ran a simulation where we used a 50% parameter variation. Supplementary Fig. 27 shows that even in this case, ff23, ff34 and fb43 are top ranked.

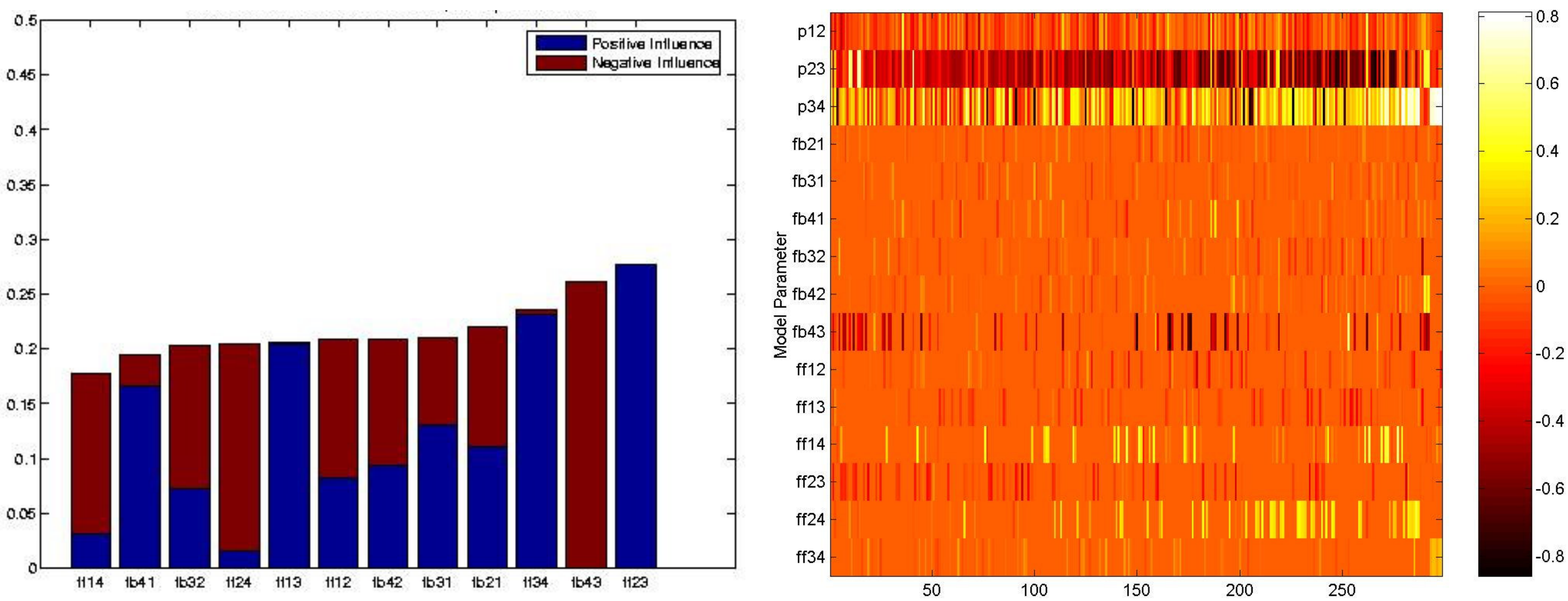

Supplementary Figure 27. Same as in Supp. Fig. 26, but 50% variation of parameters in multi-variate sensitivity analysis

2 State Regression, 2 State Sensitivity, similarity to the fitted data

In the preceding analysis, in parameter variation studies, we compared each generated time course to the experimental data given in Table 1. It is of interest to see if the results would change, if the comparison was instead performed with respect to the curves resulting from the fitting procedure for each model. The results are shown in Supplementary Fig. 28. The ff23 term remains ranked at the top, but the rest of the results are significantly less differentiated. We note that this analysis does not reflect the experimental data to the same degree as the analysis above, since we measure the similarity during the sensitivity analysis between the solutions resulting from the perturbed parameter set and the original fit, so the similarity to the experimental data is confined to the fitted set. The parameters that appear robust in this analysis may not actually endow the system with a good fit.

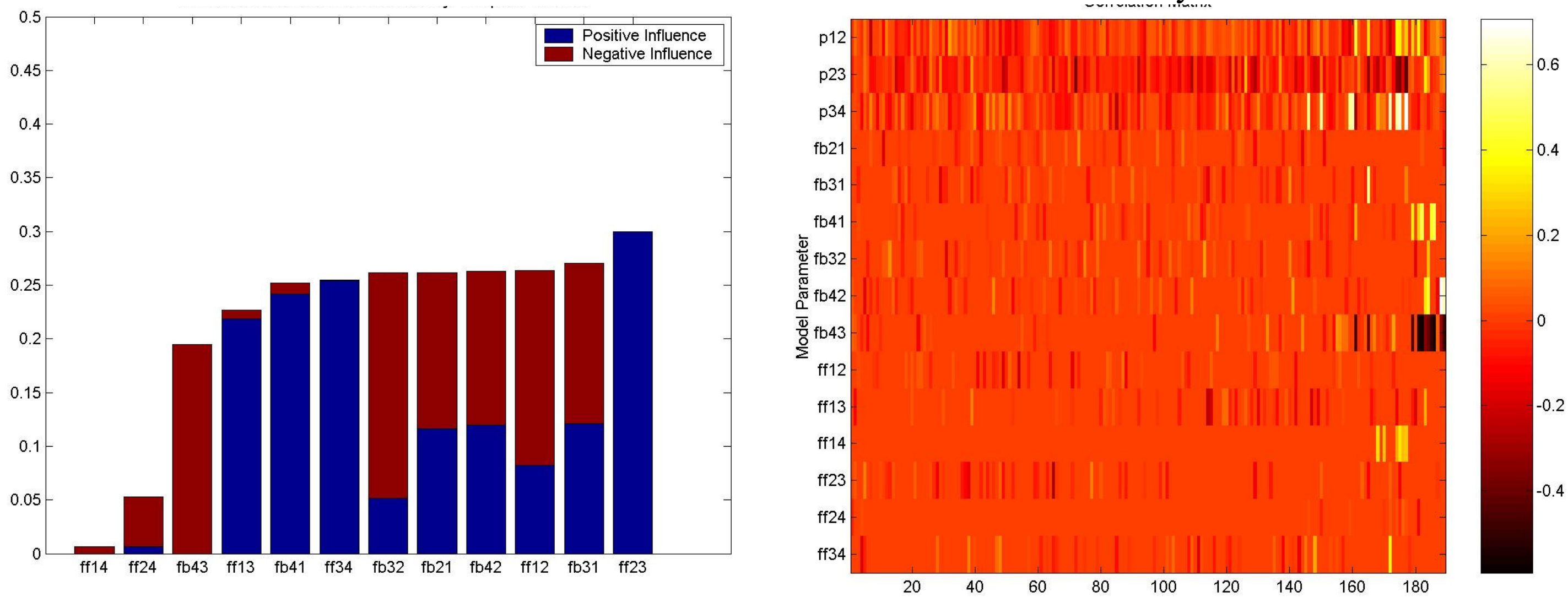

Supplementary Figure 28. Ranking of interactions according to the *R* metric and the corresponding control matrix with residuals calculated with respect to the fitted curves in the multi-variate sensitivity studies, based on states 2 and 3 only

4 State Regression, 4 State Sensitivity, similarity to the fitted data

Finally, we wanted to see if the results shown in Supplementary Fig. 29 would change if all 4 states rather than 2 states were used in fitting and parameter variation. As can be seen in Supplementary Fig. 28, ff23 is still top ranked and fb43 is the second top ranked parameter, although the value of *S* for fb43 is similar to those of most other parameters.

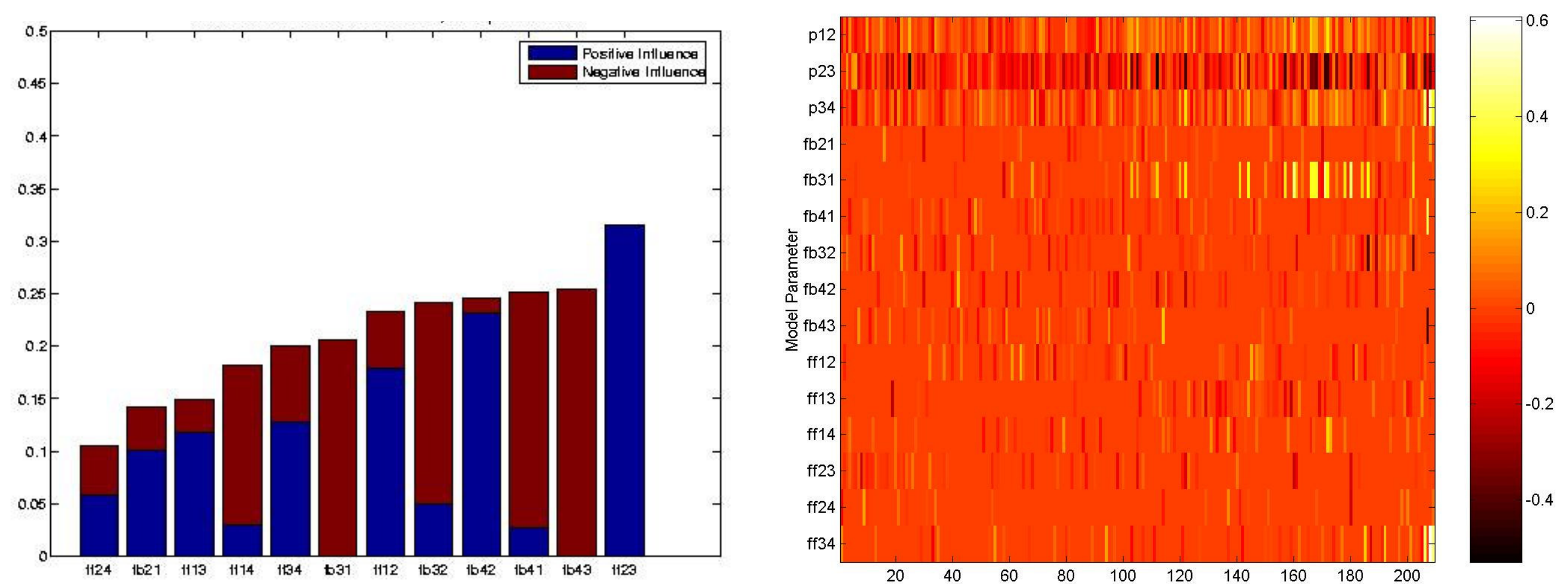

Supplementary Figure 29. Same as in Suppl. Fig. 28, but 4 states are used in fitting and multi-variate sensitivity analysis

1.6. Relative rankings of models with ff23 and ff34 interactions

As the main predictions of the model were that ff23 and ff34 would be likely to be found in any model describing fetal erythropoiesis, we wanted to see how the models containing these two interactiosn would rank compared to other models. However, we need to stress that the compatibility of any two interactions (including ff23 and ff34) is not in and of itself a function of the algorithm's performance. Each interaction participates in an identical number of models for each given model complexity (i.e. models with one, two or three interactions). Of the 298 model topologies we tested, the ff23 and ff34 interactions co-exist in one model where there are only two interactions, and in 10 models in which there are 3 interactions. As can be seen in the Supplementary Figure 30 we find that these models are indeed ranked among the best models in terms of both their fitness and robustness. We also confirmed through Wicoxon non-parameteric test that both the fitness and robustness of models with ff23,ff34 interactions are significantly better ($p<0.001$) than those in the rest of the model population.

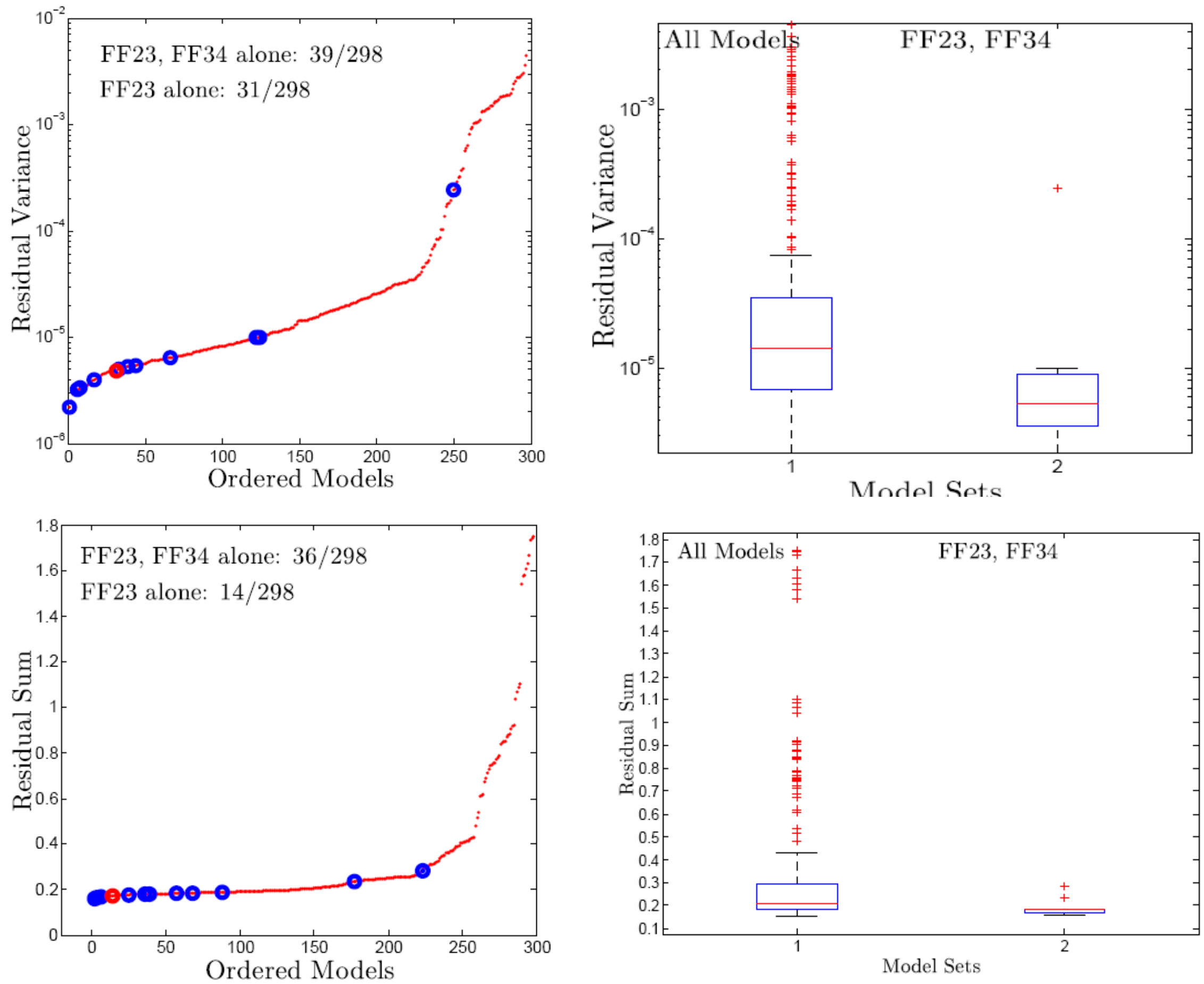

Supplementary Figure 30. Ranking of models by the mean and variance of the SSR (left column, SSR is equivalent to 'Residual Sum'), and box plots contrasting the distributions of SSR means and variances for the entire model population and the subset of models containing both ff23 and ff34 (right column). The blue circles indicate models containing both ff23 and ff34, whereas the red circles indicate the model containing ff23 only. The rankings of the two models with just ff23 or just ff23-ff34 are indicated. The box indicates the inner quartile range. The median is the horizontal red line within the box. The whiskers extend to cover the upper and lower quartiles up to a distance of 1.5 times the inner quartile range. Red crosses indicate extreme values (outliers beyond the whisker length).

1.7. Interpretation of prominence of inter-state interactions

It is clear from the Supplementary Figs. 19-29 that the analysis of both the original and modified (control) data sets consistently yields similar results in terms of the parameters deemed significant for maintaining robust fit of a model to experimental data. We hypothesized that cell-cell interactions represented by these parameters might exist in erythopoiesis. In particular, three interactions stand out as ranked at the top with the highest significance across the control simulations. These are the positive feedforward interactions ff23 and ff34 and the negative feedback interactions fb43. The first two interactions represent (see Supplementary Table 3) increased rates of transition out of the states 2 and 3 in proportion to the squares of the numbers of cells in 2 and 3 respectively. Thus these mechanisms are autoregulatory in the sense that transition out of a particular state depends only on the population in that stage; both the source and target for regulation are the same. Since both ff23 and ff34 are predicted to be positive, their effect is to decrease the number of cells in states 2 and 3, in proportion to the relative numbers of cells in each of the states, indicating a clear possibility that either an increase of the number of cells in these states increases the rates of differentiation out of these states or the rates of apoptosis in these states. In the latter case, some signal must exist that would allow the cells in either state 2 or state 3 to act in autocrine or paracrine fashion on the cells in the same state leading to apoptotic death. We hypothesized that that this autocrine and paracrine pro-apoptotic mechanism indeed is responsible for the observed behavior, focusing on Fas and FasL interactions as potential candidates. As described in the text, the experimental results provide support for this hypothesis.

The negative feedback interaction fb43 corresponds to an effective decrease of rate of transition between states 3 and 4, in proportion to the product of the relative numbers of cells in these states. This feedback can be interpreted as either upregulation of the number of cells in the state 3 due to a decrease rate of either differentiation or apoptosis from this state (both as a function of the number of cells in the state 4), or as an increased cell death in the state 4, in proportion to the number of cells in the state 3. The latter interpretation is a consequence of operating with normalized cell numbers and thus conservation assumption made for the model, and of the absence of a transition out of the state 4. Thus, if the cells in the state 4 undergo apoptosis, the effect will be in apparently less efficient transition from 3 to 4, which is the source of populating the state 4. The data provided in the main text agree with this latter interpretation, although the former ones cannot be excluded.

Interestingly, the parameters responsible for the interactions ff23, ff34 and fb43 are either predicted to be strongly positive (the first two) or negative (the last) unlike many other parameters, whose sign may vary with the actual model (Supplementary Fig. 21). In addition, ff23, ff34 and fb43 are predicted to exercise a substantial degree of control of the fit as predicted by the fact that they occur with high frequency in highly robust models and that the values of the control metric for the corresponding parameters in highly robust models are relatively high (Supplementary Fig. 20 and the right panels in Supplementary Figs. 23-29). These observations further support potential importance of these interactions and argues for their presence in the real erythropoietic system. Below we provide the proposed scheme of interactions in the real

erythropoiesis developmental regulatory network. In addition, to including interactions ff23, ff34 and fb43, we have assigned different thickness to unmodulated transition rates represented by the parameters p12, p23, p34, as determined from Supplementary Fig. 21.

Table 3. State dependency for each potential model parameter.

Connection	Sign	Effect on Transition	Effect on States	State dependency
fb21 (p4)	Positive<br>Negative	Increase h12<br>Reduce h12	Reduce 1, Increase 2<br>Increase 1, Reduce 2	x_1x_2
fb31 (p5)	Positive<br>Negative	Increase h12<br>Reduce h12	Reduce 1, Increase 2<br>Increase 1, Reduce 2	x_1x_3
fb41 (p6)	Positive<br>Negative	Increase h12<br>Reduce h12	Reduce 1, Increase 2<br>Increase 1, Reduce 2	x_1x_4
fb32 (p7)	Positive<br>Negative	Increase h23<br>Reduce h23	Reduce 2, Increase 3<br>Increase 2, Reduce 3	x_2x_3
fb42 (p8)	Positive<br>Negative	Increase h23<br>Reduce h23	Reduce 2, Increase 3<br>Increase 2, Reduce 3	x_2x_4
fb43 (p9)	Positive<br>Negative	Increase h34<br>Reduce h34	Reduce 3, Increase 4<br>Increase 3, Reduce 4	x_4x_3
ff12 (p10)	Positive<br>Negative	Increase h12<br>Reduce h12	Reduce 1, Increase 2<br>Increase 1, Reduce 2	x_1^2
ff13 (p11)	Positive<br>Negative	Increase h23<br>Reduce h23	Reduce 2, Increase 3<br>Increase 2, Reduce 3	x_1x_2
ff14 (p12)	Positive<br>Negative	Increase h34<br>Reduce h34	Reduce 3, Increase 4<br>Increase 3, Reduce 4	x_1x_3
ff23 (p13)	Positive<br>Negative	Increase h23<br>Reduce h23	Reduce 2, Increase 3<br>Increase 2, Reduce 3	x_2^2
ff24 (p14)	Positive<br>Negative	Increase h34<br>Reduce h34	Reduce 3, Increase 4<br>Increase 3, Reduce 4	x_2x_3
ff34 (p15)	Positive<br>Negative	Increase h34<br>Reduce h34	Reduce 3, Increase 4<br>Increase 3, Reduce 4	x_3^2

Supplementary References

1. M. Socolovsky *et al.*, *Blood* **98**, 3261-73. (2001).
2. S. A. Kauffman, *The origins of order* (Oxford University Press, New York, 1993).
3. T. S. Gardner, D. di Bernardo, D. Lorenz, J. J. Collins, *Science* **301**, 102-105 (July 4, 2003, 2003).
4. C. D. Perttunen, Jones, D.R., Stuckman, B.E., *Journal of Optimization Theory and Application* **79**, 157-181 (1993).
