# Supplementary material for: Negative Autoregulation by FAS Mediates Robust Fetal Erythropoiesis
Source: PLoS Biol. 2007 Sep 25;5(10):e252. doi: 10.1371/journal.pbio.0050252 (PMC1988857; doi:10.1371/journal.pbio.0050252)
Supplement: Supplementary file 2 [file pbio.0050252.sd002.pdf]

Protocol S2: **Autoregulatory Fas-FasL interaction in erythroid development: supplementary material and figures**

Section 2.1: Absolute numbers of fetal liver cells during E11.5 to E15.5 page 2

Section 2.2: The erythroblastic island on E15.5 page 3

Section 2.3: Annexin V binding in individual embryos E11.5-E15.5 page 5

Section 2.4: The significance of x_2 values in E11.5 embryos page 6

Section 2.5: The contribution of Fas-mediated apoptosis to the decay in x_2. page 8

Section 2.6: Mixing of states 1, 2 , 3 and 4 *in vitro* page 11

Section 2.1: Absolute number of fetal liver cells during E11.5 to E15.5

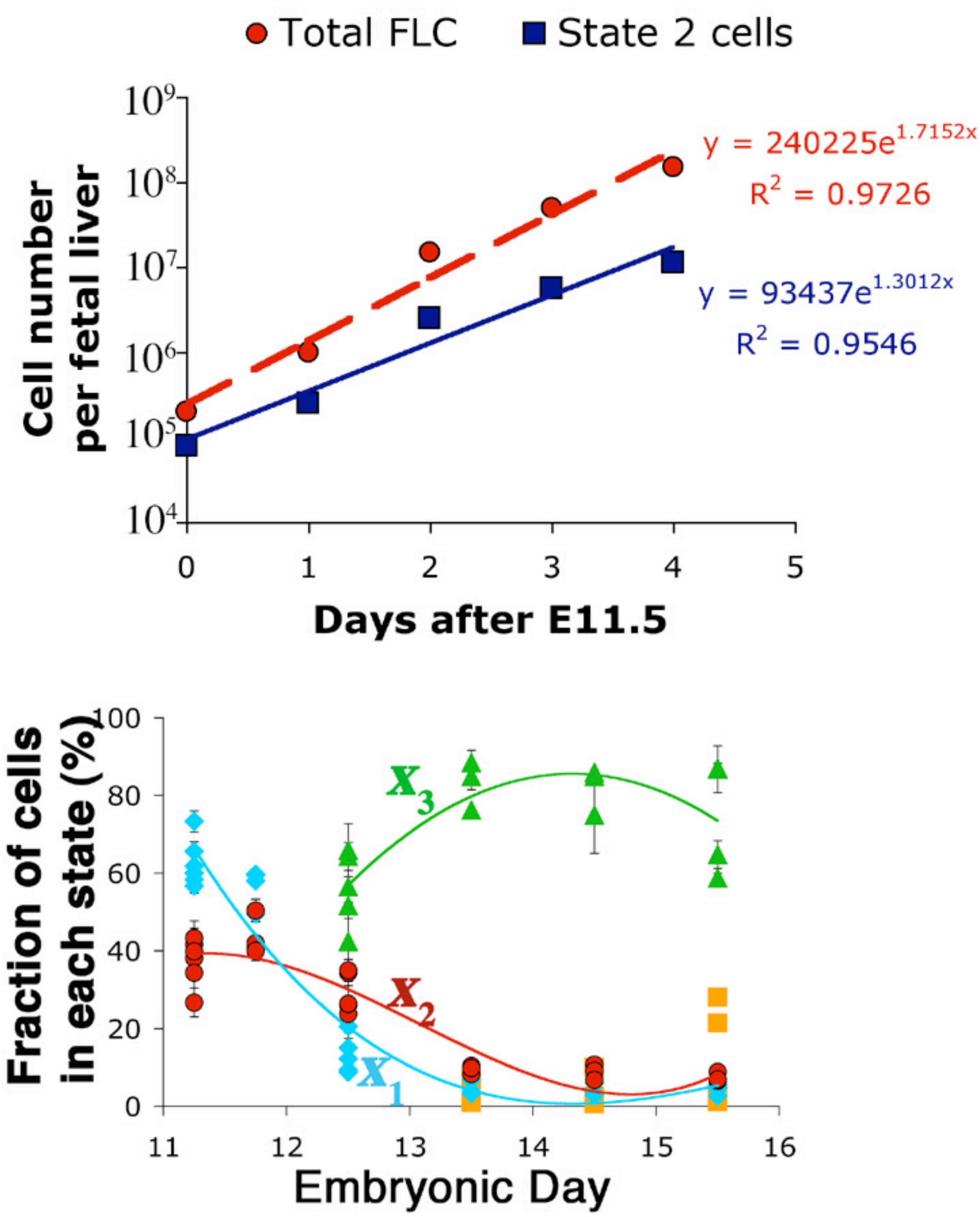

The upper panel shows exponential growth in the absolute number of cells in fetal liver between E11.5 and E15.5. Cells in each liver were counted on each day of development (red circles). The corresponding number of cells in state 2 were calculated using the mean value of x_2 for each day, presented in Fig 4A of the main text and shown here in the lower panel.

Section 2.2: The erythroblastic island on E15.5

The data below shows that erythroblastic islands on E15.5 contain rings of erythroblasts at a synchronous differentiation stage. This supports the conclusion arrived at in the main text, that erythroblasts traverse state 2 as synchronous cohorts, with little free mixing between cells.

A

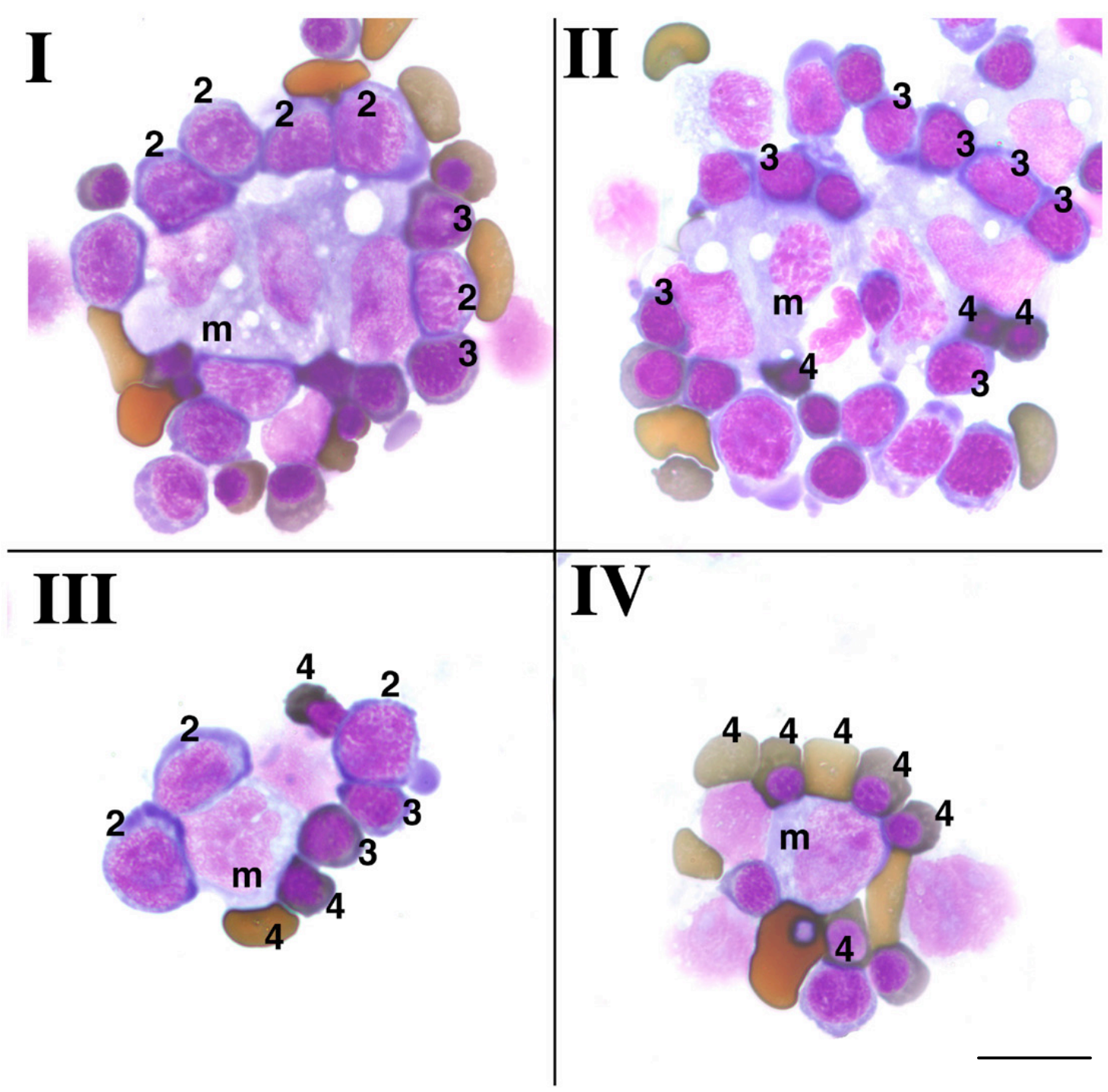

B

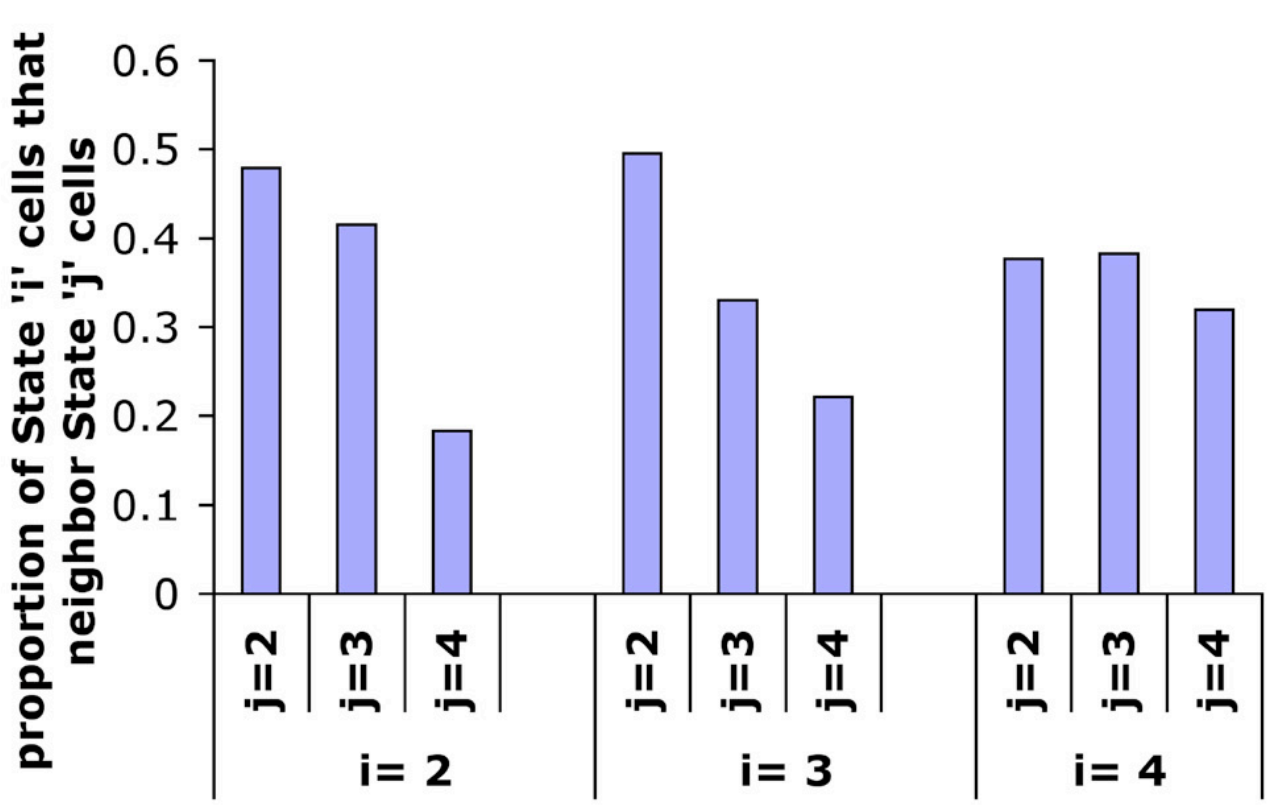

A. Erythroblastic islands isolated from E15.5 fetal liver show the majority of erythroblasts are in rings of synchronous development where they are neighboring cells of the same

maturation stage, especially in panels I, II and III. A smaller fraction of erythroblasts neighbor cells of heterogeneous maturation. m=central macrophage. Arabic numerals indicate the likely developmental state of erythroblasts. Scale Bar: 20μ.

B Quantitation of neighboring cells in E15.5 erythroblastic islands: Erythroblastic islands from E15.5 fetal liver were examined in cytospin preparations. The immediate neighbors of each of 839 cells in 94 erythroblastic islands in 25 consecutive fields were scored (each cell had between 1 and 3 interactions). The likely state of each cell was determined morphologically, as in **A**. This data shows that even on E15.5, when state 2 cells constitute less than 10% of all fetal liver, they frequently (≈50% of cells) neighbor other state 2 cells.

Section 2.3: Annexin V binding in individual embryos E11.5-E15.5

A The fraction of cells in state 2 that are Annexin V+ (Av^{+}_{2}) in the period E11.5-E15.5. Same dataset as in Fig 4B, but each data point represents an individual embryo (Fig 4B shows means for each litter of embryos).

B,C Analysis of the same embryos (symbols as for '**A**'), correlating Av^{+}_{2} with x_2 (also presented in Fig 6B,C):

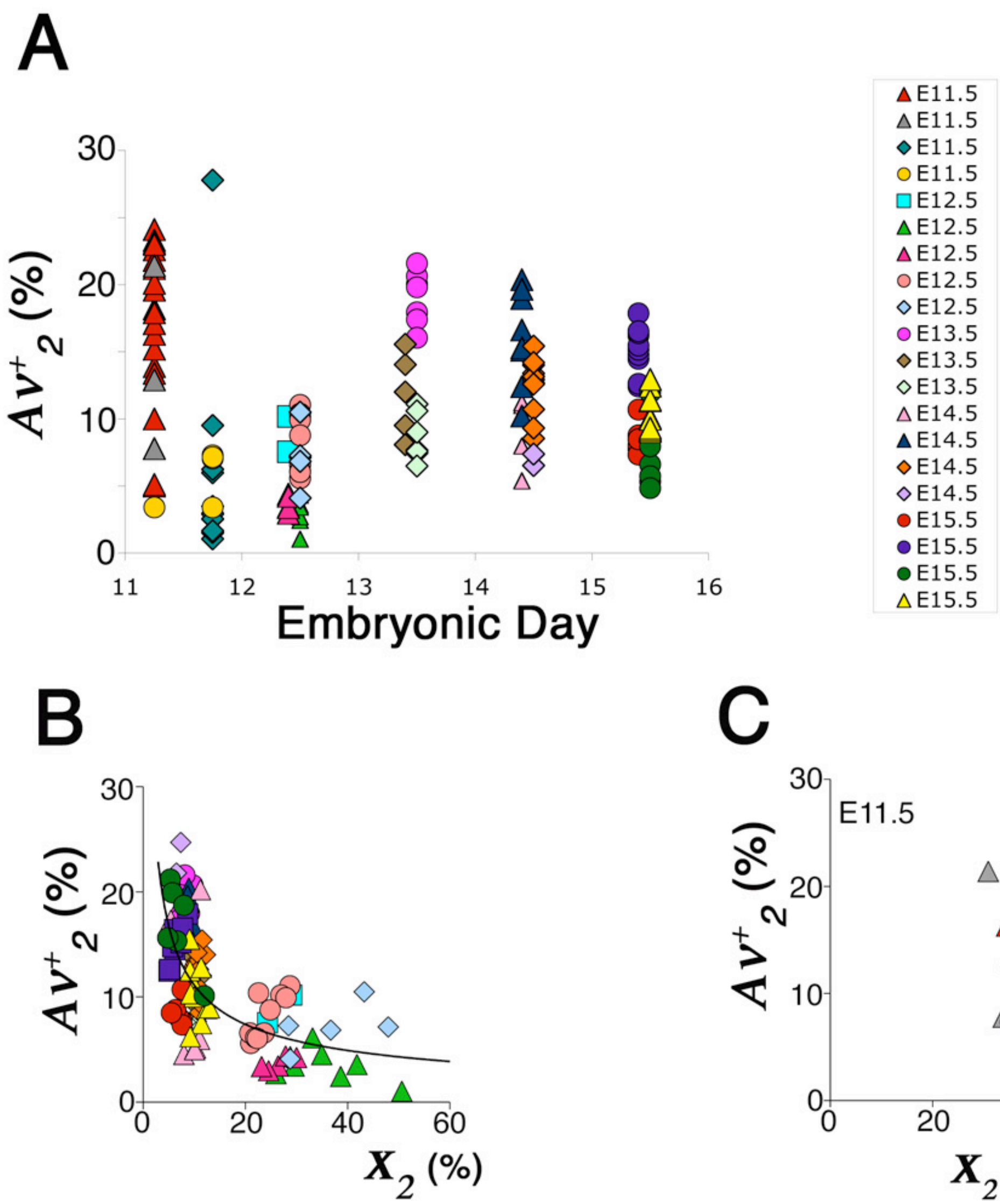

Section 2.4: The significance of x_2 values in E11.5 embryos

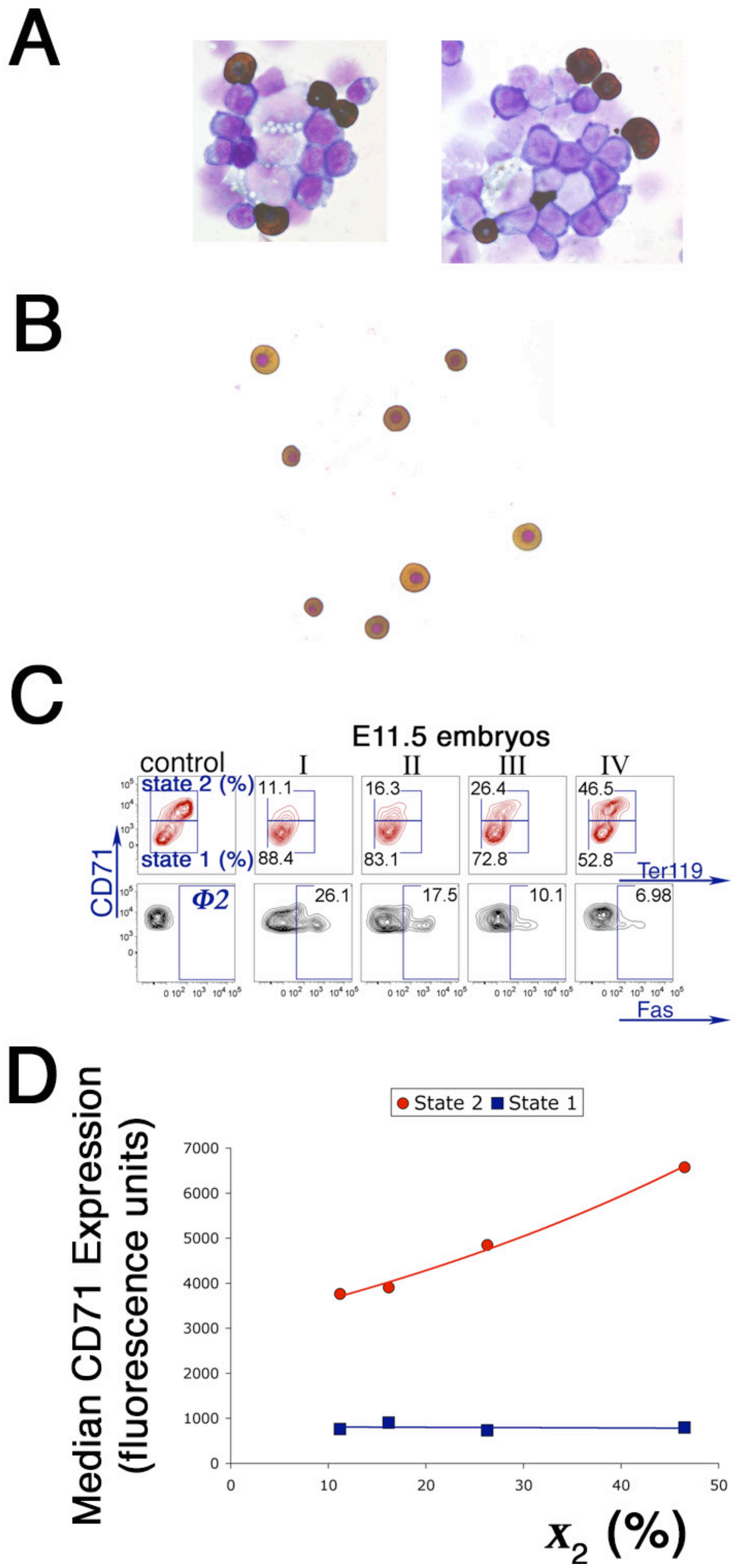

A,B E11.5 contain cells only in states 1 and 2

Erythroblastic islands on E11.5 (panel '**A**') do not contain cells that resemble state 3 cells (see section 2.2 for comparison). Nevertheless, we occasionally found E11.5 fetal liver samples containing a variable number of CD71^{high}/Ter119^{high} cells. We sorted these using flow-cytometry, and cytospin preparations (panel '**B**') show them to contain exclusively yolk-sac (nucleated, or primitive lineage) red cells. These yolk sac erythrocytes are hemoglobinized (brown) and quite distinct in morphology from state 3 cells, the latter being basophilic (blue cytoplasm) and containing a large nucleus with relatively open chromatin structure (see section 2.2). The yolk sac erythrocytes are also visible as the hemoglobinized (brown) cells associated with the erythroblastic islands in panel '**A**'. They may, in part, arise as contamination from blood introduced during dissection of the fetal liver, unavoidable at this developmental stage due to the very small size of the liver. This source of contamination becomes insignificant in older, larger embryos.

C,D The variable values for x_2 found in E11.5 embryos reflect the different extents to which the first cohort of erythroblasts has advanced within state 2.

Four embryos from the same litter are shown in panel '**C**', also shown in the main text, Fig 6D. They are arranged in order of increasing proportion of cells in state 2 (x_2). The median level of CD71 expression per cell in state 2 can be seen to increase in parallel with x_2 (panels '**C**', '**D**'), though always significantly above the median CD71 level of state 1 cells (panel '**D**'). This is in support of our assumption that x_2 reflects the extent to which the first cohort of cells has advanced within state 2: the increase in CD71 level is one of the various differentiation changes that are gradually taking place in the cells during state 2.

Section 2.5: The contribution of Fas-mediated apoptosis to the decay in x_2.

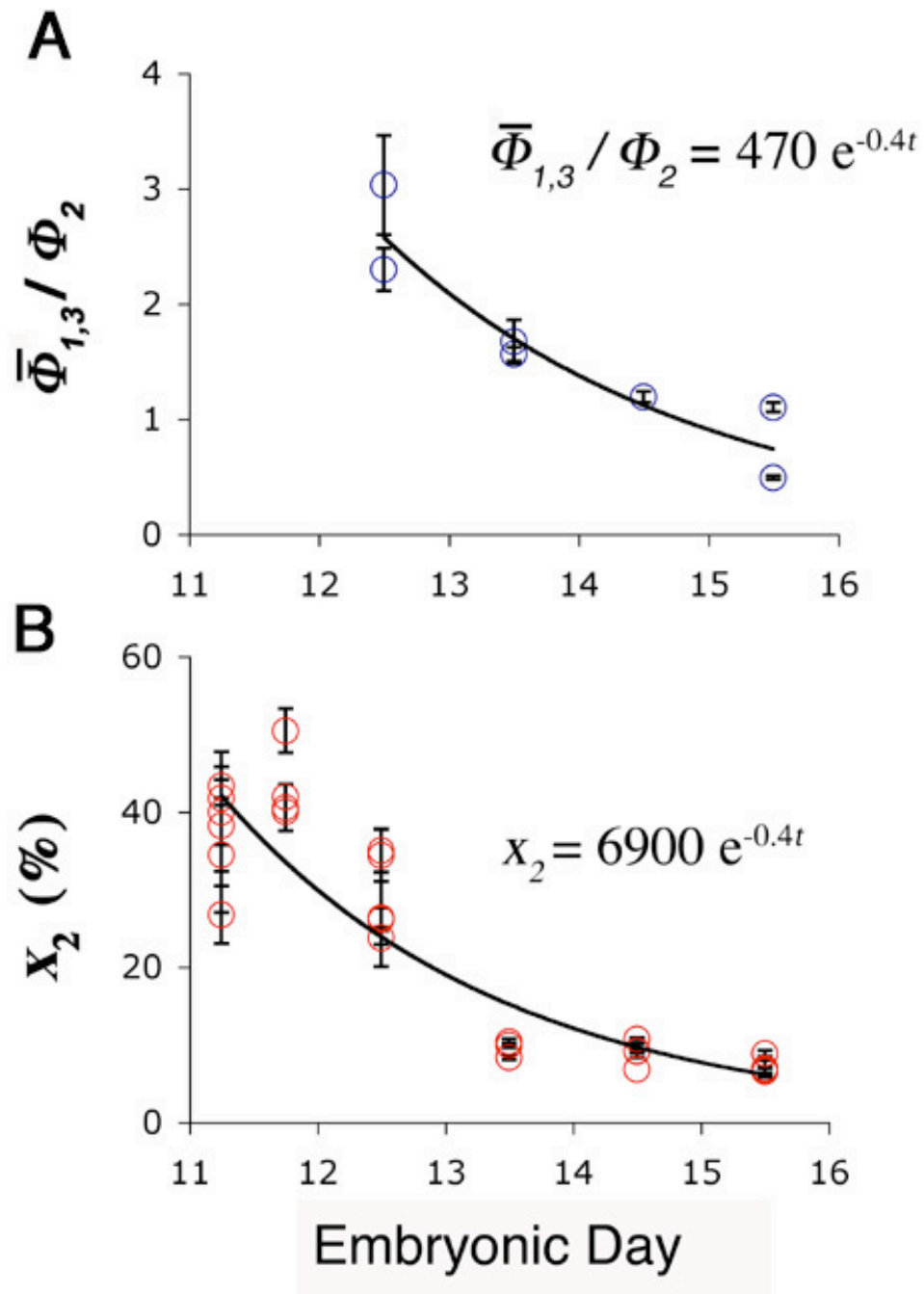

C

Embryonic Day	Expected	Observed	
E13.5	1.28	*1.28*	p<0.001
E14.5	1.65	*1.58*	p<0.001
E15.5	2.11	Not Done	

A, B Both x_2 and the ratio $\bar{\Phi}_{1,3} / \Phi_2$ decline with embryonic development at a similar rate, that can be fitted with an exponential curve containing a decay rate constant equal to 0.4. The equations describing the fitted exponential curves are shown (R^2=0.82 and 0.87 for '**A'** and '**B**', respectively). The similarity in these time-dependent decays suggests that $\bar{\Phi}_{1,3} / \Phi_2$, a measure of the rate of decline in Φ, is directly proportional to x_2 (also see main text). The proportionality between $\bar{\Phi}_{1,3} / \Phi_2$ and x_2 is another reflection of the inverse relationship between Φ_2 and x_2 (Fig 6A), since Φ_1 and Φ_3 remain relatively constant over this period.

Panel '**A'** contains the same dataset as Fig 6F, but each data point represents the mean value for a litter of embryos. Panel '**B**' shows the same data as in Fig 4A. The curve that fits the single-embryo data in Fig 6F is described by a very similar equation.

C The results presented in the main text strongly suggest that the rate of change in the fraction of cells in the state 2 could be approximated by the following equation:

$$\frac{dx_2}{dt} = \alpha x_1 - \beta x_2 - \gamma x_2^2 \qquad \text{(i)},$$

Here α describes the rate of appearance of cells in state 2 through differentiation of cells in state 1, β describes loss of cells in state 2 due to differentiation of cells in state 2 into cells in state 3 and γ describes the rate of loss of cells due to apoptotic negative auto-regulation. In this section, we will attempt to give an estimate of the number of cells in state 2 that could be potentially retained in the absence of apoptotic auto-regulation, namely, in *lpr* or *gld* embryos. A complete elimination of the apoptotic regulation term in (i) yields:

$$\left.\frac{dx_2}{dt}\right|_{No\ apoptosis} = \alpha x_1 - \beta x_2 \qquad \text{(ii)}$$

Although, to obtain the total increase in the fraction of cells in state 2 due to lack of apoptosis, one can integrate equations (i) and (ii) and compare these integrals, the results are not straightforward to analyze. Instead, we will make a few simplifying assumptions about the system and determine the approximate lower bound estimate on the desired quantity.

First we will focus on later stages of development, and thus assume that the contribution of the influx into state 2 through differentiation of cells in state 1 is negligible. This assumption is equivalent to putting the value of the parameter α in both equations to zero. Second, we can attempt to see if the solution to the equation (i) might be approximated by a simple exponential decrease. This would allow direct comparison of two exponential decay curves resulting from the simplified equations (i) and (ii). To see what error one might expect from this approximation, one can re-write (ii) as

$$\frac{dx_2}{dt} = -(\beta - \gamma x_2)x_2 = -\theta(x_2)x_2 \qquad \text{(iii)}$$

Here deviation of the function $\theta(x_2)$ from a constant indicates the degree to which the solution to (iii) would be distinct from the simple exponential decrease. Noting that x_2 changes from approximately 0.25 on day 12.5 to approximately 0.10 on day 15.5, we can approximately put the average value of x_2: $\bar{x}$ =0.15. Clearly, (iii) would then underestimate the rate of decrease in x_2 at early times and over-estimate it at later times in the development. Nevertheless one can

see if this approximation would yield results close to those observed in mutant embryos. This assumption leads to the following solutions to simplified equations (ii) and (iii):

$$x_2\big|_{No\ Apoptosis} = \tilde{x}_2 = x_2^0 \exp(-\beta t) \qquad \text{(iv)},$$

$$x_2 = x_2^0 \exp(-\theta(\bar{x}_2)t) \qquad \text{(v)}.$$

Assuming the same initial conditions, one can get the expected ratio of the fractions of cells in the state 2 in the absence and presence of apoptotic auto-regulation:

$$\frac{\tilde{x}_2}{x_2} = \exp[(\theta(\bar{x}_2) - \beta)t] = \exp(\gamma\,\bar{x}_2 t) \qquad \text{(vi)}$$

One can see that the approximate ratio of the fractions of state 2 cells would be expected to increase exponentially with time. We can try to see if this relationship is supported by the findings in the *lpr /gld* mutant embryos, which are expected to have no Fas mediated apoptosis. If one uses Fig. 3C (main text) to estimate $\gamma \cong 1.6$ and, as above, $\bar{x}$ =0.15, then, substituting in equation (vi), the ratio of cells in state 2 in *lpr/gld* vs. wild-type embryos is predicted to increase as *exp(0.25t)*; the estimated values for *t*=1, 2 or 3, corresponding to E13.5, 14.5 or 15.5, are detailed in the table in panel '**C**'. These predicted estimates of the fold-increase in x_2 in *lpr/gld* embryos are close to our experimental findings (Fig 5C, main text, re-stated in the table in panel '**C'** above), providing support for the rough approximation made in this analysis.

Although equation (vi) suggests that the relative increase in in *lpr /gld* might continue exponentially, it is likely that other compensatory mechanisms in the embryo will prevent this from occurring. We have recently found, for example, that the TRAIL receptor, a death receptor analogous to Fas, is present at very low levels in wild-type erythroblasts, but is upregulated more than 10 fold in in *lpr* cells (M.S., unpublished observations).

Equation (vi) might thus yields an estimate of the reserve that Fas^+ erythroblasts can provide, potentially doubling x_2 by E15.5, should state 2 growth be impaired through a perturbation in the microenvironment (panel '**C**').

Section 2.6: Mixing of states 1, 2 , 3 and 4 *in vitro*

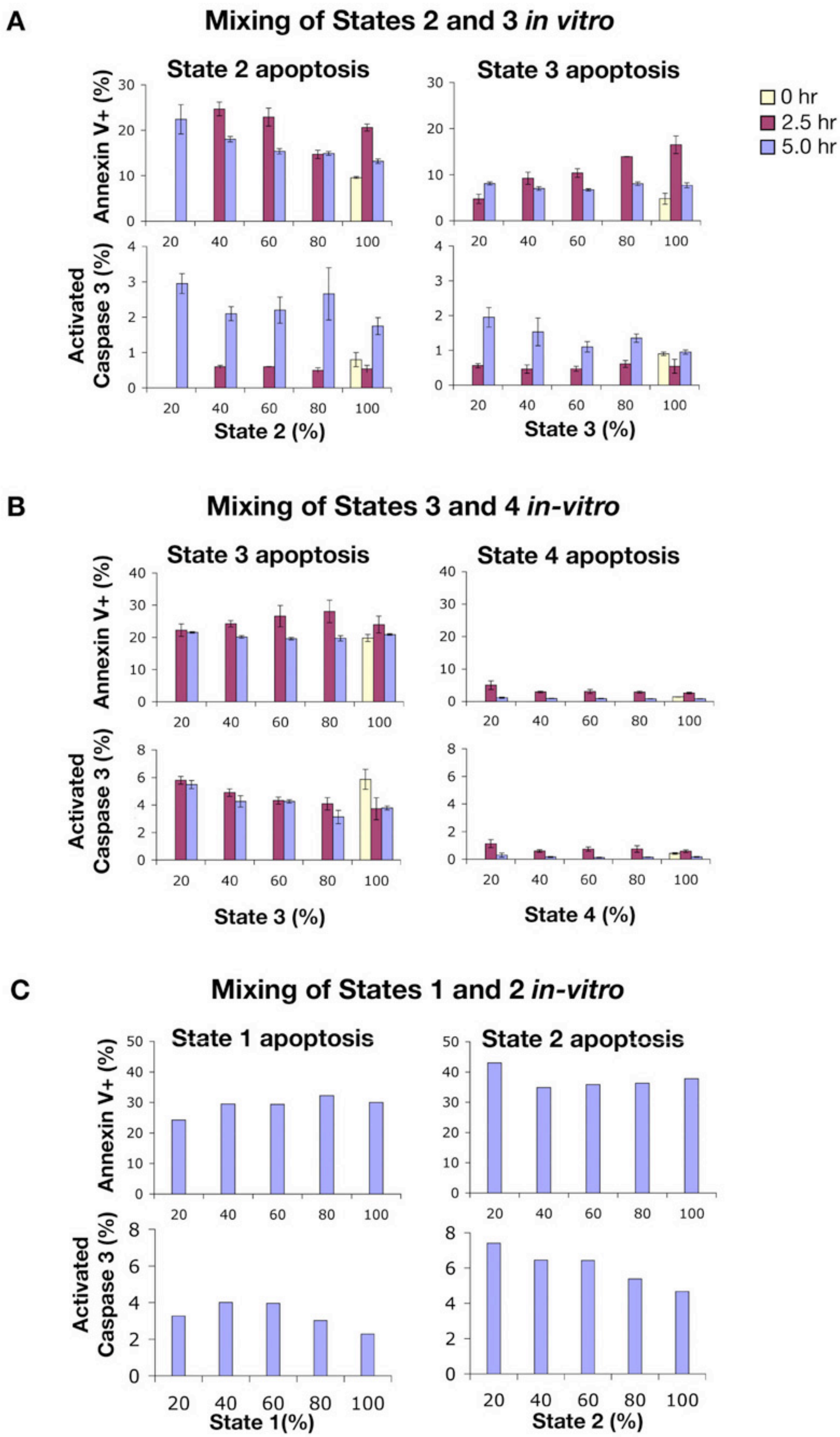

Cells in each of states 1 to 4 were sorted from freshly isolated fetal livers, using flow-cytometry. Cells were mixed at the indicated proportions and incubated at a final concentration of 2 x 10^6 cells/ml, for 0, 2.5 or 5 hours, at 37°C, in the presence of 20% serum and 0.05 U/ml Epo. At the end of the incubation period, cells were labeled for Annexin V binding and for caspase 3 activation, two independent markers of apoptosis. Each bar in panels '**A**' and '**B**' is the mean ± sem of triplicate measurements. Each bar in panel '**C**' is the mean of duplicate measurements. Only one time point was assayed in '**C**' due to the scarcity of cells in state 1.

Due to the rapid change in the relative number of cells in each state with development (main text, Fig 4A), it was difficult to obtain sufficient cells for all states at any one embryonic age. For this reason, we sorted cells from different embryonic ages as follows:

**Experiment**	**Embryonic age**
Mixing of states 3 & 4	E15.5
Mixing of states 2 & 3	E14.5
Mixing of states 1 & 2	E13.5

The data in this section is representative of 3 similar experiments.

The data suggests the following:

1. The two indicators of apoptosis give similar results (although caspase 3 activation is generally lower at t=2.5 than at t=5 hours, whereas Annexin V binding is similar at both time points).

2. The order of sensitivity of cells to apoptosis is 2>1>3>>4. This pattern is consistent with our findings *in vivo*, although state 1 apoptosis in vivo was generally lower. State 1 cells are the largest and most likely to be affected by the shear forces of cell sorting.

3. Mixing cells from different states at different proportions did not, in general, appear to produce any consistent trends in the number of cells undergoing apoptosis.

Unlike our observations *in vivo*, this *in vitro* data does not show a dependence of state 2 apoptosis on the relative concentration of cells in state 2. It is likely that the erythroblastic island architecture (section 2.2), which is disrupted during the isolation of cells for this experiment, is critical for the concentration effects seen *in vivo*. First, the erythroblastic island maintains erythroblasts in close proximity to each other, increasing their effective concentration and therefore dramatically increasing the likelihood of intercellular interactions. Second, the erythroblastic island architecture appears to maintain erythroblasts as cohorts that differentiate synchronously, thus preventing free mixing of cells once they have entered state 2. This has profound effects on the relationship between x_2 and apoptosis of state 2 cells (see main text and Figure 6).
